# Insomnia symptoms in children and adolescents: screening for sleep problems with the two-item Sleep Condition Indicator (SCI-02)

**DOI:** 10.1186/s12889-024-20310-5

**Published:** 2024-10-24

**Authors:** Gaby Illingworth, Karen L. Mansfield, Simona Skripkauskaite, Mina Fazel, Felicity Waite

**Affiliations:** 1 Department of Psychiatry, University of Oxford, Oxford, UK; 2 Department of Experimental Psychology, University of Oxford, Oxford, UK; 3 Oxford Health NHS Foundation Trust, Oxford, UK

**Keywords:** SCI-02, Insomnia, Sleep, Daytime sleepiness, Children, Adolescents, Latent profile analysis

## Abstract

**Background:**

Sleep problems are common in young people. Yet brief screening measures to identify those most in need of an intervention are lacking. This study investigated the potential of the two-item Sleep Condition Indicator (SCI-02) for screening insomnia symptoms in children and adolescents. We sought to establish whether there are distinct subgroups with different sleep profiles and whether subgroup membership varied with gender and school year group.

**Methods:**

Students (school years 5–13; typical age 9–18 years) in England completed the OxWell Student Survey in 2021. Sleep measures included: SCI-02, sleep onset latency (SOL), sleep duration, daytime sleepiness, and worry disrupting sleep. Latent profile analysis and multinomial logistic regression explored sleep profiles and predicted class membership.

**Results:**

In total, 29,304 participants answered sleep items. Of these, 95% provided binary gender (*n* = 27,802, 55% female) for analyses. Five sleep profiles emerged. The profiles, labelled “good”, “moderate”, or “poor” sleepers, vary by sleep quality – which includes time taken to fall asleep (SOL), amount of sleep (sleep duration), and the disruption of sleep due to worry. The profiles are then further differentiated by high levels of daytime sleepiness – labelled “sleepy”. “Good Sleepers” (18,355, 66%), “Moderate Sleepers” (4825, 17.4%), “Moderate Sleepy Sleepers” (1250, 4.5%), “Poor Sleepers” (1037, 3.7%) and “Poor Sleepy Sleepers” (2335, 8.4%). Probable insomnia rates (SCI-02 ≤ 2) were high in both poor sleeper profiles (70–80%) compared with other profiles (0%) and the sample overall (9%). Compared with “Good Sleepers”, all other profiles were mostly female. Daytime sleepiness – the defining characteristic of the sleepy sleeper profiles – was more common in secondary school participants than primary school.

**Conclusions:**

The SCI-02 is an efficient, two-question measure to screen for potential sleep problems in young people. Sleep disruption was high: one in ten were experiencing poor sleep. Females and adolescents appeared more vulnerable to poor sleep and daytime sleepiness. The SCI-02 has the potential for use in school and community contexts to identify children and adolescents who may benefit from support managing their sleep.

**Supplementary Information:**

The online version contains supplementary material available at 10.1186/s12889-024-20310-5.

## Background

Sleep is key to the physical, mental and cognitive health of children and young people, including academic attainment [1–4]. Impairments in sleep, such as insufficient duration and poor quality sleep, are considered risk factors for obesity [5, 6], emotional and behavioural difficulties [7] and mood deficits [8]. For example, prospective studies indicate associations between sleep problems in late childhood/adolescence and poorer mental health in adolescence/early adulthood [9–11]. Sleep plays an important role in academic performance – fundamental during these years – contributing to memory, learning and attention ( [12–14]. Notably for schools, daytime sleepiness and lapses of attention, especially for adolescents, can be an everyday consequence in the classroom [15, 16].

Insomnia is common in adolescence but may be under-diagnosed and under-treated [17, 18]. Much research documents the biopsychosocial challenges to sleep experienced by adolescents in particular, with changes to the biological regulation of sleep – including a shift in the circadian timing system (“phase delay”) and a slower accumulation of sleep pressure – contributing to a delayed sleep/wake cycle [19, 20]. This propensity to sleep late may be compounded by lifestyle and psychosocial factors, for example, electronic media use, homework, socialising, and extracurricular activities [21] and concerns over insufficient sleep are well documented [22, 23]. Insomnia is particularly evident in older adolescents and in females, with a female predominance emerging after puberty [17, 24]. Furthermore, Sivertsen and colleagues demonstrated that sleep problems in 7–9-year-old children persisted into late adolescence (aged 16–19) for approximately one in three children [25]. Given that the majority of young people attend schools, being able to assess potential sleep problems in this environment could have widespread impact. Therefore, a rapid screening tool in schools would provide an important opportunity for early intervention and potential prevention of greater problems developing.

Insomnia is defined as an experience of chronic and recurrent sleep dissatisfaction (for example, difficulty falling asleep, staying asleep, or waking up too early), and significant daytime effects [26]. The Sleep Condition Indicator (SCI) [27] is an eight-item screening tool modelled on DSM-5 diagnostic criteria for insomnia disorder [26]. The two-item short-form version of the SCI (SCI-02) [28] was developed for use in primary care. The two items evaluate the extent and frequency that sleep problems have been troubling the individual, and were chosen for the short scale in part due to their high predicted value (82% variance) of the eight-item SCI scale [27]. The SCI-02 was validated with a sample of 190,000 adults; of whom approximately 32,000 were aged 16–25 years [28]. It is brief and requires no prior knowledge of sleep disorders. Therefore, it has considerable potential as a rapid screening measure for sleep problems in young people within schools or primary care.

The present study uses data from a large cross-sectional survey in England to assess the potential of the SCI-02 as a brief screening measure of insomnia symptoms in children and adolescents. We did so by investigating whether SCI-02 scores can differentiate between different profiles of sleep behaviours. Firstly, we aimed to explore whether latent sleep profiles can be determined based on self-reported sleep onset latency (SOL), school night sleep duration, daytime sleepiness, and worry disrupting sleep, and whether SCI-02 scores differed according to sleep profile membership. Secondly, we aimed to explore whether the probability of these sleep profiles varies with gender and year group, in order to identify those most at risk, and potentially inform targeted school interventions.

## Methods

### Design and population

Data were taken from the OxWell Student Survey: a repeated cross-sectional survey that measures the mental health and wellbeing, and associated social, behavioural and emotional factors, of school-aged children and adolescents in England. The survey includes young people from state-maintained (non fee-paying) and independent (fee paying) primary schools, secondary schools, and further education (FE) colleges. In 2021, students were included from Year 5–13 (typically aged 9–18 years).

### Procedure

Schools were recruited with support from local authorities (administrative body in local government), and the OxWell Study Team provided further study information to schools that registered to take part. Schools informed parents about the survey along with instructions on how to opt-out their child. Students were shown an information video and provided with log-in details if not opted-out. Students aged 16 years and above provided online consent while those below 16 years provided online assent. The survey was completed during school time in May–July 2021. More information about the OxWell Student Survey can be found in the study protocol [29]. The study was approved by the University of Oxford Research Ethics Committee (Ref: R62366).

### Measures

Variable guides can be accessed in full on the OxWell School Survey page on the Open Science Framework (OSF) website (https://osf.io/sekhr/).

### Classification variables

#### Sleep problems

The two-item version of the Sleep Condition Indicator (SCI-02) [28] was used to screen for sleep problems. The items include: “Thinking about the past month, to what extent has poor sleep troubled you in general?” (response options: “not at all”, “a little”, “somewhat”, “much”, “very much”) and “Thinking about a typical night in the last month, how many nights a week do you have a problem with your sleep?” (response options: 0–1, 2, 3, 4, to 5–7). Each item is rated on a 5-point scale (0–4), reverse scored, and summed. Total scores range from 0–8. Higher values indicate better sleep quality whilst lower values indicate greater symptom severity, with a score of ≤ 2 reflecting DSM-5 threshold criteria for insomnia disorder.

#### Sleep behaviour

Sleep timing items were modified from the Munich Chronotype Questionnaire (MCTQ) [30] and the School Sleep Habits Survey [31, 32] (Sleep for Science webpage: http://sleepforscience.org).

#### Sleep onset latency (“SOL”)

Students were asked: “How long do you usually take to fall asleep?”. SOL was rated using a sliding scale that included five category labels (anchored at “0 mins” and at 30-minute intervals to “120 + mins (2 hours or more)”). This provided a continuous variable in decimal hours (ranging from 0.00 to 02.00), reported in minutes.

#### School night sleep duration (“sleep duration”)

Sleep duration was based on SOL, “try to sleep time”, and “wake time”. In addition to SOL, participants were asked “What time do you usually try to fall asleep on a school night?” (“try to sleep time”) and “What time do you usually wake up on a school day?” (“wake time”). Each item was rated in the survey using a sliding scale that included five category labels at 2-hourly intervals (Try to sleep time: anchored at “6pm” to “2am or later”; Wake time: anchored at “5am” to “1pm or later”). Sleep duration was calculated by the time difference between sleep onset (try to sleep time and SOL) and wake time and reported as decimal hours. A total of 191 cases with sleep durations (< 3 h) and 169 cases with sleep durations (> 12 h) considered potentially implausible were excluded [33].

#### Daytime sleepiness

Students were asked “People sometimes feel sleepy during the daytime. During your daytime activities, how much of a problem do you have with sleepiness (feeling sleepy, struggling to stay awake)?” taken from the revised School Sleep Habits Survey (see above). The item was rated using a sliding scale with five response categories and recoded into a discrete scale (ranging from 1 = “no problem at all” to 5 = “a very big problem”). Higher values indicate greater sleepiness.

#### Worry disrupting sleep

This is considered a measure of pre-sleep hyperarousal. Students were asked “How often have you been so worried about something you can not [sic.] sleep at night?” The item was rated using a sliding scale with five response categories and recoded into a discrete scale (ranging from 1 = “never” to 5 = “every night”). Higher values indicate more frequent worry.

### Predictor variables

Students reported their year group and gender, measured in younger year groups as boy, girl or prefer not to answer (Year 5–7) and in older year groups as male, female, other/prefer not to answer (Year 8–13). Year group was required to enter the survey and therefore has no missing data. Gender was treated as a binary variable (female, male) in analyses and treated as missing data if a response was either missing or provided as other/prefer not to answer. Students were grouped into three year group categories: primary school students (Year 5–6; typical age: 9–11 years), younger secondary school students (Year 7–9; typical age: 11–14 years), and older secondary school/FE students (Year 10–13; typical age: 14–18 years). FE colleges in the UK welcome students aged 16 years and older, therefore some participants in year groups 12–13 are 19 and over.

### Statistical analyses

R (version, 4.2.2; The R Foundation for Statistical Computing) was used to compute and explore descriptive statistics. Full details of inclusion criteria can be found in the pre-registration for this analysis (changes from the pre-registered analyses are outlined in the 1) [34]. To summarise, survey responses were removed when short (students spent less than 9 min on a survey, considered as survey non-starters), or when survey response times fell in non-consistent extremes (all surveys were conducted during term time, mostly within the school day, a minority were set as homework). An initial validity assessment of the SCI-02 was conducted using Spearman’s rho. Firstly, the internal reliability of the SCI-02 was examined using the correlation between the two SCI-02 items, and secondly, correlations between the SCI-02 score and other sleep measures were investigated.

Next, a Latent Profile Analysis (LPA) was conducted using Mplus [35] to examine whether there were distinct subgroups of young people with different sleep profiles. LPA is a person-oriented approach that recovers hidden groups in data [36] and obtains the probability that individuals would belong to those groups. Profiles of individuals who share similar response patterns across variables are identified and compared with other profiles [37]. The parameterization structure for the LPA models was defined based on comparison of different parameterization structures given a three-class model (Table S3). Then, a series of LPA models with one to six profiles were computed based on participants’ SOL, sleep duration, daytime sleepiness and worry disrupting sleep.

Each model was compared against the previous model(s) in an iterative process to decide on the number of latent profiles to be included. Two main indices were used, in line with existing LPA guidelines [36, 37], to determine the appropriate number of profiles and whether additional profiles in the LPA model improved the model fit: Bayesian Information Criterion (BIC), and the Vuong-Lo-Mendell-Rubin adjusted likelihood ratio test (VLMR-LRT) [38]. A lower BIC value represented the preferred model. The VLMR-LRT test assists in determining when additional profiles are not an improvement and compares significance between an estimated model versus a model with one fewer profile. A non-significant VLMR-LRT test suggests that the more parsimonious model (fewer profiles) is the better fitting and representative model. Other model fit indicators including entropy index and smallest class size were also examined as diagnostic criteria. An entropy value of > 0.80 is desired, but values between 0.60 and 0.80 were treated as acceptable. Smallest profile size was examined and *n* < 30 was considered not sufficient to support generalizability.

Once sleep profiles were identified, we conducted secondary analysis (see footnote) where we assessed whether differences in SCI-02 scores existed across the most likely profile membership. A univariate analysis of variance (ANOVA) with Bonferroni corrected post-hoc *t*-tests were conducted with SCI-02 score as the dependent variable. Lastly, whether the probability of the sleep profiles could be identified based on gender and year group was investigated. A multinomial logistic regression (mlogit package in R) [39] was carried out to predict profile membership using gender and year group as categorical predictor variables. Odds ratios (ORs) using 95% confidence intervals (CI) were extracted to determine the significance of the association. Interpretation considered that CI should not cross the value of 1 to be viewed as significant. For the LPA and secondary analysis, see Supplementary material - scripts and outputs.

## Results

### Participants

A total of 30,338 eligible students based on predefined inclusion criteria from 117 primary schools, 62 secondary schools, and one further education college, responded to the OxWell 2021 survey [34]. The final sample included participants who provided data for SCI-02 scores and at least two, and up to four, indicator variables (SOL, sleep duration, daytime sleepiness, worry disrupting sleep). A total of 814 participants (2.7% of 30,338 students) did not provide sufficient data for the SCI-02 score which left 29,524 participants remaining in the sample. Participant characteristics for those who provided SCI-02 data, with at least 2 indicator variables (SOL, sleep duration, daytime sleepiness, worry disrupting sleep) (*n* = 29,304) can be found in Table 1, while characteristics for overall sample and those missing more than 2 indicator variables (*n* = 220, 0.7% of 29,524 students) can be found in Table S1 in supplementary material. The two SCI-02 variables were significantly positively correlated (*n* = 29,304; *r*_*s*_ = 0.59, *p* < .001). Bivariate correlations between classification measures can be seen in Table 2. The SCI-02 demonstrated significant correlations with all measures with medium to large effect sizes.

**Table 1:** Sample characteristics for those who provided SCI-02 with at least 2 indicator variables (*n* = 29,304)

	SCI-02 & *≥*2 indicators (*n* = 29304)
Year group, n (%)	
Year 5–6:	
Year 5	4072 (13.9%)
Year 6	3784 (12.9%)
Total	7856 (26.8%)
Year 7–9:	
Year 7	5182 (17.7%)
Year 8	5276 (18.0%)
Year 9	5014 (17.1%)
Total	15,472 (52.8%)
Year 10–13:	
Year 10	3872 (13.2%)
Year 11	330 (1.1%)
Year 12	1501 (5.1%)
Year 13	273 (0.9%)
Total	5976 (20.4%)
Age (Years)	
Mean (SD)	12.7 (1.99)
Missing, n (%)	226 (0.8%)
Gender, n (%)	
Female	15,208 (51.9%)
Male	12,594 (43.0%)
Missing	1502 (5.1%)
SCI-02	
Mean (SD)	5.88 (2.15)
SCI-02 binary, n (%)	
Good sleep	26,329 (89.8%)
Probable insomnia	2975 (10.2%)
SOL (min)	
Mean (SD)	48.6 (34.8)
Missing, n (%)	1057 (3.6%)
Sleep duration (hrs)	
Mean (SD)	7.86 (1.58)
Missing, n (%)	3887 (13.3%)
Daytime sleepiness	
Mean (SD)	2.11 (1.04)
Missing, n (%)	714 (2.4%)
Worry disrupts sleep	
Mean (SD)	2.35 (1.11)
Missing, n (%)	1090 (3.7%)

*Note* Sleep duration is reported in decimal hours

**Table 2:** Spearman’s rho correlation matrix for classification variables

	SCI-02	Daytime sleepiness	Worry disrupts sleep	SOL	Sleep duration
SCI-02	1				
Daytime sleepiness	-0.6	1			
Worry disrupts sleep	-0.54	0.45	1		
SOL	-0.44	0.29	0.34	1	
Sleep duration	0.33	-0.33	-0.24	-0.43	1

*Note* All correlations *p* < .001

Participant characteristics for students who provided gender as female or male (*n* = 27,802) and those who did not (*n* = 1,502, 5.1% of 29,304 students) are presented in Table S2 in supplementary material. The sample with binary gender (*n* = 27,802) was included in the LPA classification and multinomial logistic regression.

### Sleep profiles

Model parameterization structure with varying means, equal variances, and varying covariances between profiles was selected. After fitting models with 2 to 6 latent classes, the 5-class model yielded the best fit. The best model fit was based on the VLMR-LRT and fall in the BIC, and it met additional diagnostic criteria including entropy index (0.86) and smallest class size (*n* = 1037). See Table S3 and Table S4 in supplementary materials.

**Fig. 1:**
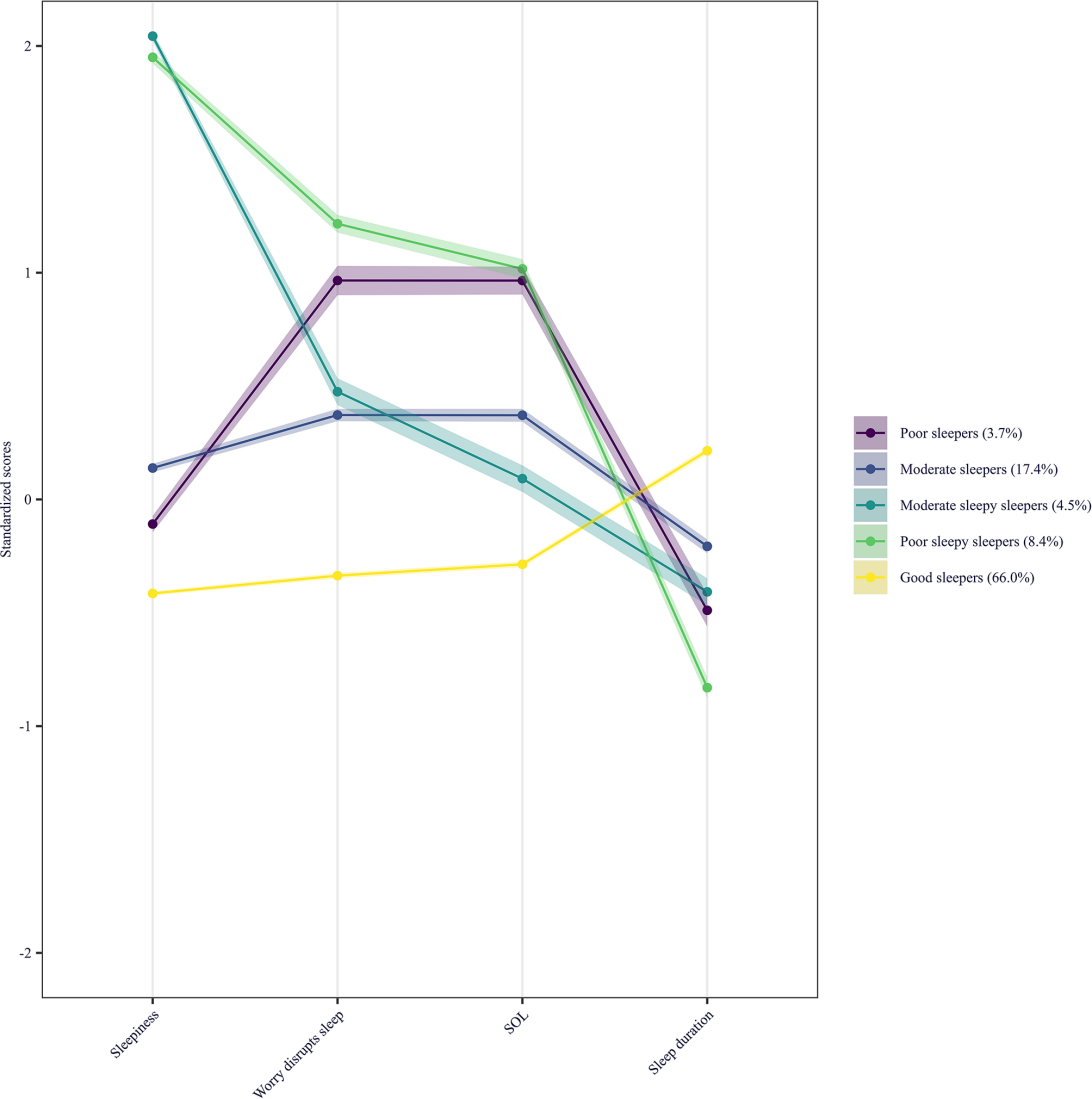
Estimated latent sleep profiles. The y-axis represents scaled and centered values for each classification variable and the shaded area represents 95% CIs

The five distinct sleep profiles that emerged from the model are presented in Fig. 1; Table 3. Sleep profiles were distinguished by sleep quality (SOL, sleep duration, worry disrupting sleep), into good, moderate and poor sleepers, with two additional profiles differentiated as “sleepy” by high levels of daytime sleepiness. The majority of participants were classed within the profile referred to as “Good Sleepers” (*n* = 18,355, 66%). This group was characterised by the lowest levels of daytime sleepiness, lowest levels of worry disrupting sleep, shortest SOL, and longest sleep duration of all profiles. The second largest profile (“Moderate Sleepers”, *n* = 4825, 17.4%), was characterised by moderate levels of sleepiness, worry disrupting sleep, SOL, and sleep duration. An additional profile (“Moderate Sleepy Sleepers”, *n* = 1250, 4.5%) was characterised by the highest levels of daytime sleepiness in the sample, with moderate worry disrupting sleep, SOL, and sleep duration.

**Table 3:** Characteristics of SCI-02, indicator and predictor variables for sleep profiles

	Good Sleepers	Moderate Sleepers	Moderate Sleepy Sleepers	Poor Sleepers	Poor Sleepy Sleepers	Overall sample
	(*n* = 18355, 66%)	(*n* = 4825, 17.4%)	(*n* = 1250, 4.5%)	(*n* = 1037, 3.7%)	(*n* = 2335, 8.4%)	(*n* = 27802)
SCI-02						
Mean (SD)	7.22 (0.76)	4.27 (0.76)	5.39 (1.24)	1.97 (0.90)	1.57 (0.99)	5.96 (2.11)
SCI-02 binary, n (%)						
Outside cutoff	18,355 (100%)	4823 (100.0%)	1250 (100%)	316 (30.5%)	473 (20.3%)	25,217 (90.7%)
Probable insomnia	0 (0%)	2 (0.0%)	0 (0%)	721 (69.5%)	1862 (79.7%)	2585 (9.3%)
Daytime sleepiness						
Mean (SD)	1.65 (0.63)	2.21 (0.66)	4.15 (0.38)	1.96 (0.59)	4.06 (0.66)	2.07 (1.02)
Missing, n (%)	374 (2.0%)	187 (3.9%)	0 (0%)	23 (2.2%)	95 (4.1%)	679 (2.4%)
Worry disrupts sleep						
Mean (SD)	1.95 (0.87)	2.73 (1.03)	2.84 (1.14)	3.38 (1.14)	3.65 (1.03)	2.32 (1.10)
Missing, n (%)	609 (3.3%)	239 (5.0%)	61 (4.9%)	41 (4.0%)	89 (3.8%)	1039 (3.7%)
SOL (min)						
Mean (SD)	37.8 (28.8)	60.6 (33.6)	51.0 (35.4)	81.0 (34.2)	82.8 (35.4)	48.0 (34.2)
Missing, n (%)	629 (3.4%)	198 (4.1%)	57 (4.6%)	40 (3.9%)	92 (3.9%)	1016 (3.7%)
Sleep duration (hrs)						
Mean (SD)	8.24 (1.40)	7.58 (1.54)	7.27 (1.56)	7.14 (1.69)	6.61 (1.62)	7.90 (1.56)
Missing, n (%)	2428 (13.2%)	649 (13.5%)	161 (12.9%)	144 (13.9%)	320 (13.7%)	3702 (13.3%)
Year group, n (%)						
Year 5–6	5242 (28.6%)	1426 (29.6%)	181 (14.5%)	367 (35.4%)	319 (13.7%)	7535 (27.1%)
Year 7–9	9837 (53.6%)	2438 (50.5%)	628 (50.2%)	448 (43.2%)	1244 (53.3%)	14,595 (52.5%)
Year 10–13	3276 (17.8%)	961 (19.9%)	441 (35.3%)	222 (21.4%)	772 (33.1%)	5672 (20.4%)
Gender, n (%)						
Female	9072 (49.4%)	2982 (61.8%)	772 (61.8%)	681 (65.7%)	1701 (72.8%)	15,208 (54.7%)
Male	9283 (50.6%)	1843 (38.2%)	478 (38.2%)	356 (34.3%)	634 (27.2%)	12,594 (45.3%)

*Note* Sleep duration is reported in decimal hours

The two profiles with the poorest sleep (together 12.1%) also differed on daytime sleepiness. Most (“Poor Sleepy Sleepers”, *n* = 2335, 8.4%) were characterised by high levels of sleepiness, the highest levels of worry disrupting sleep, longest SOL, and shortest sleep duration of all profiles. The smallest profile (“Poor Sleepers”, *n* = 1037, 3.7%) was characterised by average levels of daytime sleepiness, high worry disrupting sleep, long SOL, and relatively short sleep duration.

### Comparison of SCI-02 scores by sleep profiles

There was a significant difference between the most likely sleep profile allocation on SCI-02 score (F(4, 27797) = 39364, *p* < .001). Bonferroni corrected post-hoc *t*-tests revealed significant differences between all sleep profiles on SCI-02 score (*p*s < 0.001), listed here in order of lowest SCI-02 mean score: Poor Sleepy Sleepers (*M* = 1.57, *SD* = 0.99), Poor Sleepers (*M* = 1.97, *SD* = 0.90), Moderate Sleepers (*M* = 4.27, *SD* = 0.76), Moderate Sleepy Sleepers (*M* = 5.39, *SD* = 1.24), Good Sleepers (*M* = 7.22, *SD* = 0.76).

### SCI-02 binary classification by sleep profiles

The majority of students (*n* = 25,217, 90.7%) reported sleep quality above the cutoff (higher scores indicate better sleep quality) and not indicative of probable insomnia, while 9.3% (*n* = 2585) reported sleep problems within the cutoff (see Table 3). Probable insomnia rates (SCI-02 ≤ 2) were high in the two profiles that suggested participants were experiencing poor sleep: the highest percentage was reported by Poor Sleepy Sleepers (79.7% of profile), followed by Poor Sleepers (69.5% of profile). In contrast, Moderate Sleepers, Moderate Sleepy Sleepers and Good Sleepers reflected scores (SCI-02 > 2) that did not meet criteria for probable insomnia disorder (100% of participants in all three profiles: with the exception of two students from 4825 participants classed as Moderate Sleepers).

### Predictors of sleep profiles

**Fig. 2:**
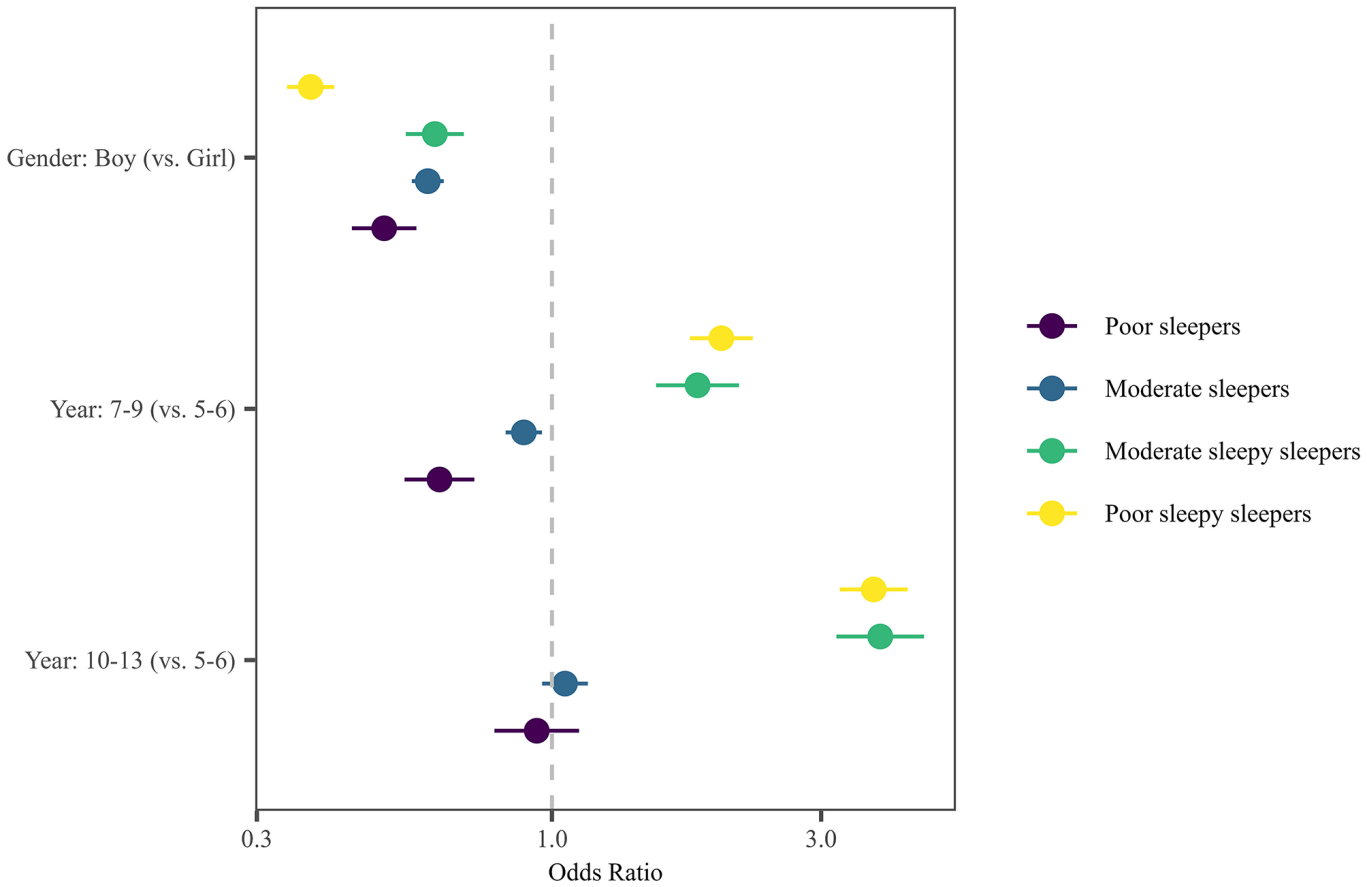
Relative odds ratios (ORs) comparing the likelihood of sleep profiles per hypothesised predictor variable (reference group: Good Sleepers). Error bars represent 95% CIs for the ORs. OR and 95% CI > 1 (to right of dotted line) indicate an increased likelihood of belonging to one of these sleep profiles compared with Good Sleepers, whereas OR and 95% CI < 1 (to left of dotted line) indicate a decreased likelihood of belonging to one of these sleep profiles compared with Good Sleepers

The multinomial logistic regression with Good Sleepers as the reference group (see Fig. 2) indicated that the likelihood of being included in different sleep profiles could be predicted by gender and year group. Considering gender, participants who were Poor Sleepers (OR 0.50, 95% CI 0.44–0.58), Moderate Sleepers (OR 0.60, 95% CI 0.56–0.64), Moderate Sleepy Sleepers (OR 0.62, 95% CI 0.55–0.70), and Poor Sleepy Sleepers (OR 0.37, 95% CI 0.34–0.41) were less likely than Good Sleepers to be male (than female). Turning to year group, participants who were Poor Sleepers (OR 0.63, 95% CI 0.55–0.73), and Moderate Sleepers (OR 0.89, 95% CI 0.83–0.96), were less likely than Good Sleepers to be in Year 7–9 than in Year 5–6. Both Moderate Sleepy Sleepers (OR 1.81, 95% CI 1.53–2.14 and OR 3.82, 95% CI 3.19–4.57, respectively) and Poor Sleepy Sleepers (OR 2.00, 95% CI 1.76–2.27 and OR 3.72, 95% CI 3.24–4.27, respectively) were more likely than Good Sleepers to be in Year 7–9 and Year 10–13 than Year 5–6.

## Discussion

One in ten young people in this study were experiencing sleep difficulties. A key step in addressing poor sleep in children and adolescents is the ability to identify those students who are experiencing sleep problems and may benefit from help managing their sleep. Findings reported here suggest that the SCI-02 is robust as a brief screening measure to assess potential sleep problems in school students. To our knowledge, this is the first study to test the SCI-02 with school-aged children and adolescents and the first to identify sleep profiles of young people based on a range of cross-sectional sleep measures using LPA. Five distinct profiles of sleep were distinguished, grouped by sleep quality (SOL, sleep duration, and worry disrupting sleep), and then further differentiated by daytime sleepiness. The majority of young people were included in the Good Sleeper profile (66%), followed by Moderate Sleepers (17.4%), Poor Sleepy Sleepers (8.4%), and Moderate Sleepy Sleepers (4.5%), while the smallest profile represented Poor Sleepers (3.7%). The SCI-02 demonstrated efficacy in differentiating profile membership.

A number of sleep-related behaviours in particular warrant discussion in relation to specific profiles. The Poor Sleeper (*M* = 7.14 h) and Poor Sleepy Sleeper (*M* = 6.61 h) profiles experienced short sleep duration in comparison with other profiles. In addition, these profiles reported the highest levels of SOL (Poor Sleeper: *M* = 81.0 min, Poor Sleepy Sleeper: *M* = 82.8 min). When considering what might impact sleep duration and SOL, Poor Sleepers and Poor Sleepy Sleepers both reported high or frequent worry disrupting sleep in comparison with other profiles (mean scores equated to on average “sometimes/most nights”), or in other words comprised students who were potentially experiencing considerable pre-sleep hyperarousal. Hyperarousal includes increased cognitive and emotional arousal and is linked to insomnia disorder [40]. Importantly, these findings suggest that pre-sleep hyperarousal and long SOL may be particularly relevant in any sleep interventions for young people. However, it is also salient to note that associations between sleep disruption and emotional difficulties are likely to be bidirectional [41] so that if one is a poor sleeper, one may be more likely to experience worry at bedtime, which might also contribute to a longer SOL.

Daytime sleepiness was particularly instrumental in the decision to interpret both moderate and poor sleepers as each comprising two distinct profiles. The profiles therefore distinguish between daytime impact, while the impact of sleep on daytime functioning and potentially engagement at school is not addressed in the SCI-02 items. Both “sleepy” profiles, on average, reported “a big problem” with sleepiness. Sleepiness is associated with worse school performance [2], therefore the inclusion of around one in eight young people in these two profiles is of particular importance given the school context of this study. Although Moderate Sleepy Sleepers comprised individuals whose sleep was labelled as moderate when considering sleep measures, these young people reported the highest levels of daytime sleepiness by profile, which merits further investigation in future research. It is plausible that this may point towards the existence of individual differences in the ability to cope with, for example, insufficient sleep or a requirement to be awake for the start of the school day that could conflict with their chronotype [42, 43]. Chronotype refers to when an individual’s circadian clock synchronises (entrains) to the 24-hour light/dark cycle [44] and is defined at a behavioural level by their preferred sleep/wake timing. The phase delay in circadian timing is reflected in the adolescent tendency to be an “evening chronotype”, which may be incompatible with school start times if they have to wake earlier than their biologically appropriate time. This explanation fits with previous findings that greater eveningness preference was associated with greater daytime sleepiness [45].

A sizeable minority of 9.3% children and young people in this sample reported sleep problems that were indicative of probable insomnia disorder according to the cutoff designated by the SCI-02 [28]. Similar rates were found in a longitudinal study with a younger age group (5–14 years; *n* = 1993) in Australia which showed that one in ten young people experienced higher levels of sleep problems from childhood to adolescence [46]. When assessing the ability of the SCI-02 to distinguish between sleep profiles, significant differences in the SCI-02 score were found between all profiles in an order consistent with how these were categorised. Poor Sleepy Sleepers and Poor Sleepers both had mean SCI-02 scores inside the cutoff for probable insomnia (≤ 2) while mean scores provided by Moderate Sleepers, Moderate Sleepy Sleepers and Good Sleepers would not be considered to indicate probable insomnia. Furthermore, the SCI-02 showed considerable promise when used as a binary variable in differentiating between those with potential sleep problems and those with better sleep. Probable insomnia rates were recorded as 69.5% of Poor Sleepers and 79.7% of Poor Sleepy Sleepers while clear-cut findings emerged for the remaining profiles with 100% of Moderate Sleepers, Moderate Sleepy Sleepers and Good Sleepers not meeting the criteria for insomnia disorder.

Binary gender differences emerged when considering all sleep profiles. All sleep profiles other than Good Sleepers, were predominantly female. The implication that females may be more vulnerable to sleep problems, given their over-representation in the Poor Sleeper and Poor Sleepy Sleeper profiles, is in line with previous findings that females are more predisposed to insomnia disorder and insomnia symptoms than males in adolescence [17, 18, 24]. Furthermore, females were more likely to be in the profiles categorised by high levels of daytime sleepiness. This supports the emergence of a female predominance in excessive daytime sleepiness found in a community-based study in Hong Kong [47]. Therefore, taken together these findings suggest that females are not only more likely to experience poor sleep but also to experience daytime sleepiness.

The younger year group (Year 5–6) was proportionately least likely to be assigned to the Moderate Sleepy Sleeper and Poor Sleepy Sleeper profiles. Notably, adolescents in Year 7–9 and Year 10–13 were more likely to be in the Poor Sleepy Sleeper profile, therefore potentially struggling with sleep in all reported measures as well as experiencing a knock-on effect in daytime functioning demonstrated by high levels of sleepiness. This fits with what we know about challenges to adolescent sleep patterns, for example from the adolescent phase delay when circadian rhythms may be misaligned with school start times [20, 48]. In addition, co-occurring psychosocial factors may contribute to poor sleep in secondary school students when compared with primary school children. For example, parents are more likely to set children’s bedtimes, whereas greater autonomy over bedtimes and activities/routines (including electronic device use) prior to sleep, may contribute to later bedtimes and shorter sleep duration in adolescence [49, 50]. Moreover, sleep disorders often emerge during adolescence, with insomnia being the most common [51]. Children in primary school were less likely to report daytime sleepiness. This is in line with previous findings that daytime sleepiness increases in adolescence compared with childhood [52, 53]. Brain reorganisation during adolescence, potentially driven by synaptic pruning (process of synaptic elimination that increases the efficiency of the neural network), has been proposed as a contributor to daytime sleepiness during this period [52].

A number of limitations should be borne in mind when considering these findings and their generalisability to other populations of young people. Firstly, the SCI-02 measure has not been formally validated with adolescents, and this is a purely exploratory, cross-sectional study investigating the association between SCI-02 and self-reported sleep characteristics in one sample. When considering the older secondary school students (Year 10–13), the majority included in this group were in Year 10 (64.8%, 13.2% of sample) while a smaller proportion (35.2%) were in Year 11–13 combined (Table 1). An inverse relationship between pediatric nighttime sleep duration and age has been demonstrated [54], with older adolescents going to bed later on school nights and having a shorter sleep duration [55]. Given developmental patterns of sleep, it may be that our findings have underestimated the sleep problems experienced in Year 10–13 as a consequence of this under-representation of older year groups. The sleep profiles described in this study were based on students who provided a binary gender and did not include 1502 students (5.1% of sample) who responded as “other / prefer not to answer”, or for whom gender data were missing. Our data do not provide sufficient detail to assess sleep for students with non-binary gender. Further research is recommended including older school-aged adolescents so that the experiences of those in Year 11–13 can be more fully explored in relation to the sleep profiles developed in this study, as well as assessment of any sleep problems using the SCI-02.

## Conclusions

The SCI-02 demonstrates promise as a brief measure to identify young people experiencing poor sleep. This study suggests the measure can differentiate between those with poor sleep and those with moderate or good sleep based on sleep profiles developed from a large community sample of school-aged children and adolescents. Notably, around one in ten are experiencing sleep problems and may benefit from support managing their sleep. Not all experience daytime sleepiness, making them harder to identify without screening. Our findings indicate that females and adolescents are at greater risk for both poor sleep and daytime sleepiness. Determining how best to utilise the SCI-02 in school, community and secondary care contexts will enable better identification of sleep difficulties in child and adolescent populations. Given its brevity and ease of use, this two-item measure has the potential to be easily adopted in a busy school-based setting. Assessing young people’s sleep using this tool may facilitate a simple evaluation of sleep and any changes over time. Furthermore, sleep measurement with the SCI-02 may help evaluate the effectiveness of intervention strategies and support provided, and have considerable interest for researchers and educators working in schools.

## Electronic supplementary material

Below is the link to the electronic supplementary material.

Supplementary Material 1

Supplementary Material 2

## Data Availability

Fully deidentified extracts of the data can be provided to academic research collaborators upon reasonable request after a review process by the research team to ensure that uses of the data fall under the remit of the intended purposes set out in the privacy information and to prevent duplication of analyses. The data are not publicly available because of ethical and information governance restrictions. The full list of questions as well as other details are available on a project-specific OxWell Open Science Framework website along with the study protocol (https://osf.io/sekhr/). Full data dictionaries can be made available upon approval for access to data extracts.

## Footnotes

1 The statistical analysis of this study was pre-registered via Open Science Framework (OSF; https://osf.io/jdyzg). Convergence of an initial LPA model was not achieved when six sleep measures were included (SCI-02 score, SOL, sleep duration, sleep efficiency, daytime sleepiness and worry disrupting sleep). In the event of model non-convergence, the pre-registration stated that the outlined list of classification variables would be reduced step-by-step based on theoretical reasons. Sleep efficiency was named as the first classification variable to be excluded from analysis in this event. In the analysis reported here, sleep efficiency was removed due to a potential issue of collinearity and as it derived in part from two sleep classification measures also included in the LPA (“sleep duration” and “sleep onset latency”). The SCI-02 was also not included as a classifier in the LPA enabling us to investigate the predictive ability of the SCI-02 and its ability to differentiate profile membership.

## Abbreviations

SCI-02: Two-item Sleep Condition Indicator
SOL: Sleep onset latency
LPA: Latent Profile Analysis
BIC: Bayesian Information Criterion
VLMR- LRT: Vuong-Lo-Mendell-Rubin adjusted likelihood ratio test
OR: Odds ratio
CI: Confidence interval
