# Supplementary material for: Insomnia symptoms in children and adolescents: screening for sleep problems with the two-item Sleep Condition Indicator (SCI-02)
Source: BMC Public Health. 2024 Oct 24;24:2957. doi: 10.1186/s12889-024-20310-5 (PMC11515297; doi:10.1186/s12889-024-20310-5)
Supplement: Supplementary file 1 — Supplementary Material 1 [file 12889_2024_20310_MOESM1_ESM.docx]

**Supplementary material**

**Table S1.** Sample characteristics for those who provided SCI-02 (*n* = 29,524) with at least 2 indicator variables (*n* = 29,304) and missing more than 2 indicator variables (*n* = 220)

|  | SCI-02 & >2 indicators  (*n* = 29304) | SCI-02 & <2 indicators  (*n* = 220) | Overall  (*n* = 29524) |
| --- | --- | --- | --- |
| Year group, n (%) |  |  |  |
| Year 5–6: |  |  |  |
| Year 5 | 4072 (13.9%) | 28 (12.7%) | 4100 (13.9%) |
| Year 6 | 3784 (12.9%) | 29 (13.2%) | 3813 (12.9%) |
| Total | 7856 (26.8%) | 57 (25.9%) | 7913 (26.8%) |
| Year 7–9: |  |  |  |
| Year 7 | 5182 (17.7%) | 27 (12.3%) | 5209 (17.6%) |
| Year 8 | 5276 (18.0%) | 38 (17.3%) | 5314 (18.0%) |
| Year 9 | 5014 (17.1%) | 35 (15.9%) | 5049 (17.1%) |
| Total | 15472 (52.8%) | 100 (45.5%) | 15572 (52.7%) |
| Year 10–13: |  |  |  |
| Year 10 | 3872 (13.2%) | 43 (19.5%) | 3915 (13.3%) |
| Year 11 | 330 (1.1%) | 2 (0.9%) | 332 (1.1%) |
| Year 12 | 1501 (5.1%) | 14 (6.4%) | 1515 (5.1%) |
| Year 13 | 273 (0.9%) | 4 (1.8%) | 277 (0.9%) |
| Total | 5976 (20.4%) | 63 (28.6%) | 6039 (20.5%) |
| Age (Years) |  |  |  |
| Mean (SD) | 12.7 (1.99) | 13.0 (2.09) | 12.7 (1.99) |
| Missing, n (%) | 226 (0.8%) | 6 (2.7%) | 232 (0.8%) |
| Gender, n (%) |  |  |  |
| Female | 15208 (51.9%) | 111 (50.5%) | 15319 (51.9%) |
| Male | 12594 (43.0%) | 98 (44.5%) | 12692 (43.0%) |
| Missing | 1502 (5.1%) | 11 (5.0%) | 1513 (5.1%) |
| SCI-02 |  |  |  |
| Mean (SD) | 5.88 (2.15) | 5.40 (2.14) | 5.88 (2.15) |
| SCI-02 binary, n (%) |  |  |  |
| Good sleep | 26329 (89.8%) | 192 (87.3%) | 26521 (89.8%) |
| Probable insomnia | 2975 (10.2%) | 28 (12.7%) | 3003 (10.2%) |
| SOL (min) |  |  |  |
| Mean (SD) | 48.6 (34.8) | 43.2 (34.2) | 48.6 (34.8) |
| Missing, n (%) | 1057 (3.6%) | 203 (92.3%) | 1260 (4.3%) |
| Sleep duration (hrs) |  |  |  |
| Mean (SD) | 7.86 (1.58) | NA (NA) | 7.86 (1.58) |
| Missing, n (%) | 3887 (13.3%) | 220 (100%) | 4107 (13.9%) |
| Daytime sleepiness |  |  |  |
| Mean (SD) | 2.11 (1.04) | 2.23 (1.02) | 2.11 (1.04) |
| Missing, n (%) | 714 (2.4%) | 126 (57.3%) | 840 (2.8%) |
| Worry disrupts sleep |  |  |  |
| Mean (SD) | 2.35 (1.11) | 2.62 (1.28) | 2.35 (1.11) |
| Missing, n (%) | 1090 (3.7%) | 141 (64.1%) | 1231 (4.2%) |

*Note.* Sleep duration is reported in decimal hours.

**Table S2**. Sample characteristics for classification and prediction sample (students who provided gender as female or male, *n* = 27,802), those who did not provide binary gender (*n* = 1,502) and overall sample (*n* = 29,304)

|  | Gender (binary)  (*n* = 27802) | Gender (other, prefer not to answer, missing)  (*n* = 1502) | Overall  (*n* = 29304) |
| --- | --- | --- | --- |
| Year group, n (%) |  |  |  |
| Year 5–6 | 7535 (27.1%) | 321 (21.4%) | 7856 (26.8%) |
| Year 7–9 | 14595 (52.5%) | 877 (58.4%) | 15472 (52.8%) |
| Year 10–13 | 5672 (20.4%) | 304 (20.2%) | 5976 (20.4%) |
| Gender, n (%) |  |  |  |
| Female | 15208 (54.7%) | 0 (0%) | 15208 (51.9%) |
| Male | 12594 (45.3%) | 0 (0%) | 12594 (43.0%) |
| Missing | 0 (0%) | 1502 (100%) | 1502 (5.1%) |
| SCI-02 |  |  |  |
| Mean (SD) | 5.96 (2.11) | 4.50 (2.49) | 5.88 (2.15) |
| SCI-02 binary, n (%) |  |  |  |
| Good sleep | 25217 (90.7%) | 1112 (74.0%) | 26329 (89.8%) |
| Probable insomnia | 2585 (9.3%) | 390 (26.0%) | 2975 (10.2%) |
| SOL (min) |  |  |  |
| Mean (SD) | 48.0 (34.2) | 69.0 (39.0) | 48.6 (34.8) |
| Missing, n (%) | 1016 (3.7%) | 41 (2.7%) | 1057 (3.6%) |
| Sleep duration (hrs) |  |  |  |
| Mean (SD) | 7.90 (1.56) | 7.07 (1.76) | 7.86 (1.58) |
| Missing, n (%) | 3702 (13.3%) | 185 (12.3%) | 3887 (13.3%) |
| Daytime sleepiness |  |  |  |
| Mean (SD) | 2.07 (1.02) | 2.74 (1.21) | 2.11 (1.04) |
| Missing, n (%) | 679 (2.4%) | 35 (2.3%) | 714 (2.4%) |
| Worry disrupts sleep |  |  |  |
| Mean (SD) | 2.32 (1.10) | 2.96 (1.21) | 2.35 (1.11) |
| Missing, n (%) | 1039 (3.7%) | 51 (3.4%) | 1090 (3.7%) |

*Note.* Sleep duration is reported in decimal hours.

**Table S3**. Model fit indices for model parameterization decision (based on a 3-profile model)

|  |  |  | VLMR-LRT | |  |  |  |  |  |
| --- | --- | --- | --- | --- | --- | --- | --- | --- | --- |
| *P* | BIC | LL | -2LL | *p* | Entropy | Smallest class | Δχ^2^_SB_ | Δ*df* | *p* |
| Model 1: (Default) Varying means, equal variance, zero covariance | | | | | | |  |  |  |
| 18 | 258131 | -128973.34 | 5936.13 | <.001 | 0.863 | 11% |  |  |  |
| Model 2: Varying means, varying variances, zero covariance | | | | | | |  |  |  |
|  | *Correct model computation or model convergence could not be achieved.* | | | | | |  |  |  |
| Model 3: Varying means, equal variance and equal covariance | | | | | | |  |  |  |
| 24 | 253790 | -126772.38 | 2922.87 | <.001 | 0.870 | 11% | 4451.77 | 6 | <.001 |
| Model 4: Varying means, varying variances, and equal covariances | | | | | | |  |  |  |
| 32 | 244875 | -122273.54 | 3849.07 | <.001 | 0.664 | 27% | 20910.74 | 14 | <.001 |
| **Model 5: Varying means, equal variances, and varying covariances** | | | | | | |  |  |  |
| 36 | 252357 | **-125994.42** | **3538.99** | **<.001** | **0.854** | **11%** | 4409.30 | **18** | <.001 |
| Model 6: Varying means, varying variances and varying covariances | | | | | | |  |  |  |
| 44 | 243929 | -121739.38 | 3424.50 | <.001 | 0.651 | 25% | 16021.51 | 26 | <.001 |

*Note.* Bold text indicates model met fit criteria. P = number of parameters; BIC = Bayesian information criterion; LL = log-likelihood; -2LL = 2 times the log-likelihood difference; VLMR-LRT = Vuong-Lo-Mendell- Rubin adjusted likelihood ratio test. Satorra-Bentler Chi-Square change test (Δχ^2^_SB_) was used to compare the model fit of each parameterization model with the default model. Model 5 parameterization structure was chosen to carry forward on the basis of BIC, log-likelihood, entropy, and Δχ^2^_SB_ test in comparison to the default model and in comparison to the more constrained Model 3 (Δχ^2^_SB_(12)= 1015.36, *p*<.001).

**Table S4**. Model fit statistics and diagnostic criteria for models with 2 to 6 classes

|  | *P* | BIC | LL | VLMR- LRT | | Entropy | Smallest class |
| --- | --- | --- | --- | --- | --- | --- | --- |
|  |  |  |  | -2LL | *p* |  |  |
| 2 Class | 25 | 255784 | -127763.91 | 10278.80 | <.001 | .878 | 23% |
| 3 Class | 36 | 252357 | -125994.42 | 3538.99 | <.001 | .854 | 11% |
| 4 Class | 47 | 249901 | -124709.90 | 2569.03 | <.001 | .855 | 5% |
| **5 Class** | **58** | **248761** | **-124083.50** | **1252.79** | **<.001** | **.860** | **4%** |
| 6 Class* | 69 | 247466 | -123380.20 | -25874.29 | 1 | .842 | 3% |

*Note.* Bold text indicates model met fit criteria. P = number of parameters; BIC = Bayesian information criterion; LL = log-likelihood; -2LL = 2 times the log-likelihood difference; VLMR-LRT = Vuong-Lo-Mendell-Rubin adjusted likelihood ratio test.

* The 6 Class model did not achieve convergence at global maxima even with increased starting values (8000/2000).
