# Supplementary material for: Insomnia symptoms in children and adolescents: screening for sleep problems with the two-item Sleep Condition Indicator (SCI-02)
Source: BMC Public Health. 2024 Oct 24;24:2957. doi: 10.1186/s12889-024-20310-5 (PMC11515297; doi:10.1186/s12889-024-20310-5)

Scripts & Outputs
"Insomnia symptoms in children and adolescents: Screening for sleep problems with the Two-item Sleep Condition Indicator (SCI-02)"

Latent Profile Analysis (LPA) in Mplus

Step 1: Model Parameterization Structure

The parametrization structure for the LPA models was defined based on comparison of different parametrization structures given three class model.

Parametrization Model 1 (Default) - Equal variances and zero covariances

```
Mplus VERSION 8.7
MUTHEN & MUTHEN
07/11/2023  11:26 AM

INPUT INSTRUCTIONS

  TITLE: LPA 3 profile syntax: M1 free means, constrained variance, zero covariance
  DATA:
  FILE IS mplus2.csv;
  ! Specifies file location for data file. Make sure data is in format appropriate for Mplus
  ! per Mplus manual. This data file is in individual format (one row of data per participan
  VARIABLE:
  NAMES ARE ID SCI_02 SCI_02B slpnssN worryN sol slpDur gender genderN yrgrp yrgrpN SCI_02C
  slpnssNC worryNC solC slpDurC;

  ! All variables included in data file should be named here.
  USEVARIABLES ARE slpnssNC worryNC solC slpDurC;
  ! Only variables intended for use in the analysis should be listed here
  IDVARIABLE IS ID;
  CLASSES = c (3);
  ! This is where you instruct Mplus on how many classes/profiles are being estimated. Initi
  ! model contains only one class/profile, thus it would be CLASSES = c (1). Above specifies
  !profiles, and for each further iterative models the number in parentheses increases by on
  !three profiles/classes would be c (3), and so on.
  MISSING ARE ALL(9999);
  ! Used to communicate how missing data is coded in data file. Here shown with a "." which
  ! all that is included in each cell with missing data in the data file
  ANALYSIS:
  TYPE = MIXTURE;
  ! LPA is a version of mixture modeling, and this instructs Mplus to analyze in this way
  ESTIMATOR = MLR;
  !FIML robust to non-normal data
  STARTS = 1000 250;
  STITERATIONS = 500;
  ! Default number of starts for each step of the ML estimation. First STARTS value specifie
  !number of unique start values to start with, the 250 represents the 250 best unique start
  !carrying forward to completion. The STITERATIONS specifies the number of ML iteration
  !steps for those 250 selected start values to go through to be able to converge. This is a
  !maximum number of iteration; if a model converges in less than 500 iterations it will sto
  !before reaching 500 iterations.
  !These values can be increased … see "Four-Profile Final Model with Covariate Analysis
  !Syntax" for an example.
  LRTSTARTS = 2 1 50 10;
  LRTBOOTSTRAP = 250;
  !The above start values are for the defaults for the LRT statistic being run to compare th
  !model fit with the model fit of a model with one less class (k-1). The BOOTSTRAP statemen
  !specifies the number of bootstrap draws to inform Mplus' bootstrapped LRT results.
  MODEL:
  !For a default Mplus model the LPA model does not need to be specified. However, it can be
  !The model can also be modified from the Mplus default of estimating the indicator means
  !(uniquely across profiles) and variances (constrained across profiles), as well as the la
  !profile mean.
  %OVERALL%
  [slpnssNC worryNC solC slpDurC]; !estimates the indicators means for each profile. Without
  !the means are freely estimated in each profile, not constrained.
  slpnssNC worryNC solC slpDurC; !Label Var1-Var5 constrains the estimates of the variances
  !profiles to be equal.

  OUTPUT:
  TECH11 TECH14;
  ! TECH1 provides parameter specifications and starting values for the analysis
  ! TECH8 provides optimization history for this analysis type
  !TECH11 provides LRT results
  !TECH14 provides bootstrapped LRT test
  PLOT: SERIES=slpnssNC worryNC solC slpDurC(*);
  TYPE=PLOT3;
  SAVEDATA:
  FILE IS Step1_LPA3M1.dat;
  ! Tells Mplus where to save the output files from the analysis
  SAVE = CPROBABILITIES;
  ! The above command lines are to save the most likely profile membership for each particip
  ! and the posterior probabilities for their membership in each latent profile.

*** WARNING in MODEL command
  All variables are uncorrelated with all other variables within class.
  Check that this is what is intended.
```

Scripts & Outputs
"Insomnia symptoms in children and adolescents: Screening for sleep problems with the Two-item Sleep Condition Indicator (SCI-02)"

```
LPA 3 profile syntax: M1 free means, constrained variance,
zero covariance

SUMMARY OF ANALYSIS

Number of groups                                      1
Number of observations                            27802

Number of dependent variables                         4
Number of independent variables                       0
Number of continuous latent variables                 0
Number of categorical latent variables                1

Observed dependent variables

  Continuous
   SLPNSSNC    WORRYNC     SOLC       SLPDURC

Categorical latent variables
   C

Variables with special functions

  ID variable          ID

Estimator                                           MLR
Information matrix                             OBSERVED
Optimization Specifications for the Quasi-Newton Algorithm
for
Continuous Outcomes
  Maximum number of iterations                      100
  Convergence criterion                       0.100D-05
Optimization Specifications for the EM Algorithm
  Maximum number of iterations                      500
  Convergence criteria
    Loglikelihood change                      0.100D-06
    Relative loglikelihood change             0.100D-06
    Derivative                                0.100D-05
Optimization Specifications for the M step of the EM
Algorithm for
Categorical Latent variables
  Number of M step iterations                         1
  M step convergence criterion                0.100D-05
  Basis for M step termination                ITERATION
Optimization Specifications for the M step of the EM
Algorithm for
Censored, Binary or Ordered Categorical (Ordinal),
Unordered
Categorical (Nominal) and Count Outcomes
  Number of M step iterations                         1
  M step convergence criterion                0.100D-05
  Basis for M step termination                ITERATION
  Maximum value for logit thresholds                 15
  Minimum value for logit thresholds                -15
  Minimum expected cell size for chi-square     0.100D-
01
Maximum number of iterations for H1                2000
Convergence criterion for H1                  0.100D-03
Optimization algorithm                              EMA
Random Starts Specifications
  Number of initial stage random starts            1000
  Number of final stage optimizations               250
  Number of initial stage iterations                500
  Initial stage convergence criterion         0.100D+01
  Random starts scale                         0.500D+01
  Random seed for generating random starts            0

Input data file(s)
 mplus2.csv
Input data format  FREE

SUMMARY OF DATA

    Number of missing data patterns             4
    Number of y missing data patterns           4
    Number of u missing data patterns           0

COVARIANCE COVERAGE OF DATA

Minimum covariance coverage value   0.100

    PROPORTION OF DATA PRESENT FOR Y


          Covariance Coverage
            SLPNSSNC      WORRYNC       SOLC
SLPDURC
            ________      ________      ________      ________
 SLPNSSNC       1.000
 WORRYNC        1.000         1.000
 SOLC           0.976         0.976         0.976
 SLPDURC        0.963         0.963         0.940         0.963

UNIVARIATE SAMPLE STATISTICS

    UNIVARIATE HIGHER-ORDER MOMENT
DESCRIPTIVE STATISTICS

      Variable/         Mean/      Skewness/   Minimum/ % with
Percentiles
     Sample Size      Variance    Kurtosis    Maximum
Min/Max      20%/60%    40%/80%    Median

    SLPNSSNC             0.933       0.086       0.000   27.10%
0.000      1.000      1.000
         27802.000       0.471      -0.878       2.000   20.40%
1.000      2.000
    WORRYNC              0.000      -1.036      -2.825    1.71%
-0.928      0.020      0.495
         27802.000       1.000       0.165       0.969   28.55%
0.495      0.969
    SOLC                 0.000       0.967      -1.054   31.63%     -
1.054     -0.072     -0.072
         27123.000       1.000       0.467       2.875    2.77%
-0.072      0.910
    SLPDURC              0.000       0.568      -1.205   25.70%
-1.205     -0.292     -0.292
         26763.000       1.000      -0.470       2.445    3.44%
-0.292      0.620


RANDOM STARTS RESULTS RANKED FROM THE BEST
TO THE WORST LOGLIKELIHOOD VALUES

Final stage loglikelihood values at local maxima, seeds, and
initial stage start numbers:

         -128973.336  780698           337
         -128973.336  569338           755
         -128973.336  699810           571
         -128973.336  298553           773
         -128973.336  232559           136
         -128973.336  628143           854
         -128973.336  635245           121
         -128973.336  27071            15
         -128973.336  810705           626
         -128973.336  545108           667
         -128973.336  926283           269
```

-128973.336	268896	124
-128973.336	850112	922
-128973.336	297518	166
-128973.336	738393	619
-128973.336	214681	824
-128973.336	797594	234
-128973.336	425929	508
-128973.336	200041	810
-128973.336	609089	241
-128973.336	853781	716
-128973.336	914505	838
-128973.336	399848	220
-128973.336	444228	860
-128973.336	209031	632
-128973.336	351807	617
-128973.336	937885	426
-128973.336	646573	741
-128973.336	327140	678
-128973.336	996231	310
-128973.336	264901	634
-128973.336	710445	792
-128973.336	568859	49
-128973.336	435506	988
-128973.336	573096	20
-128973.336	85734	411
-128973.336	21132	351
-128973.336	563002	360
-128973.336	538872	949
-128973.336	248742	556
-128973.336	686482	215
-128973.336	705224	953
-128973.336	43523	297
-128973.336	950604	172
-128973.336	499150	216
-128973.336	668003	647
-128973.336	357866	968
-128973.336	284716	713
-128973.336	285380	1
-128973.336	224950	455
-128973.336	67009	564
-128973.336	490123	995
-128973.336	462228	298
-128973.336	344422	296
-128973.336	72662	729
-128973.336	291112	645
-128973.336	805768	879
-128973.336	68850	462
-128973.336	921023	782
-128973.336	710154	831
-128973.336	376411	473
-128973.336	574942	558
-128973.336	804660	260
-128973.336	393232	152
-128973.336	595759	997
-128973.336	364676	27
-128973.336	629320	222
-128973.336	350608	334
-128973.336	700349	401
-128973.336	167409	772
-128973.336	195763	358
-128973.336	147440	514
-128973.336	603842	61
-128973.336	608849	224
-128973.336	415931	10
-128973.336	652266	490
-128973.336	900631	774
-128973.336	231281	542
-128973.336	922596	456
-128973.336	154575	539

-128973.336	383902	673
-128973.336	887676	22
-128973.336	351622	551
-128973.336	402699	604
-128973.336	130011	587
-128973.336	72344	897
-128973.336	474357	789
-128973.336	318177	748
-128973.336	458181	189
-128973.336	483369	270
-128973.336	92564	583
-128973.336	288738	940
-128973.336	535063	329
-128973.336	475419	987
-128973.336	966499	963
-128973.336	424223	900
-128973.336	848890	95
-128973.336	195353	225
-128973.336	117391	710
-128973.336	326091	759
-128973.336	965994	396
-128973.336	966603	919
-128973.336	89970	223
-128973.336	865906	641
-128973.336	891347	504
-128973.336	484406	421
-128973.336	849670	347
-128973.336	507218	613
-128973.336	900921	984
-128973.336	970689	266
-128973.336	810594	845
-128973.336	192191	629
-128973.336	314034	513
-128973.336	939021	8
-128973.336	438144	271
-128973.336	341960	685
-128973.336	137888	901
-128973.336	281558	184
-128973.336	392751	480
-128973.336	850545	357
-128973.336	928287	197
-128973.336	264081	186
-128973.336	629720	926
-128973.336	572637	989
-128973.336	644297	340
-128973.336	323588	826
-128973.336	210139	991
-128973.336	215353	164
-128973.336	809240	543
-128973.336	432513	803
-128973.336	298275	418
-128973.336	23012	352
-128973.336	979450	913
-128973.336	118421	139
-128973.336	857122	889
-128973.336	669639	699
-128973.336	107446	12
-128973.336	252949	487
-128973.336	417035	149
-128973.336	343926	624
-128973.336	39136	226
-128973.336	696830	668
-128973.336	597614	284
-128973.336	213532	503
-128973.336	551340	766
-128973.336	22075	659
-128973.336	519357	559
-128973.336	404426	344
-128973.336	79945	395

-128973.336	164305	128
-128973.336	760599	832
-128973.336	387701	275
-128973.336	792993	859
-128973.336	869564	869
-128973.336	638577	769
-128973.336	407168	44
-128973.336	863094	147
-128973.336	217744	326
-128973.336	782821	272
-128973.336	805935	615
-128973.336	173191	422
-128973.336	81233	825
-128973.336	749453	33
-128973.336	724087	925
-128973.336	752769	253
-128973.336	702492	718
-128973.336	547702	767
-128973.336	988761	475
-128973.336	224151	973
-128973.336	679448	937
-128973.336	724519	333
-128973.336	246261	38
-128973.336	977800	853
-128973.336	220454	288
-128973.336	648555	113
-128973.336	413564	535
-128973.336	476295	969
-128973.336	138695	783
-128973.336	860102	495
-128973.336	781190	219
-128973.336	341041	34
-128973.336	127362	757
-128973.336	418686	338
-128973.336	152496	123
-128973.336	46437	153
-128973.336	830292	527
-128973.336	211281	292
-128973.336	995913	787
-128973.336	590834	785
-128973.336	192071	142
-128973.336	188640	451
-128973.336	928624	981
-128973.336	671390	814
-128973.336	140849	515
-128973.336	606576	151
-128973.336	887580	493
-128973.336	497522	502
-128973.336	302046	863
-128973.336	626208	698
-128973.336	505244	582
-128973.336	602032	648
-128973.336	802256	477
-128973.336	971853	402
-128973.336	589483	950
-128973.336	373702	669
-128973.336	534864	307
-128973.336	650371	14
-128973.336	824126	287
-128973.336	781489	627
-128973.336	669634	335
-128973.336	526324	178
-128973.336	377584	630
-128973.336	271809	846
-128973.336	848331	137
-128973.336	712702	684
-128973.336	451258	848
-128973.336	856612	700
-128973.336	278661	674
-128973.336	871851	257
-128973.336	76974	16
-128973.336	561664	392
-128973.336	202790	198
-128973.336	150531	154
-128973.336	237332	661
-128973.336	252346	528
-128973.336	62835	642
-128973.336	312754	562
-128973.336	475420	71
-128973.336	704798	530
-128973.336	636396	168
-128973.336	299977	956
-128973.336	456213	160
-128973.336	79212	517
-128973.336	437181	135
-128973.336	640833	434
-128973.336	76451	211
-128973.336	529496	343
-128973.336	441191	361
-128973.336	267983	228
-128973.336	246794	801
-128973.336	225995	847
-128973.336	568405	233
-128973.336	575700	100
-128973.336	345974	622
-128973.336	726744	939
-128973.336	920593	611
-128973.336	379823	905
-128973.336	824956	607
-128973.336	645052	910
-128973.336	742688	594

THE BEST LOGLIKELIHOOD VALUE HAS BEEN REPLICATED. RERUN WITH AT LEAST TWICE THE RANDOM STARTS TO CHECK THAT THE BEST LOGLIKELIHOOD IS STILL OBTAINED AND REPLICATED.

THE MODEL ESTIMATION TERMINATED NORMALLY

MODEL FIT INFORMATION

Number of Free Parameters 18

Loglikelihood

H0 Value -128973.336
H0 Scaling Correction Factor 1.3080
for MLR

Information Criteria

Akaike (AIC) 257982.672
Bayesian (BIC) 258130.863
Sample-Size Adjusted BIC 258073.660
(n* = (n + 2) / 24)

FINAL CLASS COUNTS AND PROPORTIONS FOR THE LATENT CLASSES
BASED ON THE ESTIMATED MODEL

Latent Classes		
1	3094.62154	0.11131
2	6716.69965	0.24159
3	17990.67881	0.64710

Scripts & Outputs

“Insomnia symptoms in children and adolescents: Screening for sleep problems with the Two-item Sleep Condition Indicator (SCI-02)”

```
FINAL CLASS COUNTS AND PROPORTIONS FOR THE
LATENT CLASSES
BASED ON ESTIMATED POSTERIOR PROBABILITIES

    Latent
   Classes

     1      3094.62154        0.11131
     2      6716.69965        0.24159
     3     17990.67881        0.64710


FINAL CLASS COUNTS AND PROPORTIONS FOR THE
LATENT CLASSES
BASED ON THEIR MOST LIKELY LATENT CLASS
MEMBERSHIP

Class Counts and Proportions

    Latent
   Classes

     1           3065        0.11024
     2           6569        0.23628
     3          18168        0.65348


CLASSIFICATION QUALITY

     Entropy                     0.863


Average Latent Class Probabilities for Most Likely Latent
Class Membership (Row)
by Latent Class (Column)

           1        2        3

    1   0.921    0.079    0.000
    2   0.041    0.880    0.078
    3   0.000    0.038    0.962


Classification Probabilities for the Most Likely Latent Class
Membership (Column)
by Latent Class (Row)

           1        2        3

    1   0.912    0.088    0.000
    2   0.036    0.861    0.103
    3   0.000    0.029    0.971


Logits for the Classification Probabilities for the Most Likely
Latent Class Membership (Column)
by Latent Class (Row)

              1        2        3

    1     13.724   11.383    0.000
    2     -1.051    2.123    0.000
    3    -13.786   -3.525    0.000


MODEL RESULTS
                                                     Two-Tailed
                    Estimate       S.E.  Est./S.E.    P-Value

Latent Class 1

 Means
    SLPNSSNC           1.108      0.014     79.573      0.000
    WORRYNC           -2.040      0.017   -118.142      0.000
    SOLC               1.488      0.034     43.966      0.000
    SLPDURC            1.192      0.024     49.384      0.000

 Variances
    SLPNSSNC           0.465      0.003    155.639      0.000
    WORRYNC            0.184      0.003     68.676      0.000
    SOLC               0.597      0.008     76.749      0.000
    SLPDURC            0.677      0.007     95.747      0.000

Latent Class 2

 Means
    SLPNSSNC           0.982      0.010     98.325      0.000
    WORRYNC           -0.687      0.022    -31.840      0.000
    SOLC               0.444      0.015     30.251      0.000
    SLPDURC            0.517      0.015     34.921      0.000

 Variances
    SLPNSSNC           0.465      0.003    155.639      0.000
    WORRYNC            0.184      0.003     68.676      0.000
    SOLC               0.597      0.008     76.749      0.000
    SLPDURC            0.677      0.007     95.747      0.000

Latent Class 3

 Means
    SLPNSSNC           0.885      0.005    167.165      0.000
    WORRYNC            0.607      0.005    127.737      0.000
    SOLC              -0.416      0.007    -61.368      0.000
    SLPDURC           -0.395      0.008    -51.870      0.000

 Variances
    SLPNSSNC           0.465      0.003    155.639      0.000
    WORRYNC            0.184      0.003     68.676      0.000
    SOLC               0.597      0.008     76.749      0.000
    SLPDURC            0.677      0.007     95.747      0.000

Categorical Latent Variables

 Means
    C#1               -1.760      0.034    -51.148      0.000
    C#2               -0.985      0.019    -50.760      0.000


QUALITY OF NUMERICAL RESULTS

     Condition Number for the Information Matrix
0.514E-02
       (ratio of smallest to largest eigenvalue)


TECHNICAL 11 OUTPUT

     Random Starts Specifications for the k-1 Class Analysis
Model
        Number of initial stage random starts                1000
        Number of final stage optimizations                   250


     VUONG-LO-MENDELL-RUBIN LIKELIHOOD RATIO
TEST FOR 2 (H0) VERSUS 3 CLASSES
```

```
          H0 Loglikelihood Value                  -131941.399
          2 Times the Loglikelihood Difference          5936.126
          Difference in the Number of Parameters              5
          Mean                                   14.458
          Standard Deviation                           30.006
          P-Value                                 0.0000

     LO-MENDELL-RUBIN ADJUSTED LRT TEST

          Value                                5822.329
          P-Value                                 0.0000


TECHNICAL 14 OUTPUT

     Random Starts Specifications for the k-1 Class Analysis
Model
        Number of initial stage random starts              1000
        Number of final stage optimizations                 250

     Random Starts Specification for the k-1 Class Model for
Generated Data
        Number of initial stage random starts                 2
        Number of final stage optimizations                   1
     Random Starts Specification for the k Class Model for
Generated Data
        Number of initial stage random starts                50
        Number of final stage optimizations                  10
     Number of bootstrap draws requested                    250


     PARAMETRIC BOOTSTRAPPED LIKELIHOOD RATIO
TEST FOR 2 (H0) VERSUS 3 CLASSES

          H0 Loglikelihood Value                  -131941.399
          2 Times the Loglikelihood Difference          5936.126
          Difference in the Number of Parameters              5
          Approximate P-Value                          0.0000
          Successful Bootstrap Draws                      250

     WARNING:  OF THE 250 BOOTSTRAP DRAWS, 152
DRAWS HAD BOTH A SMALLER LRT VALUE THAN THE
     OBSERVED LRT VALUE AND NOT A REPLICATED
BEST LOGLIKELIHOOD VALUE FOR THE 3-CLASS
MODEL.
     THIS MEANS THAT THE P-VALUE MAY NOT BE
TRUSTWORTHY DUE TO LOCAL MAXIMA.
     INCREASE THE NUMBER OF RANDOM STARTS
USING THE LRTSTARTS OPTION.


PLOT INFORMATION

The following plots are available:

  Histograms (sample values)
  Scatterplots (sample values)
  Sample means
  Estimated means, medians, modes, and percentiles
  Sample and estimated means
  Observed individual values
  Estimated means and observed individual values
  Estimated overall and class-specific distributions

SAVEDATA INFORMATION

  Save file
    Step1_LPA3M1.dat

  Order and format of variables

    SLPNSSNC       F10.3
    WORRYNC        F10.3
    SOLC           F10.3
    SLPDURC        F10.3
    CPROB1         F10.3
    CPROB2         F10.3
    CPROB3         F10.3
    C              F10.3
    ID             I6

  Save file format
    8F10.3 I6

  Save file record length    10000

  Save missing symbol        *


DIAGRAM INFORMATION

  Mplus diagrams are currently not available for Mixture
analysis.
  No diagram output was produced.

     Beginning Time:  11:26:26
        Ending Time:  12:34:43
       Elapsed Time:  01:08:17

MUTHEN & MUTHEN
3463 Stoner Ave.
Los Angeles, CA  90066

Tel: (310) 391-9971
Fax: (310) 391-8971
Web: www.StatModel.com
Support: Support@StatModel.com

Copyright (c) 1998-2021 Muthen & Muthen
```

Parametrization Model 2 - Varying variances and zero covariance

```
Mplus VERSION 8.7
MUTHEN & MUTHEN
07/11/2023   1:23 PM

INPUT INSTRUCTIONS

  TITLE: LPA 3 profile syntax: M2 Unconstrained variance, 0
covariance
  DATA:
  FILE IS mplus2.csv;
  ! Specifies file location for data file. Make sure data is in
format appropriate for Mplus
  ! per Mplus manual. This data file is in individual format
(one row of data per participan
  VARIABLE:
  NAMES ARE ID SCI_02 SCI_02B slpnssN worryN sol
slpDur gender genderN yrgrp yrgrpN SCI_02C
  slpnssNC worryNC solC slpDurC;
```

```
  ! All variables included in data file should be named here.
  USEVARIABLES ARE slpnssNC worryNC solC slpDurC;
  ! Only variables intended for use in the analysis should be
listed here
  IDVARIABLE IS ID;
  CLASSES = c (3);
  ! This is where you instruct Mplus on how many
classes/profiles are being estimated. Initi
  ! model contains only one class/profile, thus it would be
CLASSES = c (1). Above specifies
  !profiles, and for each further iterative models the number
in parentheses increases by on
  !three profiles/classes would be c (3), and so on.
  MISSING ARE ALL(9999);
  ! Used to communicate how missing data is coded in data
file. Here shown with a "." which
  ! all that is included in each cell with missing data in the
data file
  ANALYSIS:
  TYPE = MIXTURE;
  ! LPA is a version of mixture modeling, and this instructs
Mplus to analyze in this way
  ESTIMATOR = MLR;
  !FIML robust to non-normal data
  STARTS = 1000 250;
  STITERATIONS = 500;
  ! Default number of starts for each step of the ML
estimation. First STARTS value specifie
  !number of unique start values to start with, the 250
represents the 250 best unique start
  !carrying forward to completion. The STITERATIONS
specifies the number of ML iteration
  !steps for those 250 selected start values to go through to
be able to converge. This is a
  !maximum number of iteration; if a model converges in less
than 500 iterations it will sto
  !before reaching 500 iterations.
  !These values can be increased … see "Four-Profile Final
Model with Covariate Analysis
  !Syntax" for an example.
  LRTSTARTS = 2 1 50 10;
  LRTBOOTSTRAP = 250;
  !The above start values are for the defaults for the LRT
statistic being run to compare th
  !model fit with the model fit of a model with one less class
(k-1). The BOOTSTRAP statemen
  !specifies the number of bootstrap draws to inform Mplus'
bootstrapped LRT results.
  MODEL:
  !For a default Mplus model the LPA model does not need
to be specified. However, it can be
  !The model can also be modified from the Mplus default of
estimating the indicator means
  !(uniquely across profiles) and variances (constrained
across profiles), as well as the la
  !profile mean.
  %OVERALL%
  [slpnssNC worryNC solC slpDurC]; !estimates the
indicators means for each profile. Without
  !the means are freely estimated in each profile, not
constrained.
  slpnssNC worryNC solC slpDurC; !Label Var1-Var5
constrains the estimates of the variances
  !profiles to be equal.

  %c#1%

      [slpnssNC worryNC solC slpDurC];

      slpnssNC worryNC solC slpDurC;

    %c#2%

      [slpnssNC worryNC solC slpDurC];

      slpnssNC worryNC solC slpDurC;

    %c#3%

      [slpnssNC worryNC solC slpDurC];

      slpnssNC worryNC solC slpDurC;

  OUTPUT:
  TECH11 TECH14;
  ! TECH1 provides parameter specifications and starting
values for the analysis
  ! TECH8 provides optimization history for this analysis type
  !TECH11 provides LRT results
  !TECH14 provides bootstrapped LRT test
  PLOT: SERIES=slpnssNC worryNC solC slpDurC(*);
  TYPE=PLOT3;
  SAVEDATA:
  FILE IS Step1_LPA3M2.dat;
  ! Tells Mplus where to save the output files from the
analysis
  SAVE = CPROBABILITIES;
  ! The above command lines are to save the most likely
profile membership for each particip
  ! and the posterior probabilities for their membership in
each latent profile.

*** WARNING in MODEL command
  All variables are uncorrelated with all other variables within
class.
  Check that this is what is intended.


LPA 3 profile syntax: m2 Unconstrained variance, 0
covariance

SUMMARY OF ANALYSIS

Number of groups                                   1
Number of observations                             27802

Number of dependent variables                      4
Number of independent variables                    0
Number of continuous latent variables              0
Number of categorical latent variables             1

Observed dependent variables

  Continuous
   SLPNSSNC    WORRYNC     SOLC       SLPDURC

Categorical latent variables
   C

Variables with special functions

  ID variable          ID

Estimator                                      MLR
Information matrix                             OBSERVED
Optimization Specifications for the Quasi-Newton Algorithm
for
Continuous Outcomes
  Maximum number of iterations                     100
```

```
  Convergence criterion                           0.100D-05
Optimization Specifications for the EM Algorithm
  Maximum number of iterations                          500
  Convergence criteria
    Loglikelihood change                          0.100D-06
    Relative loglikelihood change                 0.100D-06
    Derivative                                0.100D-05
Optimization Specifications for the M step of the EM
Algorithm for
Categorical Latent variables
  Number of M step iterations                         1
  M step convergence criterion                    0.100D-05
  Basis for M step termination                    ITERATION
Optimization Specifications for the M step of the EM
Algorithm for
Censored, Binary or Ordered Categorical (Ordinal),
Unordered
Categorical (Nominal) and Count Outcomes
  Number of M step iterations                         1
  M step convergence criterion                    0.100D-05
  Basis for M step termination                    ITERATION
  Maximum value for logit thresholds                   15
  Minimum value for logit thresholds                  -15
  Minimum expected cell size for chi-square          0.100D-
01
Maximum number of iterations for H1                  2000
Convergence criterion for H1                      0.100D-03
Optimization algorithm                              EMA
Random Starts Specifications
  Number of initial stage random starts              1000
  Number of final stage optimizations                 250
  Number of initial stage iterations                 500
  Initial stage convergence criterion             0.100D+01
  Random starts scale                           0.500D+01
  Random seed for generating random starts                0

Input data file(s)
  mplus2.csv
Input data format  FREE


SUMMARY OF DATA

     Number of missing data patterns           4
     Number of y missing data patterns         4
     Number of u missing data patterns         0


COVARIANCE COVERAGE OF DATA

Minimum covariance coverage value   0.100


     PROPORTION OF DATA PRESENT FOR Y


           Covariance Coverage
              SLPNSSNC      WORRYNC       SOLC
SLPDURC
              ________      ________      ________      ________
 SLPNSSNC       1.000
 WORRYNC        1.000         1.000
 SOLC           0.976         0.976         0.976
 SLPDURC        0.963         0.963         0.940         0.963


UNIVARIATE SAMPLE STATISTICS

     UNIVARIATE HIGHER-ORDER MOMENT
DESCRIPTIVE STATISTICS

         Variable/         Mean/      Skewness/   Minimum/ % with
Percentiles
        Sample Size      Variance    Kurtosis    Maximum
Min/Max      20%/60%    40%/80%    Median

     SLPNSSNC           0.933       0.086       0.000   27.10%
0.000      1.000      1.000
          27802.000       0.471      -0.878       2.000   20.40%
1.000      2.000
     WORRYNC            0.000      -1.036      -2.825    1.71%
-0.928      0.020      0.495
          27802.000       1.000       0.165       0.969   28.55%
0.495      0.969
     SOLC               0.000       0.967      -1.054   31.63%     -
1.054     -0.072     -0.072
          27123.000       1.000       0.467       2.875    2.77%
-0.072      0.910
     SLPDURC            0.000       0.568      -1.205   25.70%
-1.205     -0.292     -0.292
          26763.000       1.000      -0.470       2.445    3.44%
-0.292      0.620

RANDOM STARTS RESULTS RANKED FROM THE BEST
TO THE WORST LOGLIKELIHOOD VALUES

Unperturbed starting value run did not converge in the initial
stage
optimizations.

999 perturbed starting value run(s) did not converge in the
initial stage
optimizations.

Final stage loglikelihood values at local maxima, seeds, and
initial stage start numbers:


250 perturbed starting value run(s) did not converge or were
rejected in the third stage.



     THE ESTIMATED COVARIANCE MATRIX FOR THE Y
VARIABLES IN CLASS 1 COULD NOT
     BE INVERTED.  PROBLEM INVOLVING VARIABLE
SLPDURC.  COMPUTATION COULD
     NOT BE COMPLETED IN ITERATION 17.  CHANGE
YOUR MODEL AND/OR STARTING
     VALUES.  THIS MAY BE DUE TO A ZERO ESTIMATED
VARIANCE, THAT IS, NO WITHIN-CLASS
     VARIATION FOR THE VARIABLE.
     THE MODEL ESTIMATION DID NOT TERMINATE
NORMALLY.  ESTIMATES CANNOT
     BE TRUSTED.



     THE MODEL ESTIMATION DID NOT TERMINATE
NORMALLY DUE TO AN ERROR IN THE
     COMPUTATION.  CHANGE YOUR MODEL AND/OR
STARTING VALUES.

MODEL RESULTS

                   Estimate

Latent Class 1
```

```
 Means
    SLPNSSNC          -0.197
    WORRYNC           55.382
    SOLC           -1907.460
    SLPDURC         -266.045

 Variances
    SLPNSSNC           6.868
    WORRYNC           37.105
    SOLC            3564.902
    SLPDURC            0.000

Latent Class 2

 Means
    SLPNSSNC           0.508
    WORRYNC           -0.016
    SOLC              -0.134
    SLPDURC            1.084

 Variances
    SLPNSSNC           0.302
    WORRYNC            0.399
    SOLC               0.078
    SLPDURC            0.598

Latent Class 3

 Means
    SLPNSSNC           0.941
    WORRYNC            0.000
    SOLC               0.003
    SLPDURC           -0.020

 Variances
    SLPNSSNC           0.470
    WORRYNC            1.011
    SOLC               1.015
    SLPDURC            0.986

Categorical Latent Variables

 Means
    C#1             -233.972
    C#2               -3.905


MODEL COMMAND WITH FINAL ESTIMATES USED AS
STARTING VALUES

     %OVERALL%

     [ c#1*-233.97177 ];
     [ c#2*-3.90489 ];

     %C#1%

     [ slpnssnc*-0.19736 ];
     [ worrync*55.38240 ];
     [ solc*-1907.45996 ];
     [ slpdurc*-266.04523 ];

     slpnssnc*6.86776;
     worrync*37.10458;
     solc*3564.90186;
     slpdurc*0.00000;

     %C#2%

     [ slpnssnc*0.50800 ];
     [ worrync*-0.01614 ];
     [ solc*-0.13390 ];
     [ slpdurc*1.08362 ];

     slpnssnc*0.30176;
     worrync*0.39880;
     solc*0.07827;
     slpdurc*0.59777;

     %C#3%

     [ slpnssnc*0.94078 ];
     [ worrync*0.00030 ];
     [ solc*0.00289 ];
     [ slpdurc*-0.01953 ];

     slpnssnc*0.47027;
     worrync*1.01098;
     solc*1.01502;
     slpdurc*0.98561;



TECHNICAL 11 OUTPUT

     Random Starts Specifications for the k-1 Class Analysis
Model
        Number of initial stage random starts              1000
        Number of final stage optimizations                 250



TECHNICAL 14 OUTPUT

     Random Starts Specifications for the k-1 Class Analysis
Model
        Number of initial stage random starts              1000
        Number of final stage optimizations                 250

     Random Starts Specification for the k-1 Class Model for
Generated Data
        Number of initial stage random starts                 2
        Number of final stage optimizations                   1
     Random Starts Specification for the k Class Model for
Generated Data
        Number of initial stage random starts                50
        Number of final stage optimizations                  10
     Number of bootstrap draws requested                    250


SAVEDATA INFORMATION

     Class probabilities were not computed.
     No data were saved.


DIAGRAM INFORMATION

  Mplus diagrams are currently not available for Mixture
analysis.
  No diagram output was produced.


     Beginning Time:  13:23:15
        Ending Time:  13:26:44
       Elapsed Time:  00:03:29

MUTHEN & MUTHEN
3463 Stoner Ave.
Los Angeles, CA  90066
```

Tel: (310) 391-9971
Fax: (310) 391-8971
Web: www.StatModel.com
Support: Support@StatModel.com

Copyright (c) 1998-2021 Muthen & Muthen

Parametrization Model 3 - Equal variances and equal covariances

```
Mplus VERSION 8.7
MUTHEN & MUTHEN
07/11/2023   1:29 PM

INPUT INSTRUCTIONS

  TITLE: LPA 3 profile syntax: M3 constrained variance and equal covariance
  DATA:
  FILE IS mplus2.csv;
  ! Specifies file location for data file. Make sure data is in format appropriate for Mplus
  ! per Mplus manual. This data file is in individual format (one row of data per participan
  VARIABLE:
  NAMES ARE ID SCI_02 SCI_02B slpnssN worryN sol slpDur gender genderN yrgrp yrgrpN SCI_02C
  slpnssNC worryNC solC slpDurC;

  ! All variables included in data file should be named here.
  USEVARIABLES ARE slpnssNC worryNC solC slpDurC;
  ! Only variables intended for use in the analysis should be listed here
  IDVARIABLE IS ID;
  CLASSES = c (3);
  ! This is where you instruct Mplus on how many classes/profiles are being estimated. Initi
  ! model contains only one class/profile, thus it would be CLASSES = c (1). Above specifies
  !profiles, and for each further iterative models the number in parentheses increases by on
  !three profiles/classes would be c (3), and so on.
  MISSING ARE ALL(9999);
  ! Used to communicate how missing data is coded in data file. Here shown with a "." which
  ! all that is included in each cell with missing data in the data file
  ANALYSIS:
  TYPE = MIXTURE;
  ! LPA is a version of mixture modeling, and this instructs Mplus to analyze in this way
  ESTIMATOR = MLR;
  !FIML robust to non-normal data
  STARTS = 1000 250;
  STITERATIONS = 500;
  ! Default number of starts for each step of the ML estimation. First STARTS value specifie
  !number of unique start values to start with, the 250 represents the 250 best unique start
  !carrying forward to completion. The STITERATIONS specifies the number of ML iteration
  !steps for those 250 selected start values to go through to be able to converge. This is a
  !maximum number of iteration; if a model converges in less than 500 iterations it will sto
  !before reaching 500 iterations.
  !These values can be increased … see "Four-Profile Final Model with Covariate Analysis
  !Syntax" for an example.
  LRTSTARTS = 2 1 50 10;
  LRTBOOTSTRAP = 250;
  !The above start values are for the defaults for the LRT statistic being run to compare th
  !model fit with the model fit of a model with one less class (k-1). The BOOTSTRAP statemen
  !specifies the number of bootstrap draws to inform Mplus' bootstrapped LRT results.
  MODEL:
  !For a default Mplus model the LPA model does not need to be specified. However, it can be
  !The model can also be modified from the Mplus default of estimating the indicator means
  !(uniquely across profiles) and variances (constrained across profiles), as well as the la
  !profile mean.
  %OVERALL%
  [slpnssNC worryNC solC slpDurC]; !estimates the indicators means for each profile. Without
  !the means are freely estimated in each profile, not constrained.
  slpnssNC worryNC solC slpDurC; !Label Var1-Var5 constrains the estimates of the variances
  !profiles to be equal.

  !covariances
  slpnssNC WITH worryNC solC slpDurC;
  worryNC WITH solC slpDurC;
  solC WITH slpDurC;

  OUTPUT:
  TECH11 TECH14;
  ! TECH1 provides parameter specifications and starting values for the analysis
  ! TECH8 provides optimization history for this analysis type
  !TECH11 provides LRT results
  !TECH14 provides bootstrapped LRT test
  PLOT: SERIES=slpnssNC worryNC solC slpDurC(*);
  TYPE=PLOT3;
  SAVEDATA:
  FILE IS Step1_LPA3M3.dat;
  ! Tells Mplus where to save the output files from the analysis
  SAVE = CPROBABILITIES;
  ! The above command lines are to save the most likely profile membership for each particip
  ! and the posterior probabilities for their membership in each latent profile.

LPA 3 profile syntax: M3 constrained variance and equal covariance

SUMMARY OF ANALYSIS

Number of groups                                1
Number of observations                      27802

Number of dependent variables                   4
Number of independent variables                 0
Number of continuous latent variables           0
Number of categorical latent variables          1

Observed dependent variables
```

```
  Continuous
   SLPNSSNC    WORRYNC     SOLC       SLPDURC

Categorical latent variables
  C

Variables with special functions

  ID variable         ID

Estimator                                          MLR
Information matrix                                 OBSERVED
Optimization Specifications for the Quasi-Newton Algorithm
for
Continuous Outcomes
  Maximum number of iterations                      100
  Convergence criterion                         0.100D-05
Optimization Specifications for the EM Algorithm
  Maximum number of iterations                      500
  Convergence criteria
    Loglikelihood change                        0.100D-06
    Relative loglikelihood change               0.100D-06
    Derivative                                  0.100D-05
Optimization Specifications for the M step of the EM
Algorithm for
Categorical Latent variables
  Number of M step iterations                         1
  M step convergence criterion                  0.100D-05
  Basis for M step termination                  ITERATION
Optimization Specifications for the M step of the EM
Algorithm for
Censored, Binary or Ordered Categorical (Ordinal),
Unordered
Categorical (Nominal) and Count Outcomes
  Number of M step iterations                         1
  M step convergence criterion                  0.100D-05
  Basis for M step termination                  ITERATION
  Maximum value for logit thresholds                 15
  Minimum value for logit thresholds                -15
  Minimum expected cell size for chi-square     0.100D-
01
Maximum number of iterations for H1                2000
Convergence criterion for H1                    0.100D-03
Optimization algorithm                              EMA
Random Starts Specifications
  Number of initial stage random starts            1000
  Number of final stage optimizations               250
  Number of initial stage iterations                500
  Initial stage convergence criterion           0.100D+01
  Random starts scale                           0.500D+01
  Random seed for generating random starts            0

Input data file(s)
  mplus2.csv
Input data format  FREE

SUMMARY OF DATA

     Number of missing data patterns            4
     Number of y missing data patterns          4
     Number of u missing data patterns          0

COVARIANCE COVERAGE OF DATA

Minimum covariance coverage value   0.100

     PROPORTION OF DATA PRESENT FOR Y

           Covariance Coverage
              SLPNSSNC     WORRYNC      SOLC
SLPDURC
              ________    ________    ________    ________
 SLPNSSNC      1.000
 WORRYNC       1.000       1.000
 SOLC          0.976       0.976       0.976
 SLPDURC       0.963       0.963       0.940       0.963

UNIVARIATE SAMPLE STATISTICS

     UNIVARIATE HIGHER-ORDER MOMENT
DESCRIPTIVE STATISTICS

         Variable/       Mean/     Skewness/   Minimum/ % with
Percentiles
        Sample Size      Variance    Kurtosis    Maximum
Min/Max      20%/60%    40%/80%    Median

     SLPNSSNC            0.933       0.086       0.000   27.10%
0.000      1.000      1.000
           27802.000       0.471      -0.878       2.000   20.40%
1.000      2.000
     WORRYNC             0.000      -1.036      -2.825    1.71%
-0.928      0.020      0.495
           27802.000       1.000       0.165       0.969   28.55%
0.495      0.969
     SOLC                0.000       0.967      -1.054   31.63%      -
1.054     -0.072     -0.072
           27123.000       1.000       0.467       2.875    2.77%
-0.072      0.910
     SLPDURC             0.000       0.568      -1.205   25.70%
-1.205     -0.292     -0.292
           26763.000       1.000      -0.470       2.445    3.44%
-0.292      0.620

RANDOM STARTS RESULTS RANKED FROM THE BEST
TO THE WORST LOGLIKELIHOOD VALUES

8 perturbed starting value run(s) did not converge in the
initial stage
optimizations.

Final stage loglikelihood values at local maxima, seeds, and
initial stage start numbers:

        -126772.383  349562           359
        -126772.383  869564           869
        -126772.383  347515           24
        -126772.383  848331           137
        -126772.383  588923           606
        -126772.383  781489           627
        -126772.383  440841           118
        -126772.383  77571            980
        -126772.383  407168           44
        -126772.383  351807           617
        -126772.383  63231            935
        -126772.383  631413           439
        -126772.383  415931           10
        -126772.383  294811           637
        -126772.383  354208           196
        -126772.383  319144           176
        -126772.383  535804           111
        -126772.383  57226            208
        -126772.383  782821           272
        -126772.383  967902           52
        -126772.383  173191           422
        -126772.383  444228           860
        -126772.383  251641           784
        -126772.383  669639           699
        -126772.383  370957           554
```

-126772.383	965994	396	-126772.383	424223	900
-126772.383	995648	936	-126772.383	66276	217
-126772.383	592219	119	-126772.383	939021	8
-126772.383	617658	660	-126772.383	939709	112
-126772.383	634782	979	-126772.383	244349	736
-126772.383	140442	500	-126772.383	804561	59
-126772.383	213189	605	-126772.383	501995	791
-126772.383	505244	582	-126772.383	569131	26
-126772.383	471398	74	-126772.383	391179	78
-126772.383	860102	495	-126772.383	529455	268
-126772.383	567165	319	-126772.383	366533	484
-126772.383	226322	478	-126772.383	404426	344
-126772.383	260601	36	-126772.383	520865	763
-126772.383	147440	514	-126772.383	266340	683
-126772.383	85114	385	-126772.383	836515	341
-126772.383	965639	463	-126772.383	622860	259
-126772.383	887676	22	-126772.383	748692	204
-126772.383	164305	128	-126772.383	462821	745
-126772.383	50887	389	-126772.383	202790	198
-126772.383	422103	62	-126772.383	638577	769
-126772.383	425149	878	-126772.383	344422	296
-126772.383	777492	972	-126772.383	118438	601
-126772.383	98068	998	-126772.383	79212	517
-126772.383	636396	168	-126772.383	539389	544
-126772.383	414828	322	-126772.383	328661	813
-126772.383	417035	149	-126772.383	488125	368
-126772.383	609089	241	-126772.383	714997	399
-126772.383	859432	770	-126772.383	227563	63
-126772.383	406935	958	-126772.383	686482	215
-126772.383	94610	884	-126772.383	213532	503
-126772.383	387701	275	-126772.383	441191	361
-126772.383	473343	844	-126772.383	973369	202
-126772.383	579995	183	-126772.383	105435	265
-126772.383	726744	939	-126772.383	434915	552
-126772.383	251680	794	-126772.383	712702	684
-126772.383	68850	462	-126772.383	431271	943
-126772.383	760850	739	-126772.383	284716	713
-126772.383	677062	680	-126772.383	136842	58
-126772.383	642386	662	-126772.383	65651	214
-126772.383	425982	485	-126772.383	850112	922
-126772.383	370466	41	-126772.383	130011	587
-126772.383	484687	306	-126772.383	40327	971
-126772.383	508482	446	-126772.383	12477	155
-126772.383	648555	113	-126772.383	926797	406
-126772.383	502157	799	-126772.383	783165	170
-126772.383	423661	875	-126772.383	455617	242
-126772.383	195353	225	-126772.383	610181	711
-126772.383	93468	3	-126772.383	271809	846
-126772.383	650371	14	-126772.383	193569	440
-126772.383	972873	157	-126772.383	858804	534
-126772.383	742688	594	-126772.383	402699	604
-126772.383	89970	223	-126772.383	203508	806
-126772.383	194143	510	-126772.383	4091	548
-126772.383	884242	957	-126772.383	942358	644
-126772.383	343926	624	-127270.473	638977	643
-126772.383	903633	553	-127270.473	210870	383
-126772.383	891347	504	-127270.473	476498	179
-126772.383	131856	90	-127270.473	584397	428
-126772.383	40340	188	-127270.473	789528	837
-126772.383	22089	143	-127270.473	945065	255
-126772.383	326091	759	-127270.473	224950	455
-126772.383	210139	991	-127270.473	476295	969
-126772.383	917702	693	-127270.473	608496	4
-126772.383	322790	636	-127270.473	739214	807
-126772.383	549244	756	-127270.473	534483	290
-126772.383	838615	677	-127270.473	354624	448
-126772.383	966499	963	-127270.473	902278	21
-126772.383	617243	237	-127270.473	137377	397
-126772.383	628143	854	-127270.473	691041	590
-126772.383	36714	201	-127270.473	625191	350

```
-127270.473  303834       798
-127270.473  963967       941
-127270.473  152496       123
-127270.473  392751       480
-127270.473  666720       671
-127270.473  967237       48
-127270.473  341519       653
-127270.473  188498       258
-127270.473  848590       694
-127270.473  985387       381
-127270.473  599729       658
-127270.473  582296       452
-127270.473  396614       918
-127270.473  618000       190
-127270.473  741888       138
-127270.473  59674        240
-127270.473  67009        564
-127270.473  879211       453
-127270.473  995249       525
-127270.473  70118        104
-127270.473  690596       858
-127270.473  539751       459
-127270.473  286735       175
-127270.473  49910        829
-127270.473  977800       853
-127270.473  216565       474
-127270.473  561664       392
-127270.473  214681       824
-127270.473  851945       18
-127270.473  872743       374
-127270.473  563584       657
-127270.473  807534       850
-127270.473  392418       28
-127270.473  238906       430
-127270.473  939870       655
-127270.473  126371       526
-127270.473  302046       863
-127270.473  999211       628
-127270.473  364676       27
-127270.473  137888       901
-127270.473  158612       581
-127270.473  345726       461
-127270.473  736574       414
-127270.473  544048       87
-127270.473  978061       488
-127270.473  849670       347
-127270.473  648035       836
-127270.473  783102       433
-127270.473  58507        793
-127270.473  73576        213
-127270.473  377504       294
-127270.473  436892       565
-127270.473  758647       951
-127270.473  347222       533
-127270.473  489927       520
-127270.473  unperturbed  0
-127270.473  529496       343
-127270.473  154575       539
-127270.473  732596       320
-127270.473  871438       561
-127270.473  685657       69
-127270.473  11984        934
-127270.473  3607         873
-127270.473  77210        712
-127270.473  863094       147
-127270.473  791678       974
-127270.473  283492       435
-127270.473  496710       386
-127270.473  265218       924
-127270.473  402224       91
-127270.473  845580       805
-127270.473  484406       421
-127270.473  643311       888
-127270.473  259507       53
-127270.473  688839       273
-127270.473  915642       40
-127270.473  207896       25
-127270.473  207917       976
-127270.473  964570       701
-127270.473  153053       378
-127270.473  311214       64
-127270.473  531546       702
-127270.473  220454       288
-127270.473  161421       519
-127270.473  248742       556

THE BEST LOGLIKELIHOOD VALUE HAS BEEN
REPLICATED.  RERUN WITH AT LEAST TWICE THE
RANDOM STARTS TO CHECK THAT THE BEST
LOGLIKELIHOOD IS STILL OBTAINED AND
REPLICATED.

THE MODEL ESTIMATION TERMINATED NORMALLY

MODEL FIT INFORMATION

Number of Free Parameters                  24

Loglikelihood

          H0 Value                   -126772.383
          H0 Scaling Correction Factor      1.2282
            for MLR

Information Criteria

          Akaike (AIC)                253592.765
          Bayesian (BIC)              253790.354
          Sample-Size Adjusted BIC    253714.083
            (n* = (n + 2) / 24)

FINAL CLASS COUNTS AND PROPORTIONS FOR THE
LATENT CLASSES
BASED ON THE ESTIMATED MODEL

    Latent
   Classes

       1       3218.02371          0.11575
       2      18879.70470          0.67908
       3       5704.27159          0.20517

FINAL CLASS COUNTS AND PROPORTIONS FOR THE
LATENT CLASSES
BASED ON ESTIMATED POSTERIOR PROBABILITIES

    Latent
   Classes

       1       3218.02371          0.11575
       2      18879.70470          0.67908
       3       5704.27159          0.20517

FINAL CLASS COUNTS AND PROPORTIONS FOR THE
LATENT CLASSES
BASED ON THEIR MOST LIKELY LATENT CLASS
MEMBERSHIP

Class Counts and Proportions
```

```
  Latent
 Classes

    1          3167         0.11391
    2         18928         0.68081
    3          5707         0.20527

CLASSIFICATION QUALITY

   Entropy                    0.870

Average Latent Class Probabilities for Most Likely Latent
Class Membership (Row)
by Latent Class (Column)

        1       2       3

  1   0.908   0.000   0.092
  2   0.000   0.975   0.025
  3   0.060   0.074   0.866


Classification Probabilities for the Most Likely Latent Class
Membership (Column)
by Latent Class (Row)

        1       2       3

  1   0.894   0.000   0.106
  2   0.000   0.978   0.022
  3   0.051   0.082   0.867


Logits for the Classification Probabilities for the Most Likely
Latent Class Membership (Column)
by Latent Class (Row)

          1        2        3

  1      2.131  -11.006    0.000
  2    -10.015    3.778    0.000
  3     -2.834   -2.352    0.000


MODEL RESULTS

                                          Two-Tailed
                  Estimate       S.E.  Est./S.E.    P-Value

Latent Class 1

 SLPNSSNC WITH
    WORRYNC           -0.018      0.003     -7.135      0.000
    SOLC               0.118      0.004     30.181      0.000
    SLPDURC            0.030      0.004      7.611      0.000

 WORRYNC  WITH
    SOLC              -0.155      0.005    -30.483      0.000
    SLPDURC           -0.129      0.004    -29.670      0.000

 SOLC     WITH
    SLPDURC            0.225      0.006     35.821      0.000

 Means
    SLPNSSNC           1.037      0.014     72.590      0.000
    WORRYNC           -2.034      0.019   -106.696      0.000
    SOLC               1.096      0.033     33.163      0.000
    SLPDURC            0.922      0.026     35.171      0.000

 Variances
    SLPNSSNC           0.469      0.003    156.716      0.000
    WORRYNC            0.168      0.002     89.473      0.000
    SOLC               0.754      0.009     88.524      0.000
    SLPDURC            0.790      0.007    107.265      0.000

Latent Class 2

 SLPNSSNC WITH
    WORRYNC           -0.018      0.003     -7.135      0.000
    SOLC               0.118      0.004     30.181      0.000
    SLPDURC            0.030      0.004      7.611      0.000

 WORRYNC  WITH
    SOLC              -0.155      0.005    -30.483      0.000
    SLPDURC           -0.129      0.004    -29.670      0.000

 SOLC     WITH
    SLPDURC            0.225      0.006     35.821      0.000

 Means
    SLPNSSNC           0.907      0.005    175.707      0.000
    WORRYNC            0.581      0.004    138.481      0.000
    SOLC              -0.314      0.006    -48.571      0.000
    SLPDURC           -0.301      0.007    -43.319      0.000

 Variances
    SLPNSSNC           0.469      0.003    156.716      0.000
    WORRYNC            0.168      0.002     89.473      0.000
    SOLC               0.754      0.009     88.524      0.000
    SLPDURC            0.790      0.007    107.265      0.000

Latent Class 3

 SLPNSSNC WITH
    WORRYNC           -0.018      0.003     -7.135      0.000
    SOLC               0.118      0.004     30.181      0.000
    SLPDURC            0.030      0.004      7.611      0.000

 WORRYNC  WITH
    SOLC              -0.155      0.005    -30.483      0.000
    SLPDURC           -0.129      0.004    -29.670      0.000

 SOLC     WITH
    SLPDURC            0.225      0.006     35.821      0.000

 Means
    SLPNSSNC           0.959      0.011     88.715      0.000
    WORRYNC           -0.777      0.017    -45.568      0.000
    SOLC               0.444      0.017     26.141      0.000
    SLPDURC            0.491      0.017     29.459      0.000

 Variances
    SLPNSSNC           0.469      0.003    156.716      0.000
    WORRYNC            0.168      0.002     89.473      0.000
    SOLC               0.754      0.009     88.524      0.000
    SLPDURC            0.790      0.007    107.265      0.000

Categorical Latent Variables

 Means
    C#1               -0.572      0.038    -15.206      0.000
    C#2                1.197      0.019     62.898      0.000

QUALITY OF NUMERICAL RESULTS

     Condition Number for the Information Matrix
0.123E-02
       (ratio of smallest to largest eigenvalue)

TECHNICAL 11 OUTPUT
```

```
     Random Starts Specifications for the k-1 Class Analysis
Model
        Number of initial stage random starts                1000
        Number of final stage optimizations                   250

     VUONG-LO-MENDELL-RUBIN LIKELIHOOD RATIO
TEST FOR 2 (H0) VERSUS 3 CLASSES

          H0 Loglikelihood Value                  -128233.817
          2 Times the Loglikelihood Difference           2922.868
          Difference in the Number of Parameters               5
          Mean                                  12.618
          Standard Deviation                           15.491
          P-Value                                0.0000

     LO-MENDELL-RUBIN ADJUSTED LRT TEST

          Value                                2866.836
          P-Value                                0.0000

TECHNICAL 14 OUTPUT

     Random Starts Specifications for the k-1 Class Analysis
Model
        Number of initial stage random starts                1000
        Number of final stage optimizations                   250

     Random Starts Specification for the k-1 Class Model for
Generated Data
        Number of initial stage random starts                   2
        Number of final stage optimizations                     1
     Random Starts Specification for the k Class Model for
Generated Data
        Number of initial stage random starts                  50
        Number of final stage optimizations                    10
     Number of bootstrap draws requested                      250

     PARAMETRIC BOOTSTRAPPED LIKELIHOOD RATIO
TEST FOR 2 (H0) VERSUS 3 CLASSES

          H0 Loglikelihood Value                  -128233.817
          2 Times the Loglikelihood Difference           2922.868
          Difference in the Number of Parameters               5
          Approximate P-Value                         0.0000
          Successful Bootstrap Draws                        250

     WARNING:  OF THE 250 BOOTSTRAP DRAWS, 174
DRAWS HAD BOTH A SMALLER LRT VALUE THAN THE
     OBSERVED LRT VALUE AND NOT A REPLICATED
BEST LOGLIKELIHOOD VALUE FOR THE 3-CLASS
MODEL.
     THIS MEANS THAT THE P-VALUE MAY NOT BE
TRUSTWORTHY DUE TO LOCAL MAXIMA.
     INCREASE THE NUMBER OF RANDOM STARTS
USING THE LRTSTARTS OPTION.

PLOT INFORMATION

The following plots are available:

  Histograms (sample values)
  Scatterplots (sample values)
  Sample means
  Estimated means, medians, modes, and percentiles
  Sample and estimated means
  Observed individual values
  Estimated means and observed individual values
  Estimated overall and class-specific distributions

SAVEDATA INFORMATION


  Save file
    Step1_LPA3M3.dat

  Order and format of variables

    SLPNSSNC       F10.3
    WORRYNC        F10.3
    SOLC           F10.3
    SLPDURC        F10.3
    CPROB1         F10.3
    CPROB2         F10.3
    CPROB3         F10.3
    C              F10.3
    ID             I6

  Save file format
    8F10.3 I6

  Save file record length    10000

  Save missing symbol        *


DIAGRAM INFORMATION

  Mplus diagrams are currently not available for Mixture
analysis.
  No diagram output was produced.

     Beginning Time:  13:29:05
        Ending Time:  14:43:06
       Elapsed Time:  01:14:01

MUTHEN & MUTHEN
3463 Stoner Ave.
Los Angeles, CA  90066

Tel: (310) 391-9971
Fax: (310) 391-8971
Web: www.StatModel.com
Support: Support@StatModel.com

Copyright (c) 1998-2021 Muthen & Muthen
```

Parametrization Model 4 - Varying variances and equal covariances

```
Mplus VERSION 8.7
MUTHEN & MUTHEN
07/11/2023   3:04 PM

INPUT INSTRUCTIONS

  TITLE: LPA 3 profile syntax: M4: Varying means, varying
variances, and equal covariances

  DATA:
  FILE IS mplus2.csv;
  ! Specifies file location for data file. Make sure data is in
format appropriate for Mplus
```

```
  ! per Mplus manual. This data file is in individual format
(one row of data per participan
  VARIABLE:
  NAMES ARE ID SCI_02 SCI_02B slpnssN worryN sol
slpDur gender genderN yrgrp yrgrpN SCI_02C
  slpnssNC worryNC solC slpDurC;

  ! All variables included in data file should be named here.
  USEVARIABLES ARE slpnssNC worryNC solC slpDurC;
  ! Only variables intended for use in the analysis should be
listed here
  IDVARIABLE IS ID;
  CLASSES = c (3);
  ! This is where you instruct Mplus on how many
classes/profiles are being estimated. Initi
  ! model contains only one class/profile, thus it would be
CLASSES = c (1). Above specifies
  !profiles, and for each further iterative models the number
in parentheses increases by on
  !three profiles/classes would be c (3), and so on.
  MISSING ARE ALL(9999);
  ! Used to communicate how missing data is coded in data
file. Here shown with a "." which
  ! all that is included in each cell with missing data in the
data file
  ANALYSIS:
  TYPE = MIXTURE;
  ! LPA is a version of mixture modeling, and this instructs
Mplus to analyze in this way
  ESTIMATOR = MLR;
  !FIML robust to non-normal data
  STARTS = 1000 250;
  STITERATIONS = 500;
  ! Default number of starts for each step of the ML
estimation. First STARTS value specifie
  !number of unique start values to start with, the 250
represents the 250 best unique start
  !carrying forward to completion. The STITERATIONS
specifies the number of ML iteration
  !steps for those 250 selected start values to go through to
be able to converge. This is a
  !maximum number of iteration; if a model converges in less
than 500 iterations it will sto
  !before reaching 500 iterations.
  !These values can be increased … see "Four-Profile Final
Model with Covariate Analysis
  !Syntax" for an example.
  LRTSTARTS = 2 1 50 10;
  LRTBOOTSTRAP = 250;
  !The above start values are for the defaults for the LRT
statistic being run to compare th
  !model fit with the model fit of a model with one less class
(k-1). The BOOTSTRAP statemen
  !specifies the number of bootstrap draws to inform Mplus'
bootstrapped LRT results.
  MODEL:
  !For a default Mplus model the LPA model does not need
to be specified. However, it can be
  !The model can also be modified from the Mplus default of
estimating the indicator means
  !(uniquely across profiles) and variances (constrained
across profiles), as well as the la
  !profile mean.
  %OVERALL%
  [slpnssNC worryNC solC slpDurC]; !estimates the
indicators means for each profile. Without
  !the means are freely estimated in each profile, not
constrained.
  slpnssNC worryNC solC slpDurC; !Label Var1-Var5
constrains the estimates of the variances
  !profiles to be equal.

  !covariances
  slpnssNC WITH worryNC solC slpDurC;
  worryNC WITH solC slpDurC;
  solC WITH slpDurC;

  %c#1%

    [slpnssNC worryNC solC slpDurC];

    slpnssNC worryNC solC slpDurC;

   %c#2%

    [slpnssNC worryNC solC slpDurC];

    slpnssNC worryNC solC slpDurC;

   %c#3%

    [slpnssNC worryNC solC slpDurC];

    slpnssNC worryNC solC slpDurC;

  OUTPUT:
  TECH11 TECH14;
  ! TECH1 provides parameter specifications and starting
values for the analysis
  ! TECH8 provides optimization history for this analysis type
  !TECH11 provides LRT results
  !TECH14 provides bootstrapped LRT test
  PLOT: SERIES=slpnssNC worryNC solC slpDurC(*);
  TYPE=PLOT3;
  SAVEDATA:
  FILE IS Step1_LPA3M4.dat;
  ! Tells Mplus where to save the output files from the
analysis
  SAVE = CPROBABILITIES;
  ! The above command lines are to save the most likely
profile membership for each particip
  ! and the posterior probabilities for their membership in
each latent profile.

LPA 3 profile syntax: M4: Varying means, varying variances,
and equal covariances

SUMMARY OF ANALYSIS

Number of groups                                  1
Number of observations                        27802

Number of dependent variables                     4
Number of independent variables                   0
Number of continuous latent variables             0
Number of categorical latent variables            1

Observed dependent variables

  Continuous
   SLPNSSNC    WORRYNC     SOLC        SLPDURC

Categorical latent variables
   C

Variables with special functions

  ID variable           ID

Estimator                                       MLR
```

```
Information matrix                                OBSERVED
Optimization Specifications for the Quasi-Newton Algorithm
for
Continuous Outcomes
  Maximum number of iterations                          100
  Convergence criterion                             0.100D-05
Optimization Specifications for the EM Algorithm
  Maximum number of iterations                          500
  Convergence criteria
    Loglikelihood change                            0.100D-06
    Relative loglikelihood change                   0.100D-06
    Derivative                                      0.100D-05
Optimization Specifications for the M step of the EM
Algorithm for
Categorical Latent variables
  Number of M step iterations                             1
  M step convergence criterion                      0.100D-05
  Basis for M step termination                      ITERATION
Optimization Specifications for the M step of the EM
Algorithm for
Censored, Binary or Ordered Categorical (Ordinal),
Unordered
Categorical (Nominal) and Count Outcomes
  Number of M step iterations                             1
  M step convergence criterion                      0.100D-05
  Basis for M step termination                      ITERATION
  Maximum value for logit thresholds                     15
  Minimum value for logit thresholds                    -15
  Minimum expected cell size for chi-square        0.100D-
01
Maximum number of iterations for H1                    2000
Convergence criterion for H1                      0.100D-03
Optimization algorithm                                  EMA
Random Starts Specifications
  Number of initial stage random starts                1000
  Number of final stage optimizations                   250
  Number of initial stage iterations                    500
  Initial stage convergence criterion             0.100D+01
  Random starts scale                             0.500D+01
  Random seed for generating random starts                0

Input data file(s)
  mplus2.csv
Input data format  FREE

SUMMARY OF DATA

     Number of missing data patterns           4
     Number of y missing data patterns         4
     Number of u missing data patterns         0

COVARIANCE COVERAGE OF DATA

Minimum covariance coverage value   0.100


     PROPORTION OF DATA PRESENT FOR Y

           Covariance Coverage
              SLPNSSNC      WORRYNC       SOLC
SLPDURC
              ________      ________      ________      ________
 SLPNSSNC       1.000
 WORRYNC        1.000         1.000
 SOLC           0.976         0.976         0.976
 SLPDURC        0.963         0.963         0.940         0.963

UNIVARIATE SAMPLE STATISTICS

     UNIVARIATE HIGHER-ORDER MOMENT
DESCRIPTIVE STATISTICS

         Variable/         Mean/      Skewness/    Minimum/ % with
Percentiles
        Sample Size      Variance    Kurtosis    Maximum
Min/Max      20%/60%     40%/80%     Median

     SLPNSSNC             0.933       0.086       0.000   27.10%
0.000      1.000      1.000
           27802.000       0.471      -0.878       2.000   20.40%
1.000      2.000
     WORRYNC              0.000      -1.036      -2.825    1.71%
-0.928      0.020      0.495
           27802.000       1.000       0.165       0.969   28.55%
0.495      0.969
     SOLC                 0.000       0.967      -1.054   31.63%      -
1.054     -0.072     -0.072
           27123.000       1.000       0.467       2.875    2.77%
-0.072      0.910
     SLPDURC              0.000       0.568      -1.205   25.70%
-1.205     -0.292     -0.292
           26763.000       1.000      -0.470       2.445    3.44%
-0.292      0.620


RANDOM STARTS RESULTS RANKED FROM THE BEST
TO THE WORST LOGLIKELIHOOD VALUES

Unperturbed starting value run did not converge in the initial
stage
optimizations.

657 perturbed starting value run(s) did not converge in the
initial stage
optimizations.

Final stage loglikelihood values at local maxima, seeds, and
initial stage start numbers:

         -122273.536  464179           106
         -122273.536  791396           243
         -122273.536  208797           931
         -122273.536  985387           381
         -122273.536  507218           613
         -122273.536  408713           450
         -122273.536  961454           665
         -122273.536  856536           144
         -122273.536  520865           763
         -122273.536  640833           434
         -122273.536  210139           991
         -122273.536  996231           310
         -122273.536  622173           992
         -122273.536  970689           266
         -122273.536  838615           677
         -122273.536  783165           170
         -122273.536  49293            707
         -122273.536  86698            893
         -122273.536  436892           565
         -122273.536  724519           333
         -122273.536  512836           289
         -122273.536  152496           123
         -122273.536  987560           985
         -122273.536  669639           699
         -122273.536  538872           949
         -122273.536  46437            153
         -122273.536  995913           787
         -122273.536  662718           460
         -122273.536  903369           134
         -122273.536  153053           378
         -122273.536  535303           923
```

-122273.536	972873	157
-122273.536	307843	664
-122273.536	609185	181
-122273.536	85114	385
-122273.536	85734	411
-122273.536	876943	650
-122273.536	610181	711
-122273.536	395754	388
-122273.536	634782	979
-122273.536	416463	467
-122273.536	59963	670
-122273.536	751153	110
-122273.536	534193	689
-122273.536	865906	641
-122273.536	726744	939
-122273.536	920593	611
-122273.536	263268	165
-122273.536	443917	60
-122273.536	529455	268
-122273.536	645664	39
-122273.536	749453	33
-122273.536	344422	296
-122273.536	871722	355
-122273.536	278692	342
-122273.536	939870	655
-122273.536	813779	92
-122273.536	840031	276
-122273.536	514326	325
-122273.536	383979	603
-122273.536	206099	363
-122273.536	347515	24
-122273.536	485635	876
-122273.536	215353	164
-122273.536	791285	416
-122273.536	81233	825
-122273.536	964570	701
-122273.536	301717	823
-122273.536	549244	756
-122273.536	777492	972
-122273.536	376411	473
-122273.536	863691	481
-122273.536	436806	883
-122273.536	22362	365
-122273.536	268217	83
-122273.536	930872	277
-122273.536	211281	292
-122273.536	772131	407
-122273.536	765586	709
-122273.536	134830	780
-122273.536	241299	912
-122273.536	313306	612
-122273.536	178181	753
-122273.536	917702	693
-122273.536	752769	253
-122273.536	436460	89
-122273.536	243346	930
-122273.536	670281	721
-122273.536	507154	387
-122273.536	148918	682
-122273.536	118421	139
-122273.536	999211	628
-122273.536	462953	7
-122273.536	700270	855
-122273.536	857799	315
-122273.536	784664	75
-122273.536	679832	302
-122273.536	891531	206
-122273.536	471398	74
-122273.536	167409	772
-122273.536	787985	482
-122273.536	499347	881
-122273.536	81951	907
-122273.536	168762	200
-122273.536	741484	441
-122273.536	130541	896
-122273.536	432513	803
-122273.536	954914	911
-122273.536	212934	568
-122273.536	421731	886
-122273.536	605161	409
-122273.536	505244	582
-122273.536	579138	706
-122273.536	966603	919
-122273.536	751054	117
-122273.536	364676	27
-122273.536	597614	284
-122273.536	484501	163
-122273.536	121425	511
-122273.536	323588	826
-122273.536	637345	19
-122273.536	978781	497
-122273.536	942358	644
-122273.536	327475	518
-122273.536	643311	888
-122273.536	425929	508
-122273.536	762461	425
-122273.536	366706	29
-122273.536	177175	851
-122273.536	3607	873
-122273.536	475419	987
-122273.536	782821	272
-122273.536	210870	383
-122273.536	561664	392
-122273.536	384199	882
-122273.536	319575	499
-122273.536	805768	879
-122273.536	50887	389
-122273.536	327140	678
-122273.536	137377	397
-122273.536	580181	691
-122273.536	569833	85
-122273.536	349263	263
-122273.536	636396	168
-122273.536	292884	103
-122273.536	789985	67
-122273.536	227563	63
-122273.536	489927	520
-122273.536	965994	396
-122273.536	241197	747
-122273.536	629320	222
-122273.536	473942	574
-122273.536	710154	831
-122273.536	214681	824
-122273.536	326091	759
-122273.536	741888	138
-122273.536	580539	786
-122273.536	387701	275
-122273.536	648555	113
-122273.536	569131	26
-122273.536	508482	446
-122273.536	833196	715
-122273.536	699810	571
-122273.536	804104	566
-122273.536	765392	382
-122273.536	140442	500
-122273.536	465160	862
-122273.536	238317	812
-122273.536	937588	293
-122273.536	22874	588
-122273.536	458181	189

```
        -122273.536  953843        651
        -122273.536  650371        14
        -122273.536  930323        267
        -122273.536  495490        990
        -122273.536  665121        417
        -122273.536  638977        643
        -122273.536  314034        513
        -122273.536  793035        187
        -122273.536  544077        809
        -122273.536  618000        190
        -122273.536  939021        8
        -122273.536  529496        343
        -122273.536  836515        341
        -122273.536  829540        324
        -122273.536  77571         980
        -122273.536  804561        59
        -122273.536  178475        231
        -122273.536  118438        601
        -122273.536  193042        316
        -122273.536  682718        733
        -122273.536  715255        523
        -122273.536  604861        800
        -122273.536  588923        606
        -122273.536  673496        218
        -122273.536  231400        738
        -122273.536  197223        332
        -122273.536  264521        929
        -122273.536  798839        312
        -122273.536  440395        917
        -122273.536  66276         217
        -122273.536  459221        652
        -122273.536  312754        562
        -122273.536  995249        525
        -122273.536  855760        593
        -122273.536  29591         633
        -122273.536  92564         583
        -122273.536  194143        510
        -122273.536  791678        974
        -122273.536  92091         649
        -122273.536  303834        798
        -122273.536  67009         564
        -122273.536  268896        124
        -122273.536  291149        536
        -122273.536  872743        374
        -122273.536  843555        952
        -122273.536  875667        367
        -122273.536  783110        72
        -122273.536  359578        458
        -122273.536  391179        78
        -122273.536  888905        444
        -122273.536  695155        150
        -122273.536  794236        127
        -122273.536  679615        942
        -122273.536  17896         592
        -122273.536  573367        986
        -122273.536  694303        282
        -122273.536  281462        285
        -122273.536  474357        789
        -122273.536  551639        55
        -122273.536  645052        910
        -122273.536  352277        42
        -122273.536  685268        596
        -122273.536  677720        681
        -122273.536  153394        429
        -122273.536  761302        744
        -122273.536  848331        137
        -122273.536  471040        403
        -122273.536  746978        410
        -122273.536  267983        228
        -122273.536  486622        522
        -122273.536  318177        748
        -122273.536  213189        605
        -122273.536  42523         730
        -122273.536  848969        173
        -122273.536  991329        182
        -122273.536  123985        248
        -122273.536  738393        619

2 perturbed starting value run(s) did not converge or were
rejected in the third stage.

THE BEST LOGLIKELIHOOD VALUE HAS BEEN
REPLICATED.  RERUN WITH AT LEAST TWICE THE
RANDOM STARTS TO CHECK THAT THE BEST
LOGLIKELIHOOD IS STILL OBTAINED AND
REPLICATED.

THE MODEL ESTIMATION TERMINATED NORMALLY

MODEL FIT INFORMATION

Number of Free Parameters                  32

Loglikelihood

          H0 Value                  -122273.536
          H0 Scaling Correction Factor      1.0161
            for MLR

Information Criteria

          Akaike (AIC)               244611.072
          Bayesian (BIC)              244874.523
          Sample-Size Adjusted BIC      244772.828
            (n* = (n + 2) / 24)

FINAL CLASS COUNTS AND PROPORTIONS FOR THE
LATENT CLASSES
BASED ON THE ESTIMATED MODEL

    Latent
   Classes

       1       9597.87522         0.34522
       2      10278.69205         0.36971
       3       7925.43273         0.28507

FINAL CLASS COUNTS AND PROPORTIONS FOR THE
LATENT CLASSES
BASED ON ESTIMATED POSTERIOR PROBABILITIES

    Latent
   Classes

       1       9597.87522         0.34522
       2      10278.69205         0.36971
       3       7925.43273         0.28507

FINAL CLASS COUNTS AND PROPORTIONS FOR THE
LATENT CLASSES
BASED ON THEIR MOST LIKELY LATENT CLASS
MEMBERSHIP

Class Counts and Proportions

    Latent
   Classes

       1           11537         0.41497
       2            8897         0.32001
```

```
    3          7368         0.26502


CLASSIFICATION QUALITY

     Entropy                         0.664

Average Latent Class Probabilities for Most Likely Latent
Class Membership (Row)
by Latent Class (Column)

          1        2        3

   1   0.799    0.198    0.003
   2   0.042    0.832    0.126
   3   0.000    0.081    0.919

Classification Probabilities for the Most Likely Latent Class
Membership (Column)
by Latent Class (Row)

          1        2        3

   1   0.961    0.039    0.000
   2   0.222    0.720    0.058
   3   0.005    0.141    0.854

Logits for the Classification Probabilities for the Most Likely
Latent Class Membership (Column)
by Latent Class (Row)

             1        2        3

   1     11.610    8.413    0.000
   2      1.341    2.519    0.000
   3     -5.235   -1.801    0.000

MODEL RESULTS

                                                  Two-Tailed
                    Estimate       S.E.  Est./S.E.    P-Value

Latent Class 1

 SLPNSSNC WITH
    WORRYNC           -0.010      0.002     -4.859      0.000
    SOLC               0.077      0.003     23.898      0.000
    SLPDURC            0.008      0.004      2.133      0.033

 WORRYNC  WITH
    SOLC              -0.048      0.002    -22.837      0.000
    SLPDURC           -0.021      0.002    -11.280      0.000

 SOLC     WITH
    SLPDURC            0.045      0.003     13.331      0.000

 Means
    SLPNSSNC           0.879      0.009     99.742      0.000
    WORRYNC            0.805      0.007    113.188      0.000
    SOLC              -0.651      0.013    -50.397      0.000
    SLPDURC           -0.723      0.014    -50.364      0.000

 Variances
    SLPNSSNC           0.451      0.005     83.217      0.000
    WORRYNC            0.052      0.001     40.914      0.000
    SOLC               0.259      0.006     45.747      0.000
    SLPDURC            0.253      0.007     36.404      0.000

Latent Class 2

 SLPNSSNC WITH
    WORRYNC           -0.010      0.002     -4.859      0.000
    SOLC               0.077      0.003     23.898      0.000
    SLPDURC            0.008      0.004      2.133      0.033

 WORRYNC  WITH
    SOLC              -0.048      0.002    -22.837      0.000
    SLPDURC           -0.021      0.002    -11.280      0.000

 SOLC     WITH
    SLPDURC            0.045      0.003     13.331      0.000

 Means
    SLPNSSNC           0.899      0.009     99.109      0.000
    WORRYNC            0.178      0.030      5.939      0.000
    SOLC              -0.160      0.014    -11.305      0.000
    SLPDURC            0.059      0.021      2.866      0.004

 Variances
    SLPNSSNC           0.469      0.005     94.190      0.000
    WORRYNC            0.273      0.017     15.609      0.000
    SOLC               0.373      0.008     44.113      0.000
    SLPDURC            0.684      0.017     39.807      0.000

Latent Class 3

 SLPNSSNC WITH
    WORRYNC           -0.010      0.002     -4.859      0.000
    SOLC               0.077      0.003     23.898      0.000
    SLPDURC            0.008      0.004      2.133      0.033

 WORRYNC  WITH
    SOLC              -0.048      0.002    -22.837      0.000
    SLPDURC           -0.021      0.002    -11.280      0.000

 SOLC     WITH
    SLPDURC            0.045      0.003     13.331      0.000

 Means
    SLPNSSNC           1.042      0.010     99.351      0.000
    WORRYNC           -1.206      0.023    -53.488      0.000
    SOLC               1.012      0.031     33.071      0.000
    SLPDURC            0.812      0.019     42.391      0.000

 Variances
    SLPNSSNC           0.479      0.006     86.226      0.000
    WORRYNC            0.803      0.017     48.499      0.000
    SOLC               1.125      0.015     73.837      0.000
    SLPDURC            1.016      0.015     68.881      0.000

Categorical Latent Variables

 Means
    C#1                0.191      0.057      3.332      0.001
    C#2                0.260      0.034      7.668      0.000

QUALITY OF NUMERICAL RESULTS

     Condition Number for the Information Matrix
0.933E-04
       (ratio of smallest to largest eigenvalue)

TECHNICAL 11 OUTPUT

     Random Starts Specifications for the k-1 Class Analysis
Model
        Number of initial stage random starts                  1000
        Number of final stage optimizations                     250
```

```
     VUONG-LO-MENDELL-RUBIN LIKELIHOOD RATIO TEST FOR 2 (H0) VERSUS 3 CLASSES

          H0 Loglikelihood Value                  -124198.073
          2 Times the Loglikelihood Difference       3849.074
          Difference in the Number of Parameters            9
          Mean                                         27.434
          Standard Deviation                           33.435
          P-Value                                      0.0000

     LO-MENDELL-RUBIN ADJUSTED LRT TEST

          Value                                      3807.728
          P-Value                                      0.0000

TECHNICAL 14 OUTPUT

     Random Starts Specifications for the k-1 Class Analysis Model
        Number of initial stage random starts                1000
        Number of final stage optimizations                   250

     Random Starts Specification for the k-1 Class Model for Generated Data
        Number of initial stage random starts                   2
        Number of final stage optimizations                     1
     Random Starts Specification for the k Class Model for Generated Data
        Number of initial stage random starts                  50
        Number of final stage optimizations                    10
     Number of bootstrap draws requested                      250

     PARAMETRIC BOOTSTRAPPED LIKELIHOOD RATIO TEST FOR 2 (H0) VERSUS 3 CLASSES

          H0 Loglikelihood Value                  -124198.073
          2 Times the Loglikelihood Difference       3849.074
          Difference in the Number of Parameters            9
          Approximate P-Value                          0.0000
          Successful Bootstrap Draws                      250

     WARNING:  OF THE 250 BOOTSTRAP DRAWS, 199 DRAWS HAD BOTH A SMALLER LRT VALUE THAN THE
     OBSERVED LRT VALUE AND NOT A REPLICATED BEST LOGLIKELIHOOD VALUE FOR THE 3-CLASS MODEL.
     THIS MEANS THAT THE P-VALUE MAY NOT BE TRUSTWORTHY DUE TO LOCAL MAXIMA.
     INCREASE THE NUMBER OF RANDOM STARTS USING THE LRTSTARTS OPTION.

PLOT INFORMATION

The following plots are available:

  Histograms (sample values)
  Scatterplots (sample values)
  Sample means
  Estimated means, medians, modes, and percentiles
  Sample and estimated means
  Observed individual values
  Estimated means and observed individual values
  Estimated overall and class-specific distributions

SAVEDATA INFORMATION

  Save file
    Step1_LPA3M4.dat

  Order and format of variables

    SLPNSSNC       F10.3
    WORRYNC        F10.3
    SOLC           F10.3
    SLPDURC        F10.3
    CPROB1         F10.3
    CPROB2         F10.3
    CPROB3         F10.3
    C              F10.3
    ID             I6

  Save file format
    8F10.3 I6

  Save file record length    10000

  Save missing symbol        *

DIAGRAM INFORMATION

  Mplus diagrams are currently not available for Mixture analysis.
  No diagram output was produced.

     Beginning Time:  15:04:04
        Ending Time:  16:37:00
       Elapsed Time:  01:32:56

MUTHEN & MUTHEN
3463 Stoner Ave.
Los Angeles, CA  90066

Tel: (310) 391-9971
Fax: (310) 391-8971
Web: www.StatModel.com
Support: Support@StatModel.com

Copyright (c) 1998-2021 Muthen & Muthen
```

Parametrization Model 5 - Equal variances and varying covariances

```
Mplus VERSION 8.7
MUTHEN & MUTHEN
07/12/2023  10:37 AM

INPUT INSTRUCTIONS

  TITLE: LPA 3 profile syntax: M5: Varying means, equal variances, and varying covariances

  DATA:
  FILE IS mplus2.csv;
  ! Specifies file location for data file. Make sure data is in format appropriate for Mplus
  ! per Mplus manual. This data file is in individual format (one row of data per participan
  VARIABLE:
  NAMES ARE ID SCI_02 SCI_02B slpnssN worryN sol slpDur gender genderN yrgrp yrgrpN SCI_02C
  slpnssNC worryNC solC slpDurC;

  ! All variables included in data file should be named here.
  USEVARIABLES ARE slpnssNC worryNC solC slpDurC;
```

```
  ! Only variables intended for use in the analysis should be
listed here
  IDVARIABLE IS ID;
  CLASSES = c (3);
  ! This is where you instruct Mplus on how many
classes/profiles are being estimated. Initi
  ! model contains only one class/profile, thus it would be
CLASSES = c (1). Above specifies
  !profiles, and for each further iterative models the number
in parentheses increases by on
  !three profiles/classes would be c (3), and so on.
  MISSING ARE ALL(9999);
  ! Used to communicate how missing data is coded in data
file. Here shown with a "." which
  ! all that is included in each cell with missing data in the
data file
  ANALYSIS:
  TYPE = MIXTURE;
  ! LPA is a version of mixture modeling, and this instructs
Mplus to analyze in this way
  ESTIMATOR = MLR;
  !FIML robust to non-normal data
  STARTS = 1000 250;
  STITERATIONS = 500;
  ! Default number of starts for each step of the ML
estimation. First STARTS value specifie
  !number of unique start values to start with, the 250
represents the 250 best unique start
  !carrying forward to completion. The STITERATIONS
specifies the number of ML iteration
  !steps for those 250 selected start values to go through to
be able to converge. This is a
  !maximum number of iteration; if a model converges in less
than 500 iterations it will sto
  !before reaching 500 iterations.
  !These values can be increased … see "Four-Profile Final
Model with Covariate Analysis
  !Syntax" for an example.
  LRTSTARTS = 2 1 50 10;
  LRTBOOTSTRAP = 250;
  !The above start values are for the defaults for the LRT
statistic being run to compare th
  !model fit with the model fit of a model with one less class
(k-1). The BOOTSTRAP statemen
  !specifies the number of bootstrap draws to inform Mplus'
bootstrapped LRT results.
  MODEL:
  !For a default Mplus model the LPA model does not need
to be specified. However, it can be
  !The model can also be modified from the Mplus default of
estimating the indicator means
  !(uniquely across profiles) and variances (constrained
across profiles), as well as the la
  !profile mean.
  %OVERALL%
  [slpnssNC worryNC solC slpDurC]; !estimates the
indicators means for each profile. Without
  !the means are freely estimated in each profile, not
constrained.
  slpnssNC worryNC solC slpDurC; !Label Var1-Var5
constrains the estimates of the variances
  !profiles to be equal.

  %c#1%

    [slpnssNC worryNC solC slpDurC];

    !covariances
  slpnssNC WITH worryNC solC slpDurC;
  worryNC WITH solC slpDurC;
  solC WITH slpDurC;

  %c#2%

    [slpnssNC worryNC solC slpDurC];

    !covariances
  slpnssNC WITH worryNC solC slpDurC;
  worryNC WITH solC slpDurC;
  solC WITH slpDurC;

  %c#3%

    [slpnssNC worryNC solC slpDurC];

    !covariances
  slpnssNC WITH worryNC solC slpDurC;
  worryNC WITH solC slpDurC;
  solC WITH slpDurC;

  OUTPUT:
  TECH11 TECH14;
  ! TECH1 provides parameter specifications and starting
values for the analysis
  ! TECH8 provides optimization history for this analysis type
  !TECH11 provides LRT results
  !TECH14 provides bootstrapped LRT test
  PLOT: SERIES=slpnssNC worryNC solC slpDurC(*);
  TYPE=PLOT3;
  SAVEDATA:
  FILE IS Step1_LPA3M5.dat;
  ! Tells Mplus where to save the output files from the
analysis
  SAVE = CPROBABILITIES;
  ! The above command lines are to save the most likely
profile membership for each particip
  ! and the posterior probabilities for their membership in
each latent profile.

*** WARNING in MODEL command
  All variables are uncorrelated with all other variables within
class.
  Check that this is what is intended.

LPA 3 profile syntax: M5: Varying means, equal variances,
and varying covariances

SUMMARY OF ANALYSIS

Number of groups                                   1
Number of observations                         27802

Number of dependent variables                      4
Number of independent variables                    0
Number of continuous latent variables              0
Number of categorical latent variables             1

Observed dependent variables

  Continuous
   SLPNSSNC    WORRYNC     SOLC       SLPDURC

Categorical latent variables
   C

Variables with special functions

  ID variable           ID

Estimator                                        MLR
```

```
Information matrix                                  OBSERVED
Optimization Specifications for the Quasi-Newton Algorithm
for
Continuous Outcomes
  Maximum number of iterations                            100
  Convergence criterion                              0.100D-05
Optimization Specifications for the EM Algorithm
  Maximum number of iterations                            500
  Convergence criteria
    Loglikelihood change                             0.100D-06
    Relative loglikelihood change                    0.100D-06
    Derivative                                       0.100D-05
Optimization Specifications for the M step of the EM
Algorithm for
Categorical Latent variables
  Number of M step iterations                               1
  M step convergence criterion                       0.100D-05
  Basis for M step termination                      ITERATION
Optimization Specifications for the M step of the EM
Algorithm for
Censored, Binary or Ordered Categorical (Ordinal),
Unordered
Categorical (Nominal) and Count Outcomes
  Number of M step iterations                               1
  M step convergence criterion                       0.100D-05
  Basis for M step termination                      ITERATION
  Maximum value for logit thresholds                       15
  Minimum value for logit thresholds                      -15
  Minimum expected cell size for chi-square          0.100D-
01
Maximum number of iterations for H1                      2000
Convergence criterion for H1                         0.100D-03
Optimization algorithm                                   EMA
Random Starts Specifications
  Number of initial stage random starts                  1000
  Number of final stage optimizations                     250
  Number of initial stage iterations                      500
  Initial stage convergence criterion               0.100D+01
  Random starts scale                               0.500D+01
  Random seed for generating random starts                  0

Input data file(s)
  mplus2.csv
Input data format  FREE

SUMMARY OF DATA

     Number of missing data patterns            4
     Number of y missing data patterns          4
     Number of u missing data patterns          0

COVARIANCE COVERAGE OF DATA

Minimum covariance coverage value   0.100

     PROPORTION OF DATA PRESENT FOR Y

           Covariance Coverage
              SLPNSSNC      WORRYNC       SOLC
SLPDURC
              ________      ________      ________      ________
 SLPNSSNC       1.000
 WORRYNC        1.000         1.000
 SOLC           0.976         0.976         0.976
 SLPDURC        0.963         0.963         0.940         0.963

UNIVARIATE SAMPLE STATISTICS

     UNIVARIATE HIGHER-ORDER MOMENT
DESCRIPTIVE STATISTICS

         Variable/         Mean/     Skewness/   Minimum/ % with
Percentiles
        Sample Size      Variance    Kurtosis    Maximum
Min/Max      20%/60%    40%/80%    Median

     SLPNSSNC            0.933       0.086       0.000   27.10%
0.000      1.000      1.000
           27802.000      0.471      -0.878       2.000   20.40%
1.000      2.000
     WORRYNC             0.000      -1.036      -2.825    1.71%
-0.928     0.020      0.495
           27802.000      1.000       0.165       0.969   28.55%
0.495      0.969
     SOLC                0.000       0.967      -1.054   31.63%     -
1.054     -0.072     -0.072
           27123.000      1.000       0.467       2.875    2.77%
-0.072     0.910
     SLPDURC             0.000       0.568      -1.205   25.70%
-1.205     -0.292     -0.292
           26763.000      1.000      -0.470       2.445    3.44%
-0.292     0.620

RANDOM STARTS RESULTS RANKED FROM THE BEST
TO THE WORST LOGLIKELIHOOD VALUES

380 perturbed starting value run(s) did not converge in the
initial stage
optimizations.

Final stage loglikelihood values at local maxima, seeds, and
initial stage start numbers:

         -125994.415  691234          250
         -125994.415  246794          801
         -125994.415  917702          693
         -125994.415  970689          266
         -125994.415  244349          736
         -125994.415  478421          311
         -125994.415  278661          674
         -125994.415  930872          277
         -125994.415  618000          190
         -125994.415  49910           829
         -125994.415  314034          513
         -125994.415  91231           727
         -125994.415  851945          18
         -125994.415  926797          406
         -125994.415  791285          416
         -125994.415  544077          809
         -125994.415  268896          124
         -125994.415  27071           15
         -125994.415  177175          851
         -125994.415  485256          371
         -125994.415  495490          990
         -125994.415  349263          263
         -125994.415  3607            873
         -125994.415  168762          200
         -125994.415  109357          765
         -125994.415  544009          842
         -125994.415  180889          920
         -125994.415  636396          168
         -125994.415  772131          407
         -125994.415  383979          603
         -125994.415  373702          669
         -125994.415  551340          766
         -125994.415  303834          798
         -125994.415  294811          637
         -125994.415  383902          673
         -125994.415  462228          298
         -125994.415  215353          164
```

-125994.415	200041	810
-125994.415	130541	896
-125994.415	616917	261
-125994.415	126371	526
-125994.415	849670	347
-125994.415	237332	661
-125994.415	114433	708
-125994.415	903633	553
-125994.415	202790	198
-125994.415	833196	715
-125994.415	137377	397
-125994.415	399508	415
-125994.415	862607	468
-125994.415	544048	87
-125994.415	414284	158
-125994.415	490123	995
-125994.415	937885	426
-125994.415	105435	265
-125994.415	46502	714
-125994.415	992389	77
-125994.415	308582	752
-125994.415	373815	618
-125994.415	856536	144
-125994.415	679448	937
-125994.415	505879	424
-125994.415	224950	455
-125994.415	260953	589
-125994.415	339073	841
-125994.415	575700	100
-125994.415	549244	756
-125994.415	391949	295
-125994.415	701525	239
-125994.415	576596	99
-125994.415	794236	127
-125994.415	484687	306
-125994.415	972430	491
-125994.415	971693	470
-125994.415	453915	975
-125994.415	710154	831
-125994.415	850112	922
-125994.415	443442	380
-125994.415	444228	860
-125994.415	179022	687
-125994.415	50887	389
-125994.415	70118	104
-125994.415	204959	695
-125994.415	570908	98
-125994.415	907810	795
-125994.415	705224	953
-125994.415	963967	941
-125994.415	923437	398
-125994.415	138695	783
-125994.415	124999	96
-125994.415	634782	979
-125994.415	563584	657
-125994.415	742609	531
-125994.415	836515	341
-125994.415	848356	602
-125994.415	298553	773
-125994.415	745972	521
-125994.415	965639	463
-125994.415	264081	186
-125994.415	213532	503
-125994.415	284384	600
-125994.415	673496	218
-125994.415	440368	797
-125994.415	628143	854
-125994.415	847088	750
-125994.415	954914	911
-125994.415	271809	846
-125994.415	679832	302
-125994.415	809240	543
-125994.415	172913	557
-125994.415	830392	35
-125994.415	97300	640
-125994.415	597614	284
-125994.415	723035	538
-125994.415	682718	733
-125994.415	81951	907
-125994.415	879338	309
-125994.415	966499	963
-125994.415	669634	335
-125994.415	863691	481
-125994.415	717754	180
-125994.415	654136	167
-125994.415	193569	440
-125994.415	298201	903
-125994.415	347515	24
-125994.415	545108	667
-125994.415	980970	894
-125994.415	695155	150
-125994.415	766903	505
-125994.415	922596	456
-125994.415	51375	148
-125994.415	158612	581
-125994.415	364676	27
-125994.415	566739	575
-125994.415	442072	625
-125994.415	322790	636
-125994.415	852283	616
-125994.415	677062	680
-125994.415	696830	668
-125994.415	792389	746
-125994.415	535303	923
-125994.415	17896	592
-125994.415	857122	889
-125994.415	735928	916
-125994.415	961454	665
-125994.415	881886	608
-125994.415	370481	742
-125994.415	195353	225
-125994.415	405371	569
-125994.415	415931	10
-125994.415	148918	682
-125994.415	119513	821
-125994.415	303634	169
-125994.415	465160	862
-125994.415	414828	322
-125994.415	333082	578
-125994.415	928624	981
-125994.415	548245	818
-125994.415	599729	658
-125994.415	207896	25
-125994.415	485483	498
-125994.415	640833	434
-125994.415	762461	425
-125994.415	136842	58
-125994.415	311214	64
-125994.415	72662	729
-125994.415	297518	166
-125994.415	61587	400
-125994.415	569833	85
-125994.415	662718	460
-125994.415	484501	163
-125994.415	432513	803
-125994.415	843555	952
-125994.415	unperturbed	0
-125994.415	626208	698
-125994.415	599136	811
-125994.415	721392	768

```
          -125994.415  153394        429
          -125994.415  178031        720
          -125994.415  728038        177
          -125994.415  484406        421
          -125994.415  224151        973
          -125994.415  589483        950
          -125994.415  782821        272
          -125994.415  352277        42
          -125994.415  238906        430
          -125994.415  848331        137
          -125994.415  855760        593
          -125994.415  891347        504
          -125994.415  468036        131
          -125994.415  437181        135
          -125994.415  552272        654
          -125994.415  520177        262
          -125994.415  268217        83
          -125994.415  580405        286
          -125994.415  87586        871
          -125994.415  638977        643
          -125994.415  55115        408
          -125994.415  248742        556
          -125994.415  838615        677
          -125994.415  751153        110
          -125994.415  384199        882
          -125994.415  178181        753
          -125994.415  22362        365
          -125994.415  792993        859
          -125994.415  778953        635
          -125994.415  286735        175
          -125994.415  617243        237
          -125994.415  605161        409
          -125994.415  846194        93
          -125994.415  357866        968
          -125994.415  726035        191
          -125994.415  483369        270
          -125994.415  399380        436
          -125994.415  434915        552
          -125994.415  782200        84
          -125994.415  538872        949
          -125994.415  871851        257
          -125994.415  814975        129
          -125994.415  157351        579
          -125994.415  81233        825
          -125994.415  526324        178
          -125994.415  366706        29
          -125994.415  211281        292
          -125994.415  848969        173
          -125994.415  937588        293
          -125994.415  788796        145
          -125994.415  574942        558
          -125994.415  35191        703
          -125994.415  195873        6
          -125994.415  118438        601
          -125994.415  576726        280
          -125994.415  345974        622
          -125994.415  467339        66
          -125994.415  566687        597
          -125994.415  407108        366
          -125994.415  791678        974
          -125994.415  608460        244
          -125994.415  860029        760
          -125994.415  14262        781
          -125994.415  50983        834
          -125994.415  592219        119
          -125994.415  891531        206
          -125994.415  265218        924
          -125994.415  370957        554
          -125994.415  264935        281
          -125994.415  127362        757
          -125994.415  621542        375
          -125994.415  326091        759
          -125994.415  691041        590

THE BEST LOGLIKELIHOOD VALUE HAS BEEN
REPLICATED.  RERUN WITH AT LEAST TWICE THE
RANDOM STARTS TO CHECK THAT THE BEST
LOGLIKELIHOOD IS STILL OBTAINED AND
REPLICATED.

THE MODEL ESTIMATION TERMINATED NORMALLY

MODEL FIT INFORMATION

Number of Free Parameters                   36

Loglikelihood

          H0 Value                  -125994.415
          H0 Scaling Correction Factor      1.3296
            for MLR

Information Criteria

          Akaike (AIC)               252060.830
          Bayesian (BIC)             252357.213
          Sample-Size Adjusted BIC      252242.806
            (n* = (n + 2) / 24)

FINAL CLASS COUNTS AND PROPORTIONS FOR THE
LATENT CLASSES
BASED ON THE ESTIMATED MODEL

    Latent
   Classes

       1       5737.65936          0.20638
       2      18891.89637          0.67952
       3       3172.44427          0.11411

FINAL CLASS COUNTS AND PROPORTIONS FOR THE
LATENT CLASSES
BASED ON ESTIMATED POSTERIOR PROBABILITIES

    Latent
   Classes

       1       5737.65936          0.20638
       2      18891.89637          0.67952
       3       3172.44427          0.11411

FINAL CLASS COUNTS AND PROPORTIONS FOR THE
LATENT CLASSES
BASED ON THEIR MOST LIKELY LATENT CLASS
MEMBERSHIP

Class Counts and Proportions

    Latent
   Classes

       1           6141          0.22088
       2          18658          0.67110
       3           3003          0.10801

CLASSIFICATION QUALITY

     Entropy                         0.854
```

```
Average Latent Class Probabilities for Most Likely Latent
Class Membership (Row)
by Latent Class (Column)

         1        2        3

   1   0.822    0.116    0.063
   2   0.026    0.974    0.000
   3   0.071    0.001    0.928

Classification Probabilities for the Most Likely Latent Class
Membership (Column)
by Latent Class (Row)

         1        2        3

   1   0.879    0.083    0.037
   2   0.038    0.962    0.000
   3   0.122    0.000    0.878

Logits for the Classification Probabilities for the Most Likely
Latent Class Membership (Column)
by Latent Class (Row)

            1        2        3

   1     3.164    0.809    0.000
   2     5.521    8.764    0.000
   3    -1.978  -10.865    0.000

MODEL RESULTS

                                          Two-Tailed
                   Estimate       S.E.  Est./S.E.    P-Value

Latent Class 1

 SLPNSSNC WITH
    WORRYNC            0.015      0.006      2.633      0.008
    SOLC               0.106      0.008     14.059      0.000
    SLPDURC            0.019      0.009      2.191      0.028

 WORRYNC  WITH
    SOLC               0.088      0.017      5.295      0.000
    SLPDURC           -0.048      0.010     -4.925      0.000

 SOLC     WITH
    SLPDURC            0.051      0.013      3.916      0.000

 Means
    SLPNSSNC           0.931      0.012     78.488      0.000
    WORRYNC           -0.775      0.027    -28.725      0.000
    SOLC               0.353      0.018     19.838      0.000
    SLPDURC            0.391      0.023     17.144      0.000

 Variances
    SLPNSSNC           0.468      0.003    152.809      0.000
    WORRYNC            0.210      0.009     23.619      0.000
    SOLC               0.626      0.013     47.707      0.000
    SLPDURC            0.798      0.010     76.790      0.000

Latent Class 2

 SLPNSSNC WITH
    WORRYNC           -0.030      0.004     -8.232      0.000
    SOLC               0.118      0.005     22.000      0.000
    SLPDURC            0.037      0.006      6.407      0.000

 WORRYNC  WITH
    SOLC              -0.192      0.008    -25.245      0.000
    SLPDURC           -0.187      0.010    -18.506      0.000

 SOLC     WITH
    SLPDURC            0.290      0.011     27.050      0.000

 Means
    SLPNSSNC           0.904      0.005    171.611      0.000
    WORRYNC            0.570      0.006    101.171      0.000
    SOLC              -0.364      0.009    -40.558      0.000
    SLPDURC           -0.300      0.007    -40.716      0.000

 Variances
    SLPNSSNC           0.468      0.003    152.809      0.000
    WORRYNC            0.210      0.009     23.619      0.000
    SOLC               0.626      0.013     47.707      0.000
    SLPDURC            0.798      0.010     76.790      0.000

Latent Class 3

 SLPNSSNC WITH
    WORRYNC            0.009      0.006      1.533      0.125
    SOLC               0.046      0.011      4.198      0.000
    SLPDURC            0.013      0.012      1.153      0.249

 WORRYNC  WITH
    SOLC              -0.006      0.006     -0.926      0.354
    SLPDURC           -0.070      0.009     -7.748      0.000

 SOLC     WITH
    SLPDURC            0.050      0.015      3.310      0.001

 Means
    SLPNSSNC           1.112      0.015     76.093      0.000
    WORRYNC           -1.994      0.027    -74.945      0.000
    SOLC               1.572      0.036     43.121      0.000
    SLPDURC            1.108      0.027     40.596      0.000

 Variances
    SLPNSSNC           0.468      0.003    152.809      0.000
    WORRYNC            0.210      0.009     23.619      0.000
    SOLC               0.626      0.013     47.707      0.000
    SLPDURC            0.798      0.010     76.790      0.000

Categorical Latent Variables

 Means
    C#1                0.593      0.046     12.891      0.000
    C#2                1.784      0.040     44.896      0.000

QUALITY OF NUMERICAL RESULTS

     Condition Number for the Information Matrix
0.903E-04
       (ratio of smallest to largest eigenvalue)

TECHNICAL 11 OUTPUT

     Random Starts Specifications for the k-1 Class Analysis
Model
        Number of initial stage random starts                1000
        Number of final stage optimizations                   250

     VUONG-LO-MENDELL-RUBIN LIKELIHOOD RATIO
TEST FOR 2 (H0) VERSUS 3 CLASSES

          H0 Loglikelihood Value                     -127763.908
          2 Times the Loglikelihood Difference          3538.985
          Difference in the Number of Parameters              11
          Mean                                   61.969
          Standard Deviation                             60.777
```

```
        P-Value                          0.0000

     LO-MENDELL-RUBIN ADJUSTED LRT TEST

        Value                          3507.822
        P-Value                          0.0000

TECHNICAL 14 OUTPUT

     Random Starts Specifications for the k-1 Class Analysis
Model
        Number of initial stage random starts              1000
        Number of final stage optimizations                 250

     Random Starts Specification for the k-1 Class Model for
Generated Data
        Number of initial stage random starts                 2
        Number of final stage optimizations                   1
     Random Starts Specification for the k Class Model for
Generated Data
        Number of initial stage random starts                50
        Number of final stage optimizations                  10
     Number of bootstrap draws requested                    250

     PARAMETRIC BOOTSTRAPPED LIKELIHOOD RATIO
TEST FOR 2 (H0) VERSUS 3 CLASSES

        H0 Loglikelihood Value                   -127763.908
        2 Times the Loglikelihood Difference        3538.985
        Difference in the Number of Parameters            11
        Approximate P-Value                           0.0000
        Successful Bootstrap Draws                       250

     WARNING:  OF THE 250 BOOTSTRAP DRAWS, 230
DRAWS HAD BOTH A SMALLER LRT VALUE THAN THE
     OBSERVED LRT VALUE AND NOT A REPLICATED
BEST LOGLIKELIHOOD VALUE FOR THE 3-CLASS
MODEL.
     THIS MEANS THAT THE P-VALUE MAY NOT BE
TRUSTWORTHY DUE TO LOCAL MAXIMA.
     INCREASE THE NUMBER OF RANDOM STARTS
USING THE LRTSTARTS OPTION.

PLOT INFORMATION

The following plots are available:

  Histograms (sample values)
  Scatterplots (sample values)
  Sample means
  Estimated means, medians, modes, and percentiles
  Sample and estimated means
  Observed individual values
  Estimated means and observed individual values
  Estimated overall and class-specific distributions

SAVEDATA INFORMATION

  Save file
    Step1_LPA3M5.dat

  Order and format of variables

    SLPNSSNC       F10.3
    WORRYNC        F10.3
    SOLC           F10.3
    SLPDURC        F10.3
    CPROB1         F10.3
    CPROB2         F10.3
    CPROB3         F10.3
    C              F10.3
    ID             I6

  Save file format
    8F10.3 I6

  Save file record length    10000

  Save missing symbol        *

DIAGRAM INFORMATION

  Mplus diagrams are currently not available for Mixture
analysis.
  No diagram output was produced.


     Beginning Time:  10:37:29
        Ending Time:  12:21:31
       Elapsed Time:  01:44:02

MUTHEN & MUTHEN
3463 Stoner Ave.
Los Angeles, CA  90066

Tel: (310) 391-9971
Fax: (310) 391-8971
Web: www.StatModel.com
Support: Support@StatModel.com

Copyright (c) 1998-2021 Muthen & Muthen
```

Parametrization Model 6 - Varying variances and varying covariances

```
Mplus VERSION 8.7
MUTHEN & MUTHEN
07/11/2023   5:32 PM

INPUT INSTRUCTIONS

  TITLE: LPA 3 profile syntax: M6: Varying means, varying
variances and varying covariances
  DATA:
  FILE IS mplus2.csv;
  ! Specifies file location for data file. Make sure data is in
format appropriate for Mplus
  ! per Mplus manual. This data file is in individual format
(one row of data per participan
  VARIABLE:
  NAMES ARE ID SCI_02 SCI_02B slpnssN worryN sol
slpDur gender genderN yrgrp yrgrpN SCI_02C
  slpnssNC worryNC solC slpDurC;

  ! All variables included in data file should be named here.
  USEVARIABLES ARE slpnssNC worryNC solC slpDurC;
  ! Only variables intended for use in the analysis should be
listed here
  IDVARIABLE IS ID;
  CLASSES = c (3);
  ! This is where you instruct Mplus on how many
classes/profiles are being estimated. Initi
  ! model contains only one class/profile, thus it would be
CLASSES = c (1). Above specifies
```

```
  !profiles, and for each further iterative models the number in parentheses increases by on
  !three profiles/classes would be c (3), and so on.
  MISSING ARE ALL(9999);
  ! Used to communicate how missing data is coded in data file. Here shown with a "." which
  ! all that is included in each cell with missing data in the data file
  ANALYSIS:
  TYPE = MIXTURE;
  ! LPA is a version of mixture modeling, and this instructs Mplus to analyze in this way
  ESTIMATOR = MLR;
  !FIML robust to non-normal data
  STARTS = 1000 250;
  STITERATIONS = 500;
  ! Default number of starts for each step of the ML estimation. First STARTS value specifie
  !number of unique start values to start with, the 250 represents the 250 best unique start
  !carrying forward to completion. The STITERATIONS specifies the number of ML iteration
  !steps for those 250 selected start values to go through to be able to converge. This is a
  !maximum number of iteration; if a model converges in less than 500 iterations it will sto
  !before reaching 500 iterations.
  !These values can be increased … see "Four-Profile Final Model with Covariate Analysis
  !Syntax" for an example.
  LRTSTARTS = 2 1 50 10;
  LRTBOOTSTRAP = 250;
  !The above start values are for the defaults for the LRT statistic being run to compare th
  !model fit with the model fit of a model with one less class (k-1). The BOOTSTRAP statemen
  !specifies the number of bootstrap draws to inform Mplus' bootstrapped LRT results.
  MODEL:
  !For a default Mplus model the LPA model does not need to be specified. However, it can be
  !The model can also be modified from the Mplus default of estimating the indicator means
  !(uniquely across profiles) and variances (constrained across profiles), as well as the la
  !profile mean.
  %OVERALL%
  [slpnssNC worryNC solC slpDurC]; !estimates the indicators means for each profile. Without
  !the means are freely estimated in each profile, not constrained.
  slpnssNC worryNC solC slpDurC; !Label Var1-Var5 constrains the estimates of the variances
  !profiles to be equal.

  %c#1%

     [slpnssNC worryNC solC slpDurC];

     slpnssNC worryNC solC slpDurC;
     !covariances
  slpnssNC WITH worryNC solC slpDurC;
  worryNC WITH solC slpDurC;
  solC WITH slpDurC;

   %c#2%

     [slpnssNC worryNC solC slpDurC];

     slpnssNC worryNC solC slpDurC;
     !covariances
  slpnssNC WITH worryNC solC slpDurC;
  worryNC WITH solC slpDurC;
  solC WITH slpDurC;

   %c#3%

     [slpnssNC worryNC solC slpDurC];

     slpnssNC worryNC solC slpDurC;
     !covariances
  slpnssNC WITH worryNC solC slpDurC;
  worryNC WITH solC slpDurC;
  solC WITH slpDurC;

  OUTPUT:
  TECH11 TECH14;
  ! TECH1 provides parameter specifications and starting values for the analysis
  ! TECH8 provides optimization history for this analysis type
  !TECH11 provides LRT results
  !TECH14 provides bootstrapped LRT test
  PLOT: SERIES=slpnssNC worryNC solC slpDurC(*);
  TYPE=PLOT3;
  SAVEDATA:
  FILE IS Step1_LPA3M5.dat;
  ! Tells Mplus where to save the output files from the analysis
  SAVE = CPROBABILITIES;
  ! The above command lines are to save the most likely profile membership for each particip
  ! and the posterior probabilities for their membership in each latent profile.

*** WARNING in MODEL command
  All variables are uncorrelated with all other variables within class.
  Check that this is what is intended.

LPA 3 profile syntax: M6: Varying means, varying variances, and varying covariances

SUMMARY OF ANALYSIS

Number of groups                                   1
Number of observations                         27802

Number of dependent variables                      4
Number of independent variables                    0
Number of continuous latent variables              0
Number of categorical latent variables             1

Observed dependent variables

  Continuous
   SLPNSSNC    WORRYNC     SOLC       SLPDURC

Categorical latent variables
   C

Variables with special functions

  ID variable          ID

Estimator                                        MLR
Information matrix                          OBSERVED
Optimization Specifications for the Quasi-Newton Algorithm for
Continuous Outcomes
  Maximum number of iterations                   100
```

Convergence criterion 0.100D-05
Optimization Specifications for the EM Algorithm
Maximum number of iterations 500
Convergence criteria
Loglikelihood change 0.100D-06
Relative loglikelihood change 0.100D-06
Derivative 0.100D-05
Optimization Specifications for the M step of the EM Algorithm for
Categorical Latent variables
Number of M step iterations 1
M step convergence criterion 0.100D-05
Basis for M step termination ITERATION
Optimization Specifications for the M step of the EM Algorithm for
Censored, Binary or Ordered Categorical (Ordinal), Unordered
Categorical (Nominal) and Count Outcomes
Number of M step iterations 1
M step convergence criterion 0.100D-05
Basis for M step termination ITERATION
Maximum value for logit thresholds 15
Minimum value for logit thresholds -15
Minimum expected cell size for chi-square 0.100D-01
Maximum number of iterations for H1 2000
Convergence criterion for H1 0.100D-03
Optimization algorithm EMA
Random Starts Specifications
Number of initial stage random starts 1000
Number of final stage optimizations 250
Number of initial stage iterations 500
Initial stage convergence criterion 0.100D+01
Random starts scale 0.500D+01
Random seed for generating random starts 0

Input data file(s)
mplus2.csv
Input data format FREE

SUMMARY OF DATA

Number of missing data patterns 4
Number of y missing data patterns 4
Number of u missing data patterns 0

COVARIANCE COVERAGE OF DATA

Minimum covariance coverage value 0.100

PROPORTION OF DATA PRESENT FOR Y

Covariance Coverage

	SLPNSSNC	WORRYNC	SOLC	SLPDURC
SLPNSSNC	1.000			
WORRYNC	1.000	1.000		
SOLC	0.976	0.976	0.976	
SLPDURC	0.963	0.963	0.940	0.963

UNIVARIATE SAMPLE STATISTICS

UNIVARIATE HIGHER-ORDER MOMENT DESCRIPTIVE STATISTICS

Variable/ Mean/ Skewness/ Minimum/ % with Percentiles
Sample Size Variance Kurtosis Maximum Min/Max 20%/60% 40%/80% Median

SLPNSSNC 0.933 0.086 0.000 27.10% 0.000 1.000 1.000
27802.000 0.471 -0.878 2.000 20.40% 1.000 2.000
WORRYNC 0.000 -1.036 -2.825 1.71% -0.928 0.020 0.495
27802.000 1.000 0.165 0.969 28.55% 0.495 0.969
SOLC 0.000 0.967 -1.054 31.63% -1.054 -0.072 -0.072
27123.000 1.000 0.467 2.875 2.77% -0.072 0.910
SLPDURC 0.000 0.568 -1.205 25.70% -1.205 -0.292 -0.292
26763.000 1.000 -0.470 2.445 3.44% -0.292 0.620

RANDOM STARTS RESULTS RANKED FROM THE BEST TO THE WORST LOGLIKELIHOOD VALUES

Unperturbed starting value run did not converge in the initial stage
optimizations.

683 perturbed starting value run(s) did not converge in the initial stage
optimizations.

Final stage loglikelihood values at local maxima, seeds, and initial stage start numbers:

-121739.377	459221	652
-121739.377	278692	342
-121739.377	508482	446
-121739.377	55115	408
-121739.377	39810	966
-121739.377	465160	862
-121739.377	673496	218
-121739.377	415931	10
-121739.377	765586	709
-121739.377	479273	156
-121739.377	985387	381
-121739.377	96941	890
-121739.377	589483	950
-121739.377	76337	76
-121739.377	140442	500
-121739.377	704798	530
-121739.377	702492	718
-121739.377	179022	687
-121739.377	654136	167
-121739.377	51375	148
-121739.377	392751	480
-121739.377	315029	471
-121739.377	1548	384
-121739.377	712702	684
-121739.377	200041	810
-121739.377	77210	712
-121739.377	805768	879
-121739.377	296162	856
-121739.377	57226	208
-121739.377	35191	703
-121739.377	425929	508
-121739.377	992389	77
-121739.377	318230	46
-121739.377	471040	403
-121739.377	638977	643
-121739.377	215353	164
-121739.377	751153	110
-121739.377	964570	701

-121739.377	723035	538
-121739.377	863094	147
-121739.377	685268	596
-121739.377	467561	819
-121739.377	471398	74
-121739.377	646573	741
-121739.377	996231	310
-121739.377	679832	302
-121739.377	251680	794
-121739.377	404042	675
-121739.377	546943	865
-121739.377	903420	5
-121739.377	148918	682
-121739.377	77571	980
-121739.377	238906	430
-121739.377	930872	277
-121739.377	278661	674
-121739.377	156536	245
-121739.377	758647	951
-121739.377	268217	83
-121739.377	549244	756
-121739.377	495366	964
-121739.377	329127	185
-121739.377	645052	910
-121739.377	84013	598
-121739.377	853781	716
-121739.377	584397	428
-121739.377	685657	69
-121739.377	775881	778
-121739.377	178475	231
-121739.377	622173	992
-121739.377	548245	818
-121739.377	36714	201
-121739.377	455617	242
-121739.377	94573	983
-121739.377	926762	704
-121739.377	792993	859
-121739.377	291149	536
-121739.377	421731	886
-121739.377	732596	320
-121739.377	124999	96
-121739.377	85734	411
-121739.377	484501	163
-121739.377	696830	668
-121739.377	399671	13
-121739.377	741888	138
-121739.377	3307	735
-121739.377	436806	883
-121739.377	283492	435
-121739.377	210139	991
-121739.377	399848	220
-121739.377	848163	47
-121739.377	297518	166
-121739.377	856536	144
-121739.377	587946	120
-121739.377	741484	441
-121739.377	967237	48
-121739.377	836515	341
-121739.377	317640	437
-121739.377	965639	463
-121739.377	520177	262
-121739.377	922596	456
-121739.377	860772	174
-121739.377	193042	316
-121739.377	114433	708
-121739.377	292884	103
-121739.377	898745	466
-121739.377	373505	88
-121739.377	150531	154
-121739.377	760599	832
-121739.377	798821	423
-121739.377	507218	613
-121739.377	903633	553
-121739.377	424223	900
-121739.377	81117	305
-121739.377	484116	915
-121739.377	86698	893
-121739.377	518828	432
-121739.377	70118	104
-121739.377	461866	722
-121739.377	850840	232
-121739.377	25127	107
-121739.377	131856	90
-121739.377	794236	127
-121739.377	402699	604
-121739.377	464179	106
-121739.377	178031	720
-121739.377	61587	400
-121739.377	797594	234
-121739.377	404426	344
-121739.377	545108	667
-121739.377	562716	300
-121739.377	391368	802
-121739.377	153053	378
-121739.377	995249	525
-121739.377	926797	406
-121739.377	458181	189
-121739.377	714455	476
-121739.377	829540	324
-121739.377	216565	474
-121739.377	462953	7
-121739.377	49293	707
-121739.377	478341	731
-121739.377	914505	838
-121739.377	266038	672
-121739.377	730868	977
-121739.377	478421	311
-121739.377	677062	680
-121739.377	544048	87
-121739.377	130011	587
-121739.377	690596	858
-121739.377	327140	678
-121739.377	484687	306
-121739.377	875667	367
-121739.377	42523	730
-121739.377	275475	413
-121739.377	168888	817
-121739.377	92564	583
-121739.377	534193	689
-121739.377	444228	860
-121739.377	344422	296
-121739.377	972873	157
-121739.377	264081	186
-121739.377	496710	386
-121739.377	535804	111
-121739.377	137377	397
-121739.377	626891	32
-121739.377	373815	618
-121739.377	534864	307
-121739.377	738393	619
-121739.377	376411	473
-121739.377	213532	503
-121739.377	153394	429
-121739.377	155749	960
-121739.377	136842	58
-121739.377	442072	625
-121739.377	724519	333
-121739.377	997222	229
-121739.377	650371	14
-121739.377	696773	80

```
-121739.377  863691     481
-121739.377  287107     864
-121739.377  349360     464
-121739.377  66276      217
-121739.377  121425     511
-121739.377  761302     744
-121739.377  804660     260
-121739.377  950604     172
-121739.377  824956     607
-121739.377  94610      884
-121739.377  848969     173
-121739.377  350608     334
-121739.377  813779     92
-121739.377  483369     270
-121739.377  655497     376
-121739.377  814975     129
-121739.377  659832     754
-121739.377  891531     206
-121739.377  97300      640
-121739.377  312754     562
-121739.377  125727     899
-121739.377  802644     874
-121739.377  565819     65
-121739.377  501995     791
-121739.377  830392     35
-121739.377  931874     141
-121739.377  566739     575
-121739.377  576596     99
-121739.377  202790     198
-121739.377  317868     740
-121739.377  566687     597
-121739.377  535063     329
-121739.377  576783     866
-121739.377  782179     835
-121739.377  749453     33
-121739.377  840031     276
-121739.377  636396     168
-121739.377  354559     73
-121739.377  197223     332
-121739.377  303834     798
-121739.377  746978     410
-121739.377  194143     510
-121739.377  227563     63
-121739.377  39756      857
-121739.377  107446     12
-121739.377  570681     777
-121739.377  298553     773
-121739.377  403801     762
-121739.377  872743     374
-121739.377  851945     18
-121739.377  539751     459
-121739.377  468036     131
-121739.377  358488     264
-121739.377  991329     182
-121739.377  263221     447
-121739.377  379823     905
-121739.377  783165     170
-121739.377  422103     62
-121739.377  241299     912
-121739.377  536551     696
-121739.377  354395     486
-121739.377  631413     439
-121739.377  865906     641
-121739.377  383902     673
-121739.377  39136      226
-121739.377  244349     736
-121739.377  967902     52
-121739.377  347222     533
-121739.377  476295     969
-121739.377  534483     290
-121739.377  769907     457
-121739.377  954914     911

THE BEST LOGLIKELIHOOD VALUE HAS BEEN
REPLICATED.  RERUN WITH AT LEAST TWICE THE
RANDOM STARTS TO CHECK THAT THE BEST
LOGLIKELIHOOD IS STILL OBTAINED AND
REPLICATED.

THE MODEL ESTIMATION TERMINATED NORMALLY

MODEL FIT INFORMATION

Number of Free Parameters                   44

Loglikelihood

        H0 Value                  -121739.377
        H0 Scaling Correction Factor      1.0687
          for MLR

Information Criteria

        Akaike (AIC)               243566.755
        Bayesian (BIC)             243929.001
        Sample-Size Adjusted BIC      243789.170
          (n* = (n + 2) / 24)

FINAL CLASS COUNTS AND PROPORTIONS FOR THE
LATENT CLASSES
BASED ON THE ESTIMATED MODEL

    Latent
   Classes

       1      9661.77408         0.34752
       2     10017.29963         0.36031
       3      8122.92629         0.29217

FINAL CLASS COUNTS AND PROPORTIONS FOR THE
LATENT CLASSES
BASED ON ESTIMATED POSTERIOR PROBABILITIES

    Latent
   Classes

       1      9661.77408         0.34752
       2     10017.29963         0.36031
       3      8122.92629         0.29217

FINAL CLASS COUNTS AND PROPORTIONS FOR THE
LATENT CLASSES
BASED ON THEIR MOST LIKELY LATENT CLASS
MEMBERSHIP

Class Counts and Proportions

    Latent
   Classes

       1          11507         0.41389
       2           9377         0.33728
       3           6918         0.24883

CLASSIFICATION QUALITY

     Entropy                         0.651

Average Latent Class Probabilities for Most Likely Latent
Class Membership (Row)
```

```
by Latent Class (Column)

        1       2       3

  1  0.812   0.021   0.167
  2  0.010   0.908   0.082
  3  0.032   0.182   0.786

Classification Probabilities for the Most Likely Latent Class
Membership (Column)
by Latent Class (Row)

        1       2       3

  1  0.968   0.010   0.023
  2  0.024   0.850   0.126
  3  0.236   0.095   0.669

Logits for the Classification Probabilities for the Most Likely
Latent Class Membership (Column)
by Latent Class (Row)

          1        2        3

  1     3.753   -0.856    0.000
  2    -1.653    1.909    0.000
  3    -1.043   -1.954    0.000

MODEL RESULTS

                                        Two-Tailed
               Estimate       S.E.  Est./S.E.    P-Value

Latent Class 1

 SLPNSSNC WITH
   WORRYNC         -0.006      0.002     -2.581      0.010
   SOLC             0.043      0.006      7.601      0.000
   SLPDURC         -0.008      0.005     -1.854      0.064

 WORRYNC  WITH
   SOLC            -0.047      0.005    -10.231      0.000
   SLPDURC         -0.016      0.002     -6.617      0.000

 SOLC     WITH
   SLPDURC          0.046      0.006      7.140      0.000

 Means
   SLPNSSNC         0.855      0.011     75.023      0.000
   WORRYNC          0.810      0.012     66.773      0.000
   SOLC            -0.636      0.030    -21.139      0.000
   SLPDURC         -0.744      0.016    -47.613      0.000

 Variances
   SLPNSSNC         0.434      0.006     76.347      0.000
   WORRYNC          0.051      0.002     21.918      0.000
   SOLC             0.259      0.015     17.583      0.000
   SLPDURC          0.236      0.005     50.873      0.000

Latent Class 2

 SLPNSSNC WITH
   WORRYNC         -0.013      0.009     -1.441      0.150
   SOLC             0.157      0.010     15.261      0.000
   SLPDURC          0.032      0.010      3.297      0.001

 WORRYNC  WITH
   SOLC            -0.245      0.012    -20.354      0.000
   SLPDURC         -0.336      0.014    -23.324      0.000

 SOLC     WITH
   SLPDURC          0.221      0.018     12.366      0.000

 Means
   SLPNSSNC         1.018      0.010    103.100      0.000
   WORRYNC         -0.992      0.021    -47.491      0.000
   SOLC             0.821      0.019     42.708      0.000
   SLPDURC          0.643      0.015     43.949      0.000

 Variances
   SLPNSSNC         0.488      0.005     95.892      0.000
   WORRYNC          0.897      0.019     46.060      0.000
   SOLC             1.167      0.016     72.683      0.000
   SLPDURC          1.068      0.013     82.995      0.000

Latent Class 3

 SLPNSSNC WITH
   WORRYNC          0.010      0.006      1.651      0.099
   SOLC             0.083      0.006     13.391      0.000
   SLPDURC          0.002      0.010      0.176      0.860

 WORRYNC  WITH
   SOLC            -0.046      0.011     -4.199      0.000
   SLPDURC          0.053      0.020      2.679      0.007

 SOLC     WITH
   SLPDURC          0.028      0.016      1.716      0.086

 Means
   SLPNSSNC         0.921      0.015     61.046      0.000
   WORRYNC          0.259      0.034      7.565      0.000
   SOLC            -0.239      0.030     -7.868      0.000
   SLPDURC          0.103      0.036      2.858      0.004

 Variances
   SLPNSSNC         0.477      0.006     83.275      0.000
   WORRYNC          0.193      0.013     14.489      0.000
   SOLC             0.319      0.011     29.756      0.000
   SLPDURC          0.648      0.016     40.366      0.000

Categorical Latent Variables

 Means
   C#1              0.173      0.110      1.581      0.114
   C#2              0.210      0.067      3.148      0.002

QUALITY OF NUMERICAL RESULTS

     Condition Number for the Information Matrix
0.661E-05
       (ratio of smallest to largest eigenvalue)

TECHNICAL 11 OUTPUT

     Random Starts Specifications for the k-1 Class Analysis
Model
        Number of initial stage random starts                  1000
        Number of final stage optimizations                     250

     VUONG-LO-MENDELL-RUBIN LIKELIHOOD RATIO
TEST FOR 2 (H0) VERSUS 3 CLASSES

          H0 Loglikelihood Value                    -123451.625
          2 Times the Loglikelihood Difference         3424.495
          Difference in the Number of Parameters             15
          Mean                                           54.570
          Standard Deviation                             60.046
          P-Value                                        0.0000
```

```
    LO-MENDELL-RUBIN ADJUSTED LRT TEST

        Value                             3402.329
        P-Value                             0.0000

TECHNICAL 14 OUTPUT

    Random Starts Specifications for the k-1 Class Analysis
Model
       Number of initial stage random starts             1000
       Number of final stage optimizations               250

    Random Starts Specification for the k-1 Class Model for
Generated Data
       Number of initial stage random starts                2
       Number of final stage optimizations                  1
    Random Starts Specification for the k Class Model for
Generated Data
       Number of initial stage random starts               50
       Number of final stage optimizations                 10
    Number of bootstrap draws requested                   250

    PARAMETRIC BOOTSTRAPPED LIKELIHOOD RATIO
TEST FOR 2 (H0) VERSUS 3 CLASSES

        H0 Loglikelihood Value                  -123451.625
        2 Times the Loglikelihood Difference          3424.495
        Difference in the Number of Parameters            15
        Approximate P-Value                         0.0000
        Successful Bootstrap Draws                     250

    WARNING:  OF THE 250 BOOTSTRAP DRAWS, 224
DRAWS HAD BOTH A SMALLER LRT VALUE THAN THE
    OBSERVED LRT VALUE AND NOT A REPLICATED
BEST LOGLIKELIHOOD VALUE FOR THE 3-CLASS
MODEL.
    THIS MEANS THAT THE P-VALUE MAY NOT BE
TRUSTWORTHY DUE TO LOCAL MAXIMA.
    INCREASE THE NUMBER OF RANDOM STARTS
USING THE LRTSTARTS OPTION.

PLOT INFORMATION

The following plots are available:

  Histograms (sample values)
  Scatterplots (sample values)
  Sample means
  Estimated means, medians, modes, and percentiles
  Sample and estimated means
  Observed individual values
  Estimated means and observed individual values
  Estimated overall and class-specific distributions

SAVEDATA INFORMATION

  Save file
    Step1_LPA3M5.dat

  Order and format of variables

    SLPNSSNC      F10.3
    WORRYNC       F10.3
    SOLC          F10.3
    SLPDURC       F10.3
    CPROB1        F10.3
    CPROB2        F10.3
    CPROB3        F10.3
    C             F10.3
    ID            I6

  Save file format
    8F10.3 I6

  Save file record length    10000

  Save missing symbol       *

DIAGRAM INFORMATION

  Mplus diagrams are currently not available for Mixture
analysis.
  No diagram output was produced.

     Beginning Time:  17:32:22
        Ending Time:  22:33:21
       Elapsed Time:  05:00:59

MUTHEN & MUTHEN
3463 Stoner Ave.
Los Angeles, CA  90066

Tel: (310) 391-9971
Fax: (310) 391-8971
Web: www.StatModel.com
Support: Support@StatModel.com

Copyright (c) 1998-2021 Muthen & Muthen
```

Step 2: Profile Selection

Series of LPA models were conducted to assess the number of profiles based on participants' SOL, sleep duration, daytime sleepiness and worry disrupting sleep. Each model was compared against the previous model (k-1 classes) in an iterative process to decide on the number of latent profiles (one to six) to be included. Two main indices were used, in line with existing LPA guidelines, to determine the appropriate number of profiles and whether additional profiles in the LPA model improved the model fit: Bayesian Information Criterion (BIC), and the Vuong-Lo-Mendell-Rubin adjusted likelihood ratio test (VLMR-LRT). A lower BIC value represented the preferred model. The VLMR-LRT test assists in determining when additional profiles are not an improvement and compares significance between an estimated model versus a model with one fewer profile. A non-significant VLMR-LRT

test suggests that the more parsimonious model (fewer profiles) is the better fitting and representative model. Other model fit indicators including entropy index and smallest class size were also examined as diagnostic criteria. An entropy value of > .80 is desired, but values between .60 and .80 were treated as acceptable. Smallest profile size was examined and n < 30 was considered not sufficient to support generalizability.

Two-profile model

```
Mplus VERSION 8.7
MUTHEN & MUTHEN
07/13/2023   2:09 PM

INPUT INSTRUCTIONS

  TITLE: LPA 2 profile syntax: M5: Varying means, equal
variances, and varying covariances

  DATA:
  FILE IS mplus2.csv;
  ! Specifies file location for data file. Make sure data is in
format appropriate for Mplus
  ! per Mplus manual. This data file is in individual format
(one row of data per participan
  VARIABLE:
  NAMES ARE ID SCI_02 SCI_02B slpnssN worryN sol
slpDur gender genderN yrgrp yrgrpN SCI_02C
  slpnssNC worryNC solC slpDurC;

  ! All variables included in data file should be named here.
  USEVARIABLES ARE slpnssNC worryNC solC slpDurC;
  ! Only variables intended for use in the analysis should be
listed here
  IDVARIABLE IS ID;
  CLASSES = c (2);
  ! This is where you instruct Mplus on how many
classes/profiles are being estimated. Initi
  ! model contains only one class/profile, thus it would be
CLASSES = c (1). Above specifies
  !profiles, and for each further iterative models the number
in parentheses increases by on
  !three profiles/classes would be c (3), and so on.
  MISSING ARE ALL(9999);
  ! Used to communicate how missing data is coded in data
file. Here shown with a "." which
  ! all that is included in each cell with missing data in the
data file
  ANALYSIS:
  TYPE = MIXTURE;
  ! LPA is a version of mixture modeling, and this instructs
Mplus to analyze in this way
  ESTIMATOR = MLR;
  !FIML robust to non-normal data
  STARTS = 1000 250;
  STITERATIONS = 500;
  ! Default number of starts for each step of the ML
estimation. First STARTS value specifie
  !number of unique start values to start with, the 250
represents the 250 best unique start
  !carrying forward to completion. The STITERATIONS
specifies the number of ML iteration
  !steps for those 250 selected start values to go through to
be able to converge. This is a
  !maximum number of iteration; if a model converges in less
than 500 iterations it will sto
  !before reaching 500 iterations.
  !These values can be increased … see "Four-Profile Final
Model with Covariate Analysis
  !Syntax" for an example.
  LRTSTARTS = 2 1 50 10;
  LRTBOOTSTRAP = 250;
  !The above start values are for the defaults for the LRT
statistic being run to compare th
  !model fit with the model fit of a model with one less class
(k-1). The BOOTSTRAP statemen
  !specifies the number of bootstrap draws to inform Mplus'
bootstrapped LRT results.
  MODEL:
  !For a default Mplus model the LPA model does not need
to be specified. However, it can be
  !The model can also be modified from the Mplus default of
estimating the indicator means
  !(uniquely across profiles) and variances (constrained
across profiles), as well as the la
  !profile mean.
  %OVERALL%
  [slpnssNC worryNC solC slpDurC]; !estimates the
indicators means for each profile. Without
  !the means are freely estimated in each profile, not
constrained.
  slpnssNC worryNC solC slpDurC; !Label Var1-Var5
constrains the estimates of the variances
  !profiles to be equal.

  %c#1%

    [slpnssNC worryNC solC slpDurC];

    !covariances
  slpnssNC WITH worryNC solC slpDurC;
  worryNC WITH solC slpDurC;
  solC WITH slpDurC;

   %c#2%

    [slpnssNC worryNC solC slpDurC];

    !covariances
  slpnssNC WITH worryNC solC slpDurC;
  worryNC WITH solC slpDurC;
  solC WITH slpDurC;


  OUTPUT:
  TECH11 TECH14;
  ! TECH1 provides parameter specifications and starting
values for the analysis
  ! TECH8 provides optimization history for this analysis type
  !TECH11 provides LRT results
  !TECH14 provides bootstrapped LRT test
  PLOT: SERIES=slpnssNC worryNC solC slpDurC(*);
  TYPE=PLOT3;
  SAVEDATA:
  FILE IS Step2_LPA2M5.dat;
```

```
  ! Tells Mplus where to save the output files from the
analysis
  SAVE = CPROBABILITIES;
  ! The above command lines are to save the most likely
profile membership for each particip
  ! and the posterior probabilities for their membership in
each latent profile.

*** WARNING in MODEL command
  All variables are uncorrelated with all other variables within
class.
  Check that this is what is intended.

LPA 2 profile syntax: M5: Varying means, equal variances,
and varying covariances

SUMMARY OF ANALYSIS

Number of groups                                  1
Number of observations                        27802

Number of dependent variables                     4
Number of independent variables                   0
Number of continuous latent variables             0
Number of categorical latent variables            1

Observed dependent variables

  Continuous
   SLPNSSNC    WORRYNC     SOLC       SLPDURC

Categorical latent variables
   C

Variables with special functions

  ID variable          ID

Estimator                                       MLR
Information matrix                         OBSERVED
Optimization Specifications for the Quasi-Newton Algorithm
for
Continuous Outcomes
  Maximum number of iterations                  100
  Convergence criterion                   0.100D-05
Optimization Specifications for the EM Algorithm
  Maximum number of iterations                  500
  Convergence criteria
    Loglikelihood change                  0.100D-06
    Relative loglikelihood change         0.100D-06
    Derivative                            0.100D-05
Optimization Specifications for the M step of the EM
Algorithm for
Categorical Latent variables
  Number of M step iterations                     1
  M step convergence criterion            0.100D-05
  Basis for M step termination            ITERATION
Optimization Specifications for the M step of the EM
Algorithm for
Censored, Binary or Ordered Categorical (Ordinal),
Unordered
Categorical (Nominal) and Count Outcomes
  Number of M step iterations                     1
  M step convergence criterion            0.100D-05
  Basis for M step termination            ITERATION
  Maximum value for logit thresholds             15
  Minimum value for logit thresholds            -15
  Minimum expected cell size for chi-square  0.100D-
01
Maximum number of iterations for H1            2000
Convergence criterion for H1              0.100D-03
Optimization algorithm                          EMA
Random Starts Specifications
  Number of initial stage random starts        1000
  Number of final stage optimizations           250
  Number of initial stage iterations            500
  Initial stage convergence criterion     0.100D+01
  Random starts scale                     0.500D+01
  Random seed for generating random starts        0

Input data file(s)
  mplus2.csv
Input data format  FREE

SUMMARY OF DATA

     Number of missing data patterns           4
     Number of y missing data patterns         4
     Number of u missing data patterns         0

COVARIANCE COVERAGE OF DATA

Minimum covariance coverage value   0.100

     PROPORTION OF DATA PRESENT FOR Y

           Covariance Coverage
              SLPNSSNC      WORRYNC       SOLC
SLPDURC
              ________      ________      ________      ________
 SLPNSSNC       1.000
 WORRYNC        1.000         1.000
 SOLC           0.976         0.976         0.976
 SLPDURC        0.963         0.963         0.940         0.963

UNIVARIATE SAMPLE STATISTICS

     UNIVARIATE HIGHER-ORDER MOMENT
DESCRIPTIVE STATISTICS

         Variable/         Mean/     Skewness/   Minimum/ % with
Percentiles
        Sample Size      Variance    Kurtosis    Maximum
Min/Max      20%/60%    40%/80%    Median

     SLPNSSNC              0.933       0.086       0.000   27.10%
0.000      1.000      1.000
           27802.000       0.471      -0.878       2.000   20.40%
1.000      2.000
     WORRYNC               0.000      -1.036      -2.825    1.71%
-0.928      0.020      0.495
           27802.000       1.000       0.165       0.969   28.55%
0.495      0.969
     SOLC                  0.000       0.967      -1.054   31.63%      -
1.054     -0.072     -0.072
           27123.000       1.000       0.467       2.875    2.77%
-0.072      0.910
     SLPDURC               0.000       0.568      -1.205   25.70%
-1.205     -0.292     -0.292
           26763.000       1.000      -0.470       2.445    3.44%
-0.292      0.620

RANDOM STARTS RESULTS RANKED FROM THE BEST
TO THE WORST LOGLIKELIHOOD VALUES

137 perturbed starting value run(s) did not converge in the
initial stage
optimizations.
```

```
Final stage loglikelihood values at local maxima, seeds, and
initial stage start numbers:

      -127763.908  512836           289
      -127763.908  327140           678
      -127763.908  341519           653
      -127763.908  185071           370
      -127763.908  265218           924
      -127763.908  96617            955
      -127763.908  847088           750
      -127763.908  117391           710
      -127763.908  580181           691
      -127763.908  345726           461
      -127763.908  995913           787
      -127763.908  845580           805
      -127763.908  728038           177
      -127763.908  86651            993
      -127763.908  458181           189
      -127763.908  792993           859
      -127763.908  715255           523
      -127763.908  17359            227
      -127763.908  574942           558
      -127763.908  488125           368
      -127763.908  599729           658
      -127763.908  399848           220
      -127763.908  779820           877
      -127763.908  327927           908
      -127763.908  496344           808
      -127763.908  369602           146
      -127763.908  985387           381
      -127763.908  50887            389
      -127763.908  546943           865
      -127763.908  692169           308
      -127763.908  370481           742
      -127763.908  573096           20
      -127763.908  629320           222
      -127763.908  742609           531
      -127763.908  881886           608
      -127763.908  902278           21
      -127763.908  150531           154
      -127763.908  939870           655
      -127763.908  403801           762
      -127763.908  152496           123
      -127763.908  440395           917
      -127763.908  903633           553
      -127763.908  596257           405
      -127763.908  136842           58
      -127763.908  77571            980
      -127763.908  863094           147
      -127763.908  833196           715
      -127763.908  344422           296
      -127763.908  529496           343
      -127763.908  232559           136
      -127763.908  137305           379
      -127763.908  848890           95
      -127763.908  723775           97
      -127763.908  549244           756
      -127763.908  379729           512
      -127763.908  533738           11
      -127763.908  699834           723
      -127763.908  628143           854
      -127763.908  738393           619
      -127763.908  231281           542
      -127763.908  645664           39
      -127763.908  508445           946
      -127763.908  298553           773
      -127763.908  416463           467
      -127763.908  690596           858
      -127763.908  160326           546
      -127763.908  928287           197
      -127763.908  448829           938
      -127763.908  392418           28
      -127763.908  485483           498
      -127763.908  425149           878
      -127763.908  726744           939
      -127763.908  314084           81
      -127763.908  791285           416
      -127763.908  193042           316
      -127763.908  520177           262
      -127763.908  816765           959
      -127763.908  781489           627
      -127763.908  673496           218
      -127763.908  566687           597
      -127763.908  297518           166
      -127763.908  424223           900
      -127763.908  857799           315
      -127763.908  88437            761
      -127763.908  252346           528
      -127763.908  192071           142
      -127763.908  682718           733
      -127763.908  536551           696
      -127763.908  217744           326
      -127763.908  688839           273
      -127763.908  227563           63
      -127763.908  395754           388
      -127763.908  519357           559
      -127763.908  926762           704
      -127763.908  225995           847
      -127763.908  798839           312
      -127763.908  206099           363
      -127763.908  81117            305
      -127763.908  579138           706
      -127763.908  937588           293
      -127763.908  686384           690
      -127763.908  126371           526
      -127763.908  794236           127
      -127763.908  350608           334
      -127763.908  484116           915
      -127763.908  857122           889
      -127763.908  576726           280
      -127763.908  17896            592
      -127763.908  518828           432
      -127763.908  121425           511
      -127763.908  170954           86
      -127763.908  520865           763
      -127763.908  677062           680
      -127763.908  626087           314
      -127763.908  131063           843
      -127763.908  307843           664
      -127763.908  405079           68
      -127763.908  850840           232
      -127763.908  132336           362
      -127763.908  393232           152
      -127763.908  836066           372
      -127763.908  402699           604
      -127763.908  241197           747
      -127763.908  963053           43
      -127763.908  105435           265
      -127763.908  856612           700
      -127763.908  669634           335
      -127763.908  485256           371
      -127763.908  552272           654
      -127763.908  294669           501
      -127763.908  85462            51
      -127763.908  462953           7
      -127763.908  668003           647
      -127763.908  259507           53
      -127763.908  715561           125
      -127763.908  804660           260
      -127763.908  967237           48
```

-127763.908	625191	350
-127763.908	954914	911
-127763.908	288738	940
-127763.908	802682	419
-127763.908	622173	992
-127763.908	85114	385
-127763.908	30098	209
-127763.908	231400	738
-127763.908	96941	890
-127763.908	407108	366
-127763.908	972430	491
-127763.908	760878	249
-127763.908	838615	677
-127763.908	872743	374
-127763.908	742688	594
-127763.908	882750	646
-127763.908	425982	485
-127763.908	400319	887
-127763.908	915107	54
-127763.908	761302	744
-127763.908	695453	902
-127763.908	622290	880
-127763.908	134830	780
-127763.908	22874	588
-127763.908	995249	525
-127763.908	992389	77
-127763.908	650371	14
-127763.908	685657	69
-127763.908	399508	415
-127763.908	907810	795
-127763.908	264081	186
-127763.908	298201	903
-127763.908	751153	110
-127763.908	347222	533
-127763.908	527050	948
-127763.908	535063	329
-127763.908	735928	916
-127763.908	399380	436
-127763.908	246575	291
-127763.908	147440	514
-127763.908	739214	807
-127763.908	197223	332
-127763.908	871438	561
-127763.908	659832	754
-127763.908	63231	935
-127763.908	414828	322
-127763.908	153053	378
-127763.908	345974	622
-127763.908	942358	644
-127763.908	848331	137
-127763.908	481835	57
-127763.908	459221	652
-127763.908	210139	991
-127763.908	848969	173
-127763.908	213532	503
-127763.908	939021	8
-127763.908	326091	759
-127763.908	829540	324
-127763.908	442072	625
-127763.908	860772	174
-127763.908	505244	582
-127763.908	580539	786
-127763.908	97300	640
-127763.908	790452	303
-127763.908	830292	527
-127763.908	852283	616
-127763.908	421731	886
-127763.908	897782	545
-127763.908	595153	230
-127763.908	422103	62
-127763.908	712702	684
-127763.908	217130	443
-127763.908	462662	872
-127763.908	466971	109
-127763.908	836515	341
-127763.908	360419	356
-127763.908	163110	584
-127763.908	287107	864
-127763.908	264521	929
-127763.908	354395	486
-127763.908	220492	743
-127763.908	11397	944
-127763.908	180889	920
-127763.908	238906	430
-127763.908	358488	264
-127763.908	648035	836
-127763.908	563584	657
-127763.908	618760	489
-127763.908	758647	951
-127763.908	862607	468
-127763.908	314757	345
-127763.908	193847	354
-127763.908	914505	838
-127763.908	327475	518
-127763.908	264901	634
-127763.908	373815	618
-127763.908	957392	79
-127763.908	521575	313
-127763.908	971853	402
-127763.908	548245	818
-127763.908	474357	789
-127763.908	268896	124
-127763.908	856536	144
-127763.908	963967	941
-127763.908	42523	730
-127763.908	411615	171
-127763.908	804561	59
-127763.908	372176	23
-127763.908	425929	508
-127763.908	376411	473
-127763.908	569131	26
-127763.908	748692	204
-127763.908	562716	300

THE BEST LOGLIKELIHOOD VALUE HAS BEEN REPLICATED. RERUN WITH AT LEAST TWICE THE RANDOM STARTS TO CHECK THAT THE BEST LOGLIKELIHOOD IS STILL OBTAINED AND REPLICATED.

THE MODEL ESTIMATION TERMINATED NORMALLY

MODEL FIT INFORMATION

Number of Free Parameters 25

Loglikelihood

H0 Value -127763.908
H0 Scaling Correction Factor 1.1651
for MLR

Information Criteria

Akaike (AIC) 255577.815
Bayesian (BIC) 255783.637
Sample-Size Adjusted BIC 255704.188
(n* = (n + 2) / 24)

```
FINAL CLASS COUNTS AND PROPORTIONS FOR THE
LATENT CLASSES
BASED ON THE ESTIMATED MODEL

    Latent
   Classes

     1      6498.62894          0.23375
     2     21303.37106          0.76625

FINAL CLASS COUNTS AND PROPORTIONS FOR THE
LATENT CLASSES
BASED ON ESTIMATED POSTERIOR PROBABILITIES

    Latent
   Classes

     1      6498.62894          0.23375
     2     21303.37106          0.76625

FINAL CLASS COUNTS AND PROPORTIONS FOR THE
LATENT CLASSES
BASED ON THEIR MOST LIKELY LATENT CLASS
MEMBERSHIP

Class Counts and Proportions

    Latent
   Classes

     1           6336          0.22790
     2          21466          0.77210

CLASSIFICATION QUALITY

     Entropy                        0.878

Average Latent Class Probabilities for Most Likely Latent
Class Membership (Row)
by Latent Class (Column)

        1        2

  1   0.940    0.060
  2   0.025    0.975

Classification Probabilities for the Most Likely Latent Class
Membership (Column)
by Latent Class (Row)

        1        2

  1   0.917    0.083
  2   0.018    0.982


Logits for the Classification Probabilities for the Most Likely
Latent Class Membership (Column)
by Latent Class (Row)

          1        2

  1     2.399    0.000
  2    -4.013    0.000


MODEL RESULTS

                                        Two-Tailed
                   Estimate       S.E.  Est./S.E.    P-Value

Latent Class 1

 SLPNSSNC WITH
    WORRYNC          -0.024      0.005     -5.002      0.000
    SOLC              0.113      0.007     16.449      0.000
    SLPDURC           0.042      0.008      5.542      0.000

 WORRYNC  WITH
    SOLC             -0.175      0.007    -25.662      0.000
    SLPDURC          -0.139      0.007    -20.205      0.000

 SOLC     WITH
    SLPDURC           0.169      0.010     17.225      0.000

 Means
    SLPNSSNC          0.989      0.010    100.111      0.000
    WORRYNC          -1.520      0.012   -122.617      0.000
    SOLC              0.816      0.018     45.861      0.000
    SLPDURC           0.743      0.016     46.134      0.000

 Variances
    SLPNSSNC          0.471      0.003    155.408      0.000
    WORRYNC           0.305      0.003     99.427      0.000
    SOLC              0.820      0.009     92.599      0.000
    SLPDURC           0.859      0.008    105.661      0.000

Latent Class 2

 SLPNSSNC WITH
    WORRYNC          -0.039      0.003    -11.465      0.000
    SOLC              0.136      0.005     26.590      0.000
    SLPDURC           0.042      0.005      8.336      0.000

 WORRYNC  WITH
    SOLC             -0.283      0.005    -52.165      0.000
    SLPDURC          -0.252      0.005    -46.861      0.000

 SOLC     WITH
    SLPDURC           0.357      0.008     42.220      0.000

 Means
    SLPNSSNC          0.916      0.005    189.197      0.000
    WORRYNC           0.464      0.005    102.502      0.000
    SOLC             -0.243      0.006    -39.804      0.000
    SLPDURC          -0.222      0.007    -33.454      0.000

 Variances
    SLPNSSNC          0.471      0.003    155.408      0.000
    WORRYNC           0.305      0.003     99.427      0.000
    SOLC              0.820      0.009     92.599      0.000
    SLPDURC           0.859      0.008    105.661      0.000

Categorical Latent Variables

 Means
    C#1              -1.187      0.018    -67.816      0.000


QUALITY OF NUMERICAL RESULTS

     Condition Number for the Information Matrix
0.563E-02
       (ratio of smallest to largest eigenvalue)


TECHNICAL 11 OUTPUT

     Random Starts Specifications for the k-1 Class Analysis
Model
```

```
        Number of initial stage random starts              1000
        Number of final stage optimizations                 250

     VUONG-LO-MENDELL-RUBIN LIKELIHOOD RATIO
TEST FOR 1 (H0) VERSUS 2 CLASSES

          H0 Loglikelihood Value                  -132903.306
          2 Times the Loglikelihood Difference        10278.796
          Difference in the Number of Parameters           11
          Mean                                 17.872
          Standard Deviation                        10.707
          P-Value                                0.0000

     LO-MENDELL-RUBIN ADJUSTED LRT TEST

          Value                              10188.283
          P-Value                                0.0000

TECHNICAL 14 OUTPUT

     Random Starts Specifications for the k-1 Class Analysis
Model
        Number of initial stage random starts              1000
        Number of final stage optimizations                 250

     Random Starts Specification for the k-1 Class Model for
Generated Data
        Number of initial stage random starts                 2
        Number of final stage optimizations                   1
     Random Starts Specification for the k Class Model for
Generated Data
        Number of initial stage random starts                50
        Number of final stage optimizations                  10
     Number of bootstrap draws requested                    250


     PARAMETRIC BOOTSTRAPPED LIKELIHOOD RATIO
TEST FOR 1 (H0) VERSUS 2 CLASSES

          H0 Loglikelihood Value                  -132903.306
          2 Times the Loglikelihood Difference        10278.796
          Difference in the Number of Parameters           11
          Approximate P-Value                        0.0000
          Successful Bootstrap Draws                      249

     WARNING:  OF THE 249 BOOTSTRAP DRAWS, 225
DRAWS HAD BOTH A SMALLER LRT VALUE THAN THE
     OBSERVED LRT VALUE AND NOT A REPLICATED
BEST LOGLIKELIHOOD VALUE FOR THE 2-CLASS
MODEL.
     THIS MEANS THAT THE P-VALUE MAY NOT BE
TRUSTWORTHY DUE TO LOCAL MAXIMA.
     INCREASE THE NUMBER OF RANDOM STARTS
USING THE LRTSTARTS OPTION.

     WARNING:  1 OUT OF 250 BOOTSTRAP DRAWS DID
NOT CONVERGE.
     INCREASE THE NUMBER OF RANDOM STARTS
USING THE LRTSTARTS OPTION.

PLOT INFORMATION

The following plots are available:

  Histograms (sample values)
  Scatterplots (sample values)
  Sample means
  Estimated means, medians, modes, and percentiles
  Sample and estimated means
  Observed individual values
  Estimated means and observed individual values
  Estimated overall and class-specific distributions

SAVEDATA INFORMATION

  Save file
    Step2_LPA2M5.dat

  Order and format of variables

    SLPNSSNC       F10.3
    WORRYNC        F10.3
    SOLC           F10.3
    SLPDURC        F10.3
    CPROB1         F10.3
    CPROB2         F10.3
    C              F10.3
    ID             I6

  Save file format
    7F10.3 I6

  Save file record length    10000

  Save missing symbol        *

DIAGRAM INFORMATION

  Mplus diagrams are currently not available for Mixture
analysis.
  No diagram output was produced.

     Beginning Time:  14:09:11
        Ending Time:  15:19:05
       Elapsed Time:  01:09:54

MUTHEN & MUTHEN
3463 Stoner Ave.
Los Angeles, CA  90066

Tel: (310) 391-9971
Fax: (310) 391-8971
Web: www.StatModel.com
Support: Support@StatModel.com

Copyright (c) 1998-2021 Muthen & Muthen
```

Three-profile model

```
Mplus VERSION 8.7
MUTHEN & MUTHEN
07/20/2023   9:24 AM

INPUT INSTRUCTIONS

  TITLE: LPA 3 profile syntax: M5: Varying means, equal
variances, and varying covariances

  DATA:
  FILE IS mplus2.csv;
  ! Specifies file location for data file. Make sure data is in
format appropriate for Mplus
  ! per Mplus manual. This data file is in individual format
(one row of data per participan
  VARIABLE:
```

```
  NAMES ARE ID SCI_02 SCI_02B slpnssN worryN sol
slpDur gender genderN yrgrp yrgrpN SCI_02C
  slpnssNC worryNC solC slpDurC;

  ! All variables included in data file should be named here.
  USEVARIABLES ARE slpnssNC worryNC solC slpDurC;
  ! Only variables intended for use in the analysis should be
listed here
  IDVARIABLE IS ID;
  CLASSES = c (3);
  ! This is where you instruct Mplus on how many
classes/profiles are being estimated. Initi
  ! model contains only one class/profile, thus it would be
CLASSES = c (1). Above specifies
  !profiles, and for each further iterative models the number
in parentheses increases by on
  !three profiles/classes would be c (3), and so on.
  MISSING ARE ALL(9999);
  ! Used to communicate how missing data is coded in data
file. Here shown with a "." which
  ! all that is included in each cell with missing data in the
data file
  ANALYSIS:
  TYPE = MIXTURE;
  ! LPA is a version of mixture modeling, and this instructs
Mplus to analyze in this way
  ESTIMATOR = MLR;
  !FIML robust to non-normal data
  STARTS = 1000 250;
  STITERATIONS = 500;
  ! Default number of starts for each step of the ML
estimation. First STARTS value specifie
  !number of unique start values to start with, the 250
represents the 250 best unique start
  !carrying forward to completion. The STITERATIONS
specifies the number of ML iteration
  !steps for those 250 selected start values to go through to
be able to converge. This is a
  !maximum number of iteration; if a model converges in less
than 500 iterations it will sto
  !before reaching 500 iterations.
  !These values can be increased … see "Four-Profile Final
Model with Covariate Analysis
  !Syntax" for an example.
  LRTSTARTS = 2 1 50 10;
  LRTBOOTSTRAP = 250;
  !The above start values are for the defaults for the LRT
statistic being run to compare th
  !model fit with the model fit of a model with one less class
(k-1). The BOOTSTRAP statemen
  !specifies the number of bootstrap draws to inform Mplus'
bootstrapped LRT results.
  MODEL:
  !For a default Mplus model the LPA model does not need
to be specified. However, it can be
  !The model can also be modified from the Mplus default of
estimating the indicator means
  !(uniquely across profiles) and variances (constrained
across profiles), as well as the la
  !profile mean.
  %OVERALL%
  [slpnssNC worryNC solC slpDurC]; !estimates the
indicators means for each profile. Without
  !the means are freely estimated in each profile, not
constrained.
  slpnssNC worryNC solC slpDurC; !Label Var1-Var5
constrains the estimates of the variances
  !profiles to be equal.

  %c#1%

     [slpnssNC worryNC solC slpDurC];

     !covariances
  slpnssNC WITH worryNC solC slpDurC;
  worryNC WITH solC slpDurC;
  solC WITH slpDurC;

   %c#2%

     [slpnssNC worryNC solC slpDurC];

     !covariances
  slpnssNC WITH worryNC solC slpDurC;
  worryNC WITH solC slpDurC;
  solC WITH slpDurC;

   %c#3%

     [slpnssNC worryNC solC slpDurC];

     !covariances
  slpnssNC WITH worryNC solC slpDurC;
  worryNC WITH solC slpDurC;
  solC WITH slpDurC;

  OUTPUT:
  TECH11 TECH14;
  ! TECH1 provides parameter specifications and starting
values for the analysis
  ! TECH8 provides optimization history for this analysis type
  !TECH11 provides LRT results
  !TECH14 provides bootstrapped LRT test
  PLOT: SERIES=slpnssNC worryNC solC slpDurC(*);
  TYPE=PLOT3;
  SAVEDATA:
  FILE IS Step1_LPA3M5.dat;
  ! Tells Mplus where to save the output files from the
analysis
  SAVE = CPROBABILITIES;
  ! The above command lines are to save the most likely
profile membership for each particip
  ! and the posterior probabilities for their membership in
each latent profile.

*** WARNING in MODEL command
  All variables are uncorrelated with all other variables within
class.
  Check that this is what is intended.

LPA 3 profile syntax: M5: Varying means, equal variances,
and varying covariances

SUMMARY OF ANALYSIS

Number of groups                                 1
Number of observations                       27802

Number of dependent variables                    4
Number of independent variables                  0
Number of continuous latent variables            0
Number of categorical latent variables           1

Observed dependent variables

  Continuous
   SLPNSSNC    WORRYNC     SOLC       SLPDURC

Categorical latent variables
   C
```

```
Variables with special functions

  ID variable           ID

Estimator                                          MLR
Information matrix                                 OBSERVED
Optimization Specifications for the Quasi-Newton Algorithm for
Continuous Outcomes
  Maximum number of iterations                        100
  Convergence criterion                          0.100D-05
Optimization Specifications for the EM Algorithm
  Maximum number of iterations                        500
  Convergence criteria
    Loglikelihood change                         0.100D-06
    Relative loglikelihood change                0.100D-06
    Derivative                                   0.100D-05
Optimization Specifications for the M step of the EM Algorithm for
Categorical Latent variables
  Number of M step iterations                           1
  M step convergence criterion                   0.100D-05
  Basis for M step termination                   ITERATION
Optimization Specifications for the M step of the EM Algorithm for
Censored, Binary or Ordered Categorical (Ordinal), Unordered
Categorical (Nominal) and Count Outcomes
  Number of M step iterations                           1
  M step convergence criterion                   0.100D-05
  Basis for M step termination                   ITERATION
  Maximum value for logit thresholds                   15
  Minimum value for logit thresholds                  -15
  Minimum expected cell size for chi-square      0.100D-01
Maximum number of iterations for H1                  2000
Convergence criterion for H1                     0.100D-03
Optimization algorithm                                EMA
Random Starts Specifications
  Number of initial stage random starts              1000
  Number of final stage optimizations                 250
  Number of initial stage iterations                  500
  Initial stage convergence criterion            0.100D+01
  Random starts scale                            0.500D+01
  Random seed for generating random starts              0

Input data file(s)
  mplus2.csv
Input data format  FREE

SUMMARY OF DATA

     Number of missing data patterns             4
     Number of y missing data patterns           4
     Number of u missing data patterns           0

COVARIANCE COVERAGE OF DATA

Minimum covariance coverage value   0.100

     PROPORTION OF DATA PRESENT FOR Y

           Covariance Coverage
              SLPNSSNC      WORRYNC       SOLC          SLPDURC
              ________      ________      ________      ________
 SLPNSSNC       1.000
 WORRYNC        1.000         1.000
 SOLC           0.976         0.976         0.976
 SLPDURC        0.963         0.963         0.940         0.963

UNIVARIATE SAMPLE STATISTICS

     UNIVARIATE HIGHER-ORDER MOMENT DESCRIPTIVE STATISTICS

         Variable/         Mean/     Skewness/   Minimum/ % with                Percentiles
        Sample Size      Variance    Kurtosis    Maximum  Min/Max      20%/60%    40%/80%    Median

     SLPNSSNC             0.933       0.086       0.000   27.10%       0.000      1.000      1.000
           27802.000       0.471      -0.878       2.000   20.40%       1.000      2.000
     WORRYNC              0.000      -1.036      -2.825    1.71%      -0.928      0.020      0.495
           27802.000       1.000       0.165       0.969   28.55%       0.495      0.969
     SOLC                 0.000       0.967      -1.054   31.63%      -1.054     -0.072     -0.072
           27123.000       1.000       0.467       2.875    2.77%      -0.072      0.910
     SLPDURC              0.000       0.568      -1.205   25.70%      -1.205     -0.292     -0.292
           26763.000       1.000      -0.470       2.445    3.44%      -0.292      0.620

RANDOM STARTS RESULTS RANKED FROM THE BEST TO THE WORST LOGLIKELIHOOD VALUES

380 perturbed starting value run(s) did not converge in the initial stage
optimizations.

Final stage loglikelihood values at local maxima, seeds, and initial stage start numbers:

         -125994.415  691234           250
         -125994.415  246794           801
         -125994.415  917702           693
         -125994.415  970689           266
         -125994.415  244349           736
         -125994.415  478421           311
         -125994.415  278661           674
         -125994.415  930872           277
         -125994.415  618000           190
         -125994.415  49910            829
         -125994.415  314034           513
         -125994.415  91231            727
         -125994.415  851945           18
         -125994.415  926797           406
         -125994.415  791285           416
         -125994.415  544077           809
         -125994.415  268896           124
         -125994.415  27071            15
         -125994.415  177175           851
         -125994.415  485256           371
         -125994.415  495490           990
         -125994.415  349263           263
         -125994.415  3607             873
         -125994.415  168762           200
         -125994.415  109357           765
         -125994.415  544009           842
         -125994.415  180889           920
         -125994.415  636396           168
         -125994.415  772131           407
         -125994.415  383979           603
         -125994.415  373702           669
```

-125994.415	551340	766
-125994.415	303834	798
-125994.415	294811	637
-125994.415	383902	673
-125994.415	462228	298
-125994.415	215353	164
-125994.415	200041	810
-125994.415	130541	896
-125994.415	616917	261
-125994.415	126371	526
-125994.415	849670	347
-125994.415	237332	661
-125994.415	114433	708
-125994.415	903633	553
-125994.415	202790	198
-125994.415	833196	715
-125994.415	137377	397
-125994.415	399508	415
-125994.415	862607	468
-125994.415	544048	87
-125994.415	414284	158
-125994.415	490123	995
-125994.415	937885	426
-125994.415	105435	265
-125994.415	46502	714
-125994.415	992389	77
-125994.415	308582	752
-125994.415	373815	618
-125994.415	856536	144
-125994.415	679448	937
-125994.415	505879	424
-125994.415	224950	455
-125994.415	260953	589
-125994.415	339073	841
-125994.415	575700	100
-125994.415	549244	756
-125994.415	391949	295
-125994.415	701525	239
-125994.415	576596	99
-125994.415	794236	127
-125994.415	484687	306
-125994.415	972430	491
-125994.415	971693	470
-125994.415	453915	975
-125994.415	710154	831
-125994.415	850112	922
-125994.415	443442	380
-125994.415	444228	860
-125994.415	179022	687
-125994.415	50887	389
-125994.415	70118	104
-125994.415	204959	695
-125994.415	570908	98
-125994.415	907810	795
-125994.415	705224	953
-125994.415	963967	941
-125994.415	923437	398
-125994.415	138695	783
-125994.415	124999	96
-125994.415	634782	979
-125994.415	563584	657
-125994.415	742609	531
-125994.415	836515	341
-125994.415	848356	602
-125994.415	298553	773
-125994.415	745972	521
-125994.415	965639	463
-125994.415	264081	186
-125994.415	213532	503
-125994.415	284384	600
-125994.415	673496	218
-125994.415	440368	797
-125994.415	628143	854
-125994.415	847088	750
-125994.415	954914	911
-125994.415	271809	846
-125994.415	679832	302
-125994.415	809240	543
-125994.415	172913	557
-125994.415	830392	35
-125994.415	97300	640
-125994.415	597614	284
-125994.415	723035	538
-125994.415	682718	733
-125994.415	81951	907
-125994.415	879338	309
-125994.415	966499	963
-125994.415	669634	335
-125994.415	863691	481
-125994.415	717754	180
-125994.415	654136	167
-125994.415	193569	440
-125994.415	298201	903
-125994.415	347515	24
-125994.415	545108	667
-125994.415	980970	894
-125994.415	695155	150
-125994.415	766903	505
-125994.415	922596	456
-125994.415	51375	148
-125994.415	158612	581
-125994.415	364676	27
-125994.415	566739	575
-125994.415	442072	625
-125994.415	322790	636
-125994.415	852283	616
-125994.415	677062	680
-125994.415	696830	668
-125994.415	792389	746
-125994.415	535303	923
-125994.415	17896	592
-125994.415	857122	889
-125994.415	735928	916
-125994.415	961454	665
-125994.415	881886	608
-125994.415	370481	742
-125994.415	195353	225
-125994.415	405371	569
-125994.415	415931	10
-125994.415	148918	682
-125994.415	119513	821
-125994.415	303634	169
-125994.415	465160	862
-125994.415	414828	322
-125994.415	333082	578
-125994.415	928624	981
-125994.415	548245	818
-125994.415	599729	658
-125994.415	207896	25
-125994.415	485483	498
-125994.415	640833	434
-125994.415	762461	425
-125994.415	136842	58
-125994.415	311214	64
-125994.415	72662	729
-125994.415	297518	166
-125994.415	61587	400
-125994.415	569833	85
-125994.415	662718	460
-125994.415	484501	163

-125994.415	432513	803
-125994.415	843555	952
-125994.415	unperturbed	0
-125994.415	626208	698
-125994.415	599136	811
-125994.415	721392	768
-125994.415	153394	429
-125994.415	178031	720
-125994.415	728038	177
-125994.415	484406	421
-125994.415	224151	973
-125994.415	589483	950
-125994.415	782821	272
-125994.415	352277	42
-125994.415	238906	430
-125994.415	848331	137
-125994.415	855760	593
-125994.415	891347	504
-125994.415	468036	131
-125994.415	437181	135
-125994.415	552272	654
-125994.415	520177	262
-125994.415	268217	83
-125994.415	580405	286
-125994.415	87586	871
-125994.415	638977	643
-125994.415	55115	408
-125994.415	248742	556
-125994.415	838615	677
-125994.415	751153	110
-125994.415	384199	882
-125994.415	178181	753
-125994.415	22362	365
-125994.415	792993	859
-125994.415	778953	635
-125994.415	286735	175
-125994.415	617243	237
-125994.415	605161	409
-125994.415	846194	93
-125994.415	357866	968
-125994.415	726035	191
-125994.415	483369	270
-125994.415	399380	436
-125994.415	434915	552
-125994.415	782200	84
-125994.415	538872	949
-125994.415	871851	257
-125994.415	814975	129
-125994.415	157351	579
-125994.415	81233	825
-125994.415	526324	178
-125994.415	366706	29
-125994.415	211281	292
-125994.415	848969	173
-125994.415	937588	293
-125994.415	788796	145
-125994.415	574942	558
-125994.415	35191	703
-125994.415	195873	6
-125994.415	118438	601
-125994.415	576726	280
-125994.415	345974	622
-125994.415	467339	66
-125994.415	566687	597
-125994.415	407108	366
-125994.415	791678	974
-125994.415	608460	244
-125994.415	860029	760
-125994.415	14262	781
-125994.415	50983	834
-125994.415	592219	119
-125994.415	891531	206
-125994.415	265218	924
-125994.415	370957	554
-125994.415	264935	281
-125994.415	127362	757
-125994.415	621542	375
-125994.415	326091	759
-125994.415	691041	590

THE BEST LOGLIKELIHOOD VALUE HAS BEEN REPLICATED. RERUN WITH AT LEAST TWICE THE RANDOM STARTS TO CHECK THAT THE BEST LOGLIKELIHOOD IS STILL OBTAINED AND REPLICATED.

THE MODEL ESTIMATION TERMINATED NORMALLY

MODEL FIT INFORMATION

Number of Free Parameters 36

Loglikelihood

H0 Value	-125994.415
H0 Scaling Correction Factor for MLR	1.3296

Information Criteria

Akaike (AIC)	252060.830
Bayesian (BIC)	252357.213
Sample-Size Adjusted BIC (n* = (n + 2) / 24)	252242.806

FINAL CLASS COUNTS AND PROPORTIONS FOR THE LATENT CLASSES
BASED ON THE ESTIMATED MODEL

Latent Classes		
1	5737.65936	0.20638
2	18891.89637	0.67952
3	3172.44427	0.11411

FINAL CLASS COUNTS AND PROPORTIONS FOR THE LATENT CLASSES
BASED ON ESTIMATED POSTERIOR PROBABILITIES

Latent Classes		
1	5737.65936	0.20638
2	18891.89637	0.67952
3	3172.44427	0.11411

FINAL CLASS COUNTS AND PROPORTIONS FOR THE LATENT CLASSES
BASED ON THEIR MOST LIKELY LATENT CLASS MEMBERSHIP

Class Counts and Proportions

Latent Classes		
1	6141	0.22088
2	18658	0.67110
3	3003	0.10801

```
CLASSIFICATION QUALITY

     Entropy                         0.854

Average Latent Class Probabilities for Most Likely Latent
Class Membership (Row)
by Latent Class (Column)

           1        2        3

   1   0.822    0.116    0.063
   2   0.026    0.974    0.000
   3   0.071    0.001    0.928

Classification Probabilities for the Most Likely Latent Class
Membership (Column)
by Latent Class (Row)

           1        2        3

   1   0.879    0.083    0.037
   2   0.038    0.962    0.000
   3   0.122    0.000    0.878


Logits for the Classification Probabilities for the Most Likely
Latent Class Membership (Column)
by Latent Class (Row)

              1        2        3

   1      3.164    0.809    0.000
   2      5.521    8.764    0.000
   3     -1.978  -10.865    0.000

MODEL RESULTS

                                                    Two-Tailed
                    Estimate       S.E.  Est./S.E.    P-Value

Latent Class 1

 SLPNSSNC WITH
    WORRYNC            0.015      0.006      2.633      0.008
    SOLC               0.106      0.008     14.059      0.000
    SLPDURC            0.019      0.009      2.191      0.028

 WORRYNC  WITH
    SOLC               0.088      0.017      5.295      0.000
    SLPDURC           -0.048      0.010     -4.925      0.000

 SOLC     WITH
    SLPDURC            0.051      0.013      3.916      0.000

 Means
    SLPNSSNC           0.931      0.012     78.488      0.000
    WORRYNC           -0.775      0.027    -28.725      0.000
    SOLC               0.353      0.018     19.838      0.000
    SLPDURC            0.391      0.023     17.144      0.000

 Variances
    SLPNSSNC           0.468      0.003    152.809      0.000
    WORRYNC            0.210      0.009     23.619      0.000
    SOLC               0.626      0.013     47.707      0.000
    SLPDURC            0.798      0.010     76.790      0.000

Latent Class 2

 SLPNSSNC WITH
    WORRYNC           -0.030      0.004     -8.232      0.000
    SOLC               0.118      0.005     22.000      0.000
    SLPDURC            0.037      0.006      6.407      0.000

 WORRYNC  WITH
    SOLC              -0.192      0.008    -25.245      0.000
    SLPDURC           -0.187      0.010    -18.506      0.000

 SOLC     WITH
    SLPDURC            0.290      0.011     27.050      0.000

 Means
    SLPNSSNC           0.904      0.005    171.611      0.000
    WORRYNC            0.570      0.006    101.171      0.000
    SOLC              -0.364      0.009    -40.558      0.000
    SLPDURC           -0.300      0.007    -40.716      0.000

 Variances
    SLPNSSNC           0.468      0.003    152.809      0.000
    WORRYNC            0.210      0.009     23.619      0.000
    SOLC               0.626      0.013     47.707      0.000
    SLPDURC            0.798      0.010     76.790      0.000

Latent Class 3

 SLPNSSNC WITH
    WORRYNC            0.009      0.006      1.533      0.125
    SOLC               0.046      0.011      4.198      0.000
    SLPDURC            0.013      0.012      1.153      0.249

 WORRYNC  WITH
    SOLC              -0.006      0.006     -0.926      0.354
    SLPDURC           -0.070      0.009     -7.748      0.000

 SOLC     WITH
    SLPDURC            0.050      0.015      3.310      0.001

 Means
    SLPNSSNC           1.112      0.015     76.093      0.000
    WORRYNC           -1.994      0.027    -74.945      0.000
    SOLC               1.572      0.036     43.121      0.000
    SLPDURC            1.108      0.027     40.596      0.000

 Variances
    SLPNSSNC           0.468      0.003    152.809      0.000
    WORRYNC            0.210      0.009     23.619      0.000
    SOLC               0.626      0.013     47.707      0.000
    SLPDURC            0.798      0.010     76.790      0.000

Categorical Latent Variables

 Means
    C#1                0.593      0.046     12.891      0.000
    C#2                1.784      0.040     44.896      0.000

QUALITY OF NUMERICAL RESULTS

     Condition Number for the Information Matrix
0.903E-04
       (ratio of smallest to largest eigenvalue)

TECHNICAL 11 OUTPUT

     Random Starts Specifications for the k-1 Class Analysis
Model
        Number of initial stage random starts                  1000
        Number of final stage optimizations                     250

     VUONG-LO-MENDELL-RUBIN LIKELIHOOD RATIO
TEST FOR 2 (H0) VERSUS 3 CLASSES
```

```
          H0 Loglikelihood Value                 -127763.908
          2 Times the Loglikelihood Difference          3538.985
          Difference in the Number of Parameters              11
          Mean                                  61.969
          Standard Deviation                           60.777
          P-Value                                0.0000

     LO-MENDELL-RUBIN ADJUSTED LRT TEST

          Value                                3507.822
          P-Value                                0.0000

TECHNICAL 14 OUTPUT

     Random Starts Specifications for the k-1 Class Analysis
Model
        Number of initial stage random starts                1000
        Number of final stage optimizations                   250

     Random Starts Specification for the k-1 Class Model for
Generated Data
        Number of initial stage random starts                   2
        Number of final stage optimizations                     1
     Random Starts Specification for the k Class Model for
Generated Data
        Number of initial stage random starts                  50
        Number of final stage optimizations                    10
     Number of bootstrap draws requested                      250

     PARAMETRIC BOOTSTRAPPED LIKELIHOOD RATIO
TEST FOR 2 (H0) VERSUS 3 CLASSES

          H0 Loglikelihood Value                 -127763.908
          2 Times the Loglikelihood Difference          3538.985
          Difference in the Number of Parameters              11
          Approximate P-Value                        0.0000
          Successful Bootstrap Draws                       250

     WARNING:  OF THE 250 BOOTSTRAP DRAWS, 230
DRAWS HAD BOTH A SMALLER LRT VALUE THAN THE
     OBSERVED LRT VALUE AND NOT A REPLICATED
BEST LOGLIKELIHOOD VALUE FOR THE 3-CLASS
MODEL.
     THIS MEANS THAT THE P-VALUE MAY NOT BE
TRUSTWORTHY DUE TO LOCAL MAXIMA.
     INCREASE THE NUMBER OF RANDOM STARTS
USING THE LRTSTARTS OPTION.

PLOT INFORMATION

The following plots are available:

  Histograms (sample values)
  Scatterplots (sample values)
  Sample means
  Estimated means, medians, modes, and percentiles
  Sample and estimated means
  Observed individual values
  Estimated means and observed individual values
  Estimated overall and class-specific distributions

SAVEDATA INFORMATION

  Save file
    Step1_LPA3M5.dat

  Order and format of variables

    SLPNSSNC       F10.3
    WORRYNC        F10.3
    SOLC           F10.3
    SLPDURC        F10.3
    CPROB1         F10.3
    CPROB2         F10.3
    CPROB3         F10.3
    C              F10.3
    ID             I6

  Save file format
    8F10.3 I6

  Save file record length    10000

  Save missing symbol        *

DIAGRAM INFORMATION

  Mplus diagrams are currently not available for Mixture
analysis.
  No diagram output was produced.

     Beginning Time:  09:24:01
        Ending Time:  11:07:04
       Elapsed Time:  01:43:03

MUTHEN & MUTHEN
3463 Stoner Ave.
Los Angeles, CA  90066

Tel: (310) 391-9971
Fax: (310) 391-8971
Web: www.StatModel.com
Support: Support@StatModel.com

Copyright (c) 1998-2021 Muthen & Muthen
```

Four-profile model

```
Mplus VERSION 8.7
MUTHEN & MUTHEN
07/25/2023   1:46 PM

INPUT INSTRUCTIONS

  TITLE: LPA 4 profile syntax: M5: Varying means, equal
variances, and varying covariances

  DATA:
  FILE IS mplus2.csv;
  ! Specifies file location for data file. Make sure data is in
format appropriate for Mplus
  ! per Mplus manual. This data file is in individual format
(one row of data per participan
  VARIABLE:
  NAMES ARE ID SCI_02 SCI_02B slpnssN worryN sol
slpDur gender genderN yrgrp yrgrpN SCI_02C
  slpnssNC worryNC solC slpDurC;

  ! All variables included in data file should be named here.
  USEVARIABLES ARE slpnssNC worryNC solC slpDurC;
  ! Only variables intended for use in the analysis should be
listed here
  IDVARIABLE IS ID;
  CLASSES = c (4);
```

```
  ! This is where you instruct Mplus on how many
classes/profiles are being estimated. Initi
  ! model contains only one class/profile, thus it would be
CLASSES = c (1). Above specifies
  !profiles, and for each further iterative models the number
in parentheses increases by on
  !three profiles/classes would be c (3), and so on.
  MISSING ARE ALL(999999);
  ! Used to communicate how missing data is coded in data
file. Here shown with a "." which
  ! all that is included in each cell with missing data in the
data file
  ANALYSIS:
  TYPE = MIXTURE;
  ! LPA is a version of mixture modeling, and this instructs
Mplus to analyze in this way
  ESTIMATOR = MLR;
  !FIML robust to non-normal data
  STARTS = 1000 250;
  STITERATIONS = 500;
  ! Default number of starts for each step of the ML
estimation. First STARTS value specifie
  !number of unique start values to start with, the 250
represents the 250 best unique start
  !carrying forward to completion. The STITERATIONS
specifies the number of ML iteration
  !steps for those 250 selected start values to go through to
be able to converge. This is a
  !maximum number of iteration; if a model converges in less
than 500 iterations it will sto
  !before reaching 500 iterations.
  !These values can be increased … see "Four-Profile Final
Model with Covariate Analysis
  !Syntax" for an example.
  LRTSTARTS = 2 1 50 10;
  LRTBOOTSTRAP = 250;
  !The above start values are for the defaults for the LRT
statistic being run to compare th
  !model fit with the model fit of a model with one less class
(k-1). The BOOTSTRAP statemen
  !specifies the number of bootstrap draws to inform Mplus'
bootstrapped LRT results.
  MODEL:
  !For a default Mplus model the LPA model does not need
to be specified. However, it can be
  !The model can also be modified from the Mplus default of
estimating the indicator means
  !(uniquely across profiles) and variances (constrained
across profiles), as well as the la
  !profile mean.
  %OVERALL%
  [slpnssNC worryNC solC slpDurC]; !estimates the
indicators means for each profile. Without
  !the means are freely estimated in each profile, not
constrained.
  slpnssNC worryNC solC slpDurC; !Label Var1-Var5
constrains the estimates of the variances
  !profiles to be equal.

  %c#1%

      [slpnssNC worryNC solC slpDurC];

      !covariances
  slpnssNC WITH worryNC solC slpDurC;
  worryNC WITH solC slpDurC;
  solC WITH slpDurC;

    %c#2%

      [slpnssNC worryNC solC slpDurC];

      !covariances
  slpnssNC WITH worryNC solC slpDurC;
  worryNC WITH solC slpDurC;
  solC WITH slpDurC;

  %c#3%

      [slpnssNC worryNC solC slpDurC];

      !covariances
  slpnssNC WITH worryNC solC slpDurC;
  worryNC WITH solC slpDurC;
  solC WITH slpDurC;

    %c#4%

      [slpnssNC worryNC solC slpDurC];

      !covariances
  slpnssNC WITH worryNC solC slpDurC;
  worryNC WITH solC slpDurC;
  solC WITH slpDurC;

  OUTPUT:
  TECH11 TECH14;
  ! TECH1 provides parameter specifications and starting
values for the analysis
  ! TECH8 provides optimization history for this analysis type
  !TECH11 provides LRT results
  !TECH14 provides bootstrapped LRT test
  PLOT: SERIES=slpnssNC worryNC solC slpDurC(*);
  TYPE=PLOT3;
  SAVEDATA:
  FILE IS Step2_LPA4M5.dat;
  ! Tells Mplus where to save the output files from the
analysis
  SAVE = CPROBABILITIES;
  ! The above command lines are to save the most likely
profile membership for each particip
  ! and the posterior probabilities for their membership in
each latent profile.

*** WARNING in MODEL command
  All variables are uncorrelated with all other variables within
class.
  Check that this is what is intended.

LPA 4 profile syntax: M5: Varying means, equal variances,
and varying covariances

SUMMARY OF ANALYSIS

Number of groups                                    1
Number of observations                          27802

Number of dependent variables                       4
Number of independent variables                     0
Number of continuous latent variables               0
Number of categorical latent variables              1

Observed dependent variables

  Continuous
   SLPNSSNC    WORRYNC     SOLC       SLPDURC

Categorical latent variables
   C
```

```
Variables with special functions

  ID variable          ID

Estimator                                          MLR
Information matrix                                 OBSERVED
Optimization Specifications for the Quasi-Newton Algorithm
for
Continuous Outcomes
  Maximum number of iterations                     100
  Convergence criterion                            0.100D-05
Optimization Specifications for the EM Algorithm
  Maximum number of iterations                     500
  Convergence criteria
    Loglikelihood change                           0.100D-06
    Relative loglikelihood change                  0.100D-06
    Derivative                                     0.100D-05
Optimization Specifications for the M step of the EM
Algorithm for
Categorical Latent variables
  Number of M step iterations                      1
  M step convergence criterion                     0.100D-05
  Basis for M step termination                     ITERATION
Optimization Specifications for the M step of the EM
Algorithm for
Censored, Binary or Ordered Categorical (Ordinal),
Unordered
Categorical (Nominal) and Count Outcomes
  Number of M step iterations                      1
  M step convergence criterion                     0.100D-05
  Basis for M step termination                     ITERATION
  Maximum value for logit thresholds               15
  Minimum value for logit thresholds               -15
  Minimum expected cell size for chi-square        0.100D-
01
Maximum number of iterations for H1                2000
Convergence criterion for H1                       0.100D-03
Optimization algorithm                             EMA
Random Starts Specifications
  Number of initial stage random starts            1000
  Number of final stage optimizations              250
  Number of initial stage iterations               500
  Initial stage convergence criterion              0.100D+01
  Random starts scale                              0.500D+01
  Random seed for generating random starts         0

Input data file(s)
  mplus2.csv
Input data format  FREE

SUMMARY OF DATA

    Number of missing data patterns          4
    Number of y missing data patterns        4
    Number of u missing data patterns        0

COVARIANCE COVERAGE OF DATA

Minimum covariance coverage value   0.100

    PROPORTION OF DATA PRESENT FOR Y

        Covariance Coverage
          SLPNSSNC      WORRYNC       SOLC
SLPDURC
          ________      ________      ________      ________
 SLPNSSNC       1.000
 WORRYNC        1.000         1.000
 SOLC           0.976         0.976         0.976
 SLPDURC        0.963         0.963         0.940         0.963

UNIVARIATE SAMPLE STATISTICS

    UNIVARIATE HIGHER-ORDER MOMENT
DESCRIPTIVE STATISTICS

     Variable/       Mean/     Skewness/   Minimum/ % with
Percentiles
    Sample Size     Variance    Kurtosis    Maximum
Min/Max     20%/60%    40%/80%    Median

    SLPNSSNC          0.933       0.086       0.000   27.10%
0.000      1.000      1.000
         27802.000      0.471      -0.878       2.000   20.40%
1.000      2.000
    WORRYNC           0.000      -1.036      -2.825    1.71%
-0.928     0.020      0.495
         27802.000      1.000       0.165       0.969   28.55%
0.495      0.969
    SOLC              0.000       0.967      -1.054   31.63%      -
1.054     -0.072     -0.072
         27123.000      1.000       0.467       2.875    2.77%
-0.072     0.910
    SLPDURC           0.000       0.568      -1.205   25.70%
-1.205     -0.292     -0.292
         26763.000      1.000      -0.470       2.445    3.44%
-0.292     0.620

RANDOM STARTS RESULTS RANKED FROM THE BEST
TO THE WORST LOGLIKELIHOOD VALUES

Unperturbed starting value run did not converge in the initial
stage
optimizations.

696 perturbed starting value run(s) did not converge in the
initial stage
optimizations.

Final stage loglikelihood values at local maxima, seeds, and
initial stage start numbers:

       -124709.900  182524           373
       -124709.900  42523            730
       -124709.900  512836           289
       -124709.900  373702           669
       -124709.900  497522           502
       -124709.900  299977           956
       -124709.900  298201           903
       -124709.900  944186           541
       -124709.900  319144           176
       -124709.900  402224           91
       -124709.900  73576            213
       -124709.900  863691           481
       -124709.900  246261           38
       -124709.900  790452           303
       -124709.900  252346           528
       -124709.900  407108           366
       -124709.900  461687           833
       -124709.900  798821           423
       -124709.900  997222           229
       -124709.900  782179           835
       -124709.900  848969           173
       -124709.900  739214           807
       -124709.900  317868           740
       -124709.900  415931           10
       -124709.900  190339           102
       -124709.900  781489           627
       -124709.900  875667           367
       -124709.900  685657           69
```

-124709.900	383986	159
-124709.900	566739	575
-124709.900	579995	183
-124709.900	443917	60
-124709.900	266038	672
-124709.900	301428	885
-124709.900	384199	882
-124709.900	50887	389
-124709.900	724519	333
-124709.900	853195	431
-124709.900	165853	105
-124709.900	464179	106
-124709.900	699554	427
-124709.900	207896	25
-124709.900	637095	207
-124709.900	679448	937
-124709.900	87586	871
-124709.900	529455	268
-124709.900	779820	877
-124709.900	769907	457
-124709.900	535303	923
-124709.900	574412	679
-124709.900	3307	735
-124709.900	276102	599
-124709.900	486622	522
-124709.900	349360	464
-124709.900	603842	61
-124709.900	575700	100
-124709.900	232559	136
-124709.900	481835	57
-124709.900	544009	842
-124709.900	928624	981
-124709.900	547702	767
-124709.900	59963	670
-124709.900	246794	801
-124709.900	291112	645
-124709.900	728038	177
-124709.900	357866	968
-124709.900	197223	332
-124709.900	741888	138
-124709.900	192191	629
-124709.900	780698	337
-124709.900	891347	504
-124709.900	349263	263
-124709.900	576783	866
-124709.900	81951	907
-124709.900	879338	309
-124709.900	499150	216
-124709.900	204959	695
-124709.900	3607	873
-124709.900	607072	717
-124709.900	321390	133
-124709.900	84013	598
-124709.900	814975	129
-124709.900	640833	434
-124709.900	375590	438
-124709.900	872743	374
-124709.900	140442	500
-124709.900	315029	471
-124709.900	467339	66
-124709.900	59674	240
-124709.900	473343	844
-124709.900	526324	178
-124709.900	424223	900
-124709.900	11397	944
-124709.900	404042	675
-124709.900	391949	295
-124709.900	937588	293
-124709.900	648035	836
-124709.900	496703	758
-124709.900	484406	421
-124709.900	97300	640
-124709.900	966499	963
-124709.900	584397	428
-124709.900	746978	410
-124709.900	259507	53
-124709.900	876056	1000
-124709.900	965994	396
-124709.900	173191	422
-124709.900	406935	958
-124709.900	266340	683
-124709.900	604861	800
-124709.900	471398	74
-124709.900	644297	340
-124709.900	804104	566
-124709.900	726035	191
-124709.900	907810	795
-124709.900	195873	6
-124709.900	694303	282
-124709.900	284384	600
-124709.900	544048	87
-124709.900	153394	429
-124709.900	563584	657
-124709.900	847088	750
-124709.900	954354	840
-124709.900	688839	273
-124709.900	950604	172
-124709.900	436806	883
-124709.900	576726	280
-124709.900	539389	544
-124709.900	673496	218
-124709.900	552272	654
-124709.900	561664	392
-124709.900	66276	217
-124709.900	371246	101
-124709.900	647617	725
-124709.900	177936	726
-124709.900	549244	756
-124709.900	227563	63
-124709.900	392418	28
-124709.900	49293	707
-124709.900	392407	221
-124709.900	94610	884
-124709.900	931558	898
-124709.900	178181	753
-124709.900	501995	791
-124709.900	158612	581
-124709.900	833196	715
-124709.900	995648	936
-124709.900	858804	534
-124709.900	364676	27
-124709.900	602032	648
-124709.900	850112	922
-124709.900	869564	869
-124709.900	948615	140
-124709.900	76974	16
-124709.900	427006	839
-124709.900	508482	446
-124709.900	263268	165
-124709.900	46437	153
-124709.900	758647	951
-124709.900	414284	158
-124709.900	118421	139
-124709.900	995875	547
-124709.900	188498	258
-124709.900	545108	667
-124709.900	645052	910
-124709.900	862607	468
-124709.900	840031	276
-124709.900	569833	85

```
-124709.900  863094    147
-124709.900  506886    576
-124709.900  638611    524
-124709.900  271809    846
-124709.900  244349    736
-124709.900  597614    284
-124709.900  579138    706
-124709.900  455617    242
-124709.900  852283    616
-124709.900  193042    316
-124709.900  370466    41
-124709.900  23012     352
-124709.900  407168    44
-124709.900  178031    720
-124709.900  789985    67
-124709.900  509733    130
-124709.900  339073    841
-124709.900  804660    260
-124709.900  947714    573
-124709.900  692169    308
-124709.900  922596    456
-124709.900  53621     483
-124709.900  505244    582
-124709.900  915107    54
-124709.900  638577    769
-124709.900  715255    523
-124709.900  475419    987
-124709.900  125727    899
-124709.900  954914    911
-124709.900  442072    625
-124709.900  851945    18
-124709.900  978781    497
-124709.900  432148    30
-124709.900  297518    166
-124709.900  177175    851
-124709.900  376411    473
-124709.900  650371    14
-124709.900  215353    164
-124709.900  930872    277
-124709.900  478421    311
-124709.900  766903    505
-124709.900  301180    236
-124709.900  848163    47
-124709.900  326091    759
-124709.900  700846    867
-124709.900  39136     226
-124709.900  306768    978
-124709.900  466971    109
-124709.900  292884    103
-124709.900  926283    269
-124709.900  629320    222
-124709.900  871438    561
-124709.900  574942    558
-124709.900  311214    64
-124709.900  440395    917
-124709.900  843555    952
-124709.900  626891    32
-124709.900  403801    762
-124709.900  677720    681
-124709.900  717754    180
-124709.900  655497    376
-124709.900  387701    275
-124709.900  391368    802
-124709.900  798839    312
-124709.900  996231    310
-124709.900  636396    168
-124709.900  193847    354
-124709.900  393232    152
-124709.900  61587     400
-124709.900  98068     998
-124709.900  399848    220
-124709.900  961454    665
-124709.900  314757    345
-124709.900  618760    489
-124709.900  760878    249
-124709.900  172913    557
-124709.900  345726    461
-124709.900  638977    643
-124709.900  856536    144
-124709.900  860772    174
-124709.900  781190    219
-124709.900  967902    52

THE BEST LOGLIKELIHOOD VALUE HAS BEEN
REPLICATED.  RERUN WITH AT LEAST TWICE THE
RANDOM STARTS TO CHECK THAT THE BEST
LOGLIKELIHOOD IS STILL OBTAINED AND
REPLICATED.

THE MODEL ESTIMATION TERMINATED NORMALLY

MODEL FIT INFORMATION

Number of Free Parameters                    47

Loglikelihood

          H0 Value                    -124709.900
          H0 Scaling Correction Factor      1.1775
            for MLR

Information Criteria

          Akaike (AIC)                249513.801
          Bayesian (BIC)              249900.745
          Sample-Size Adjusted BIC      249751.380
            (n* = (n + 2) / 24)

FINAL CLASS COUNTS AND PROPORTIONS FOR THE
LATENT CLASSES
BASED ON THE ESTIMATED MODEL

    Latent
   Classes

       1       4456.90910          0.16031
       2       2657.87540          0.09560
       3       1910.44951          0.06872
       4      18776.76599          0.67537


FINAL CLASS COUNTS AND PROPORTIONS FOR THE
LATENT CLASSES
BASED ON ESTIMATED POSTERIOR PROBABILITIES

    Latent
   Classes

       1       4456.90910          0.16031
       2       2657.87540          0.09560
       3       1910.44951          0.06872
       4      18776.76599          0.67537

FINAL CLASS COUNTS AND PROPORTIONS FOR THE
LATENT CLASSES
BASED ON THEIR MOST LIKELY LATENT CLASS
MEMBERSHIP

Class Counts and Proportions
```

```
  Latent
 Classes

    1         4162          0.14970
    2         2502          0.08999
    3         1451          0.05219
    4        19687          0.70811

CLASSIFICATION QUALITY

     Entropy                         0.855

Average Latent Class Probabilities for Most Likely Latent
Class Membership (Row)
by Latent Class (Column)

           1        2        3        4

    1   0.862    0.055    0.011    0.072
    2   0.071    0.912    0.016    0.001
    3   0.023    0.097    0.797    0.083
    4   0.034    0.000    0.034    0.932

Classification Probabilities for the Most Likely Latent Class
Membership (Column)
by Latent Class (Row)

           1        2        3        4

    1   0.805    0.040    0.007    0.148
    2   0.087    0.859    0.053    0.001
    3   0.025    0.021    0.605    0.350
    4   0.016    0.000    0.006    0.978

Logits for the Classification Probabilities for the Most Likely
Latent Class Membership (Column)
by Latent Class (Row)

              1        2        3        4

    1     1.691   -1.315   -3.000    0.000
    2     4.229    6.523    3.740    0.000
    3    -2.648   -2.830    0.548    0.000
    4    -4.119   -8.984   -5.021    0.000

MODEL RESULTS

                                                Two-Tailed
                    Estimate       S.E.  Est./S.E.    P-Value

Latent Class 1

 SLPNSSNC WITH
    WORRYNC           -0.002      0.006     -0.280      0.780
    SOLC               0.081      0.007     10.882      0.000
    SLPDURC            0.030      0.009      3.201      0.001

 WORRYNC  WITH
    SOLC              -0.007      0.005     -1.446      0.148
    SLPDURC           -0.076      0.009     -8.952      0.000

 SOLC     WITH
    SLPDURC            0.063      0.010      6.438      0.000

 Means
    SLPNSSNC           0.872      0.014     64.494      0.000
    WORRYNC           -1.107      0.021    -52.403      0.000
    SOLC               0.029      0.017      1.766      0.077
    SLPDURC            0.455      0.022     21.037      0.000

 Variances
    SLPNSSNC           0.460      0.003    150.050      0.000
    WORRYNC            0.245      0.004     55.585      0.000
    SOLC               0.405      0.006     72.186      0.000
    SLPDURC            0.797      0.008     98.354      0.000

Latent Class 2

 SLPNSSNC WITH
    WORRYNC            0.003      0.007      0.398      0.691
    SOLC               0.007      0.011      0.686      0.492
    SLPDURC            0.012      0.012      0.968      0.333

 WORRYNC  WITH
    SOLC              -0.042      0.008     -5.604      0.000
    SLPDURC           -0.089      0.010     -8.966      0.000

 SOLC     WITH
    SLPDURC            0.029      0.013      2.263      0.024

 Means
    SLPNSSNC           1.148      0.015     75.059      0.000
    WORRYNC           -1.962      0.022    -88.615      0.000
    SOLC               1.874      0.022     85.791      0.000
    SLPDURC            1.137      0.025     46.280      0.000

 Variances
    SLPNSSNC           0.460      0.003    150.050      0.000
    WORRYNC            0.245      0.004     55.585      0.000
    SOLC               0.405      0.006     72.186      0.000
    SLPDURC            0.797      0.008     98.354      0.000

Latent Class 3

 SLPNSSNC WITH
    WORRYNC           -0.019      0.010     -1.946      0.052
    SOLC               0.010      0.015      0.653      0.514
    SLPDURC           -0.022      0.017     -1.290      0.197

 WORRYNC  WITH
    SOLC              -0.075      0.012     -6.420      0.000
    SLPDURC           -0.102      0.014     -7.163      0.000

 SOLC     WITH
    SLPDURC            0.049      0.020      2.429      0.015

 Means
    SLPNSSNC           1.189      0.021     56.070      0.000
    WORRYNC            0.017      0.049      0.341      0.733
    SOLC               1.520      0.047     32.307      0.000
    SLPDURC            0.303      0.047      6.470      0.000

 Variances
    SLPNSSNC           0.460      0.003    150.050      0.000
    WORRYNC            0.245      0.004     55.585      0.000
    SOLC               0.405      0.006     72.186      0.000
    SLPDURC            0.797      0.008     98.354      0.000

Latent Class 4

 SLPNSSNC WITH
    WORRYNC           -0.022      0.004     -6.262      0.000
    SOLC               0.075      0.004     18.458      0.000
    SLPDURC            0.022      0.005      4.056      0.000

 WORRYNC  WITH
    SOLC              -0.170      0.005    -37.049      0.000
    SLPDURC           -0.207      0.006    -33.958      0.000

 SOLC     WITH
```

```
    SLPDURC          0.217      0.007     33.011      0.000

  Means
    SLPNSSNC         0.891      0.005    164.073      0.000
    WORRYNC          0.539      0.005     99.557      0.000
    SOLC            -0.420      0.008    -54.829      0.000
    SLPDURC         -0.294      0.007    -41.098      0.000

  Variances
    SLPNSSNC         0.460      0.003    150.050      0.000
    WORRYNC          0.245      0.004     55.585      0.000
    SOLC             0.405      0.006     72.186      0.000
    SLPDURC          0.797      0.008     98.354      0.000

Categorical Latent Variables

  Means
    C#1             -1.438      0.027    -54.013      0.000
    C#2             -1.955      0.033    -60.126      0.000
    C#3             -2.285      0.055    -41.873      0.000

QUALITY OF NUMERICAL RESULTS

     Condition Number for the Information Matrix
0.201E-04
       (ratio of smallest to largest eigenvalue)

TECHNICAL 11 OUTPUT

     Random Starts Specifications for the k-1 Class Analysis
Model
        Number of initial stage random starts                1000
        Number of final stage optimizations                   250

     VUONG-LO-MENDELL-RUBIN LIKELIHOOD RATIO
TEST FOR 3 (H0) VERSUS 4 CLASSES

          H0 Loglikelihood Value                  -125994.415
          2 Times the Loglikelihood Difference       2569.029
          Difference in the Number of Parameters           11
          Mean                                        -20.016
          Standard Deviation                           62.940
          P-Value                                      0.0000

     LO-MENDELL-RUBIN ADJUSTED LRT TEST

          Value                                      2546.407
          P-Value                                      0.0000

TECHNICAL 14 OUTPUT

     Random Starts Specifications for the k-1 Class Analysis
Model
        Number of initial stage random starts                1000
        Number of final stage optimizations                   250

     Random Starts Specification for the k-1 Class Model for
Generated Data
        Number of initial stage random starts                   2
        Number of final stage optimizations                     1
     Random Starts Specification for the k Class Model for
Generated Data
        Number of initial stage random starts                  50
        Number of final stage optimizations                    10
     Number of bootstrap draws requested                      250

     PARAMETRIC BOOTSTRAPPED LIKELIHOOD RATIO
TEST FOR 3 (H0) VERSUS 4 CLASSES

          H0 Loglikelihood Value                  -125994.415
          2 Times the Loglikelihood Difference       2569.029
          Difference in the Number of Parameters           11
          Approximate P-Value                          0.0000
          Successful Bootstrap Draws                      250

     WARNING:  OF THE 250 BOOTSTRAP DRAWS, 233
DRAWS HAD BOTH A SMALLER LRT VALUE THAN THE
     OBSERVED LRT VALUE AND NOT A REPLICATED
BEST LOGLIKELIHOOD VALUE FOR THE 4-CLASS
MODEL.
     THIS MEANS THAT THE P-VALUE MAY NOT BE
TRUSTWORTHY DUE TO LOCAL MAXIMA.
     INCREASE THE NUMBER OF RANDOM STARTS
USING THE LRTSTARTS OPTION.

PLOT INFORMATION

The following plots are available:

  Histograms (sample values)
  Scatterplots (sample values)
  Sample means
  Estimated means, medians, modes, and percentiles
  Sample and estimated means
  Observed individual values
  Estimated means and observed individual values
  Estimated overall and class-specific distributions

SAVEDATA INFORMATION

  Save file
    Step2_LPA4M5.dat

  Order and format of variables

    SLPNSSNC       F10.3
    WORRYNC        F10.3
    SOLC           F10.3
    SLPDURC        F10.3
    CPROB1         F10.3
    CPROB2         F10.3
    CPROB3         F10.3
    CPROB4         F10.3
    C              F10.3
    ID             I6

  Save file format
    9F10.3 I6

  Save file record length    10000

  Save missing symbol        *

DIAGRAM INFORMATION

  Mplus diagrams are currently not available for Mixture
analysis.
  No diagram output was produced.

     Beginning Time:  13:46:02
        Ending Time:  16:52:39
       Elapsed Time:  03:06:37

MUTHEN & MUTHEN
3463 Stoner Ave.
Los Angeles, CA  90066

Tel: (310) 391-9971
Fax: (310) 391-8971
Web: www.StatModel.com
```

Support: Support@StatModel.com	Copyright (c) 1998-2021 Muthen & Muthen

Five-profile model

```
Mplus VERSION 8.7
MUTHEN & MUTHEN
07/13/2023   8:14 PM

INPUT INSTRUCTIONS

  TITLE: LPA 5 profile syntax: M5: Varying means, equal
variances, and varying covariances

  DATA:
  FILE IS mplus2.csv;
  ! Specifies file location for data file. Make sure data is in
format appropriate for Mplus
  ! per Mplus manual. This data file is in individual format
(one row of data per participan
  VARIABLE:
  NAMES ARE ID SCI_02 SCI_02B slpnssN worryN sol
slpDur gender genderN yrgrp yrgrpN SCI_02C
  slpnssNC worryNC solC slpDurC;

  ! All variables included in data file should be named here.
  USEVARIABLES ARE slpnssNC worryNC solC slpDurC;
  ! Only variables intended for use in the analysis should be
listed here
  IDVARIABLE IS ID;
  CLASSES = c (5);
  ! This is where you instruct Mplus on how many
classes/profiles are being estimated. Initi
  ! model contains only one class/profile, thus it would be
CLASSES = c (1). Above specifies
  !profiles, and for each further iterative models the number
in parentheses increases by on
  !three profiles/classes would be c (3), and so on.
  MISSING ARE ALL(9999);
  ! Used to communicate how missing data is coded in data
file. Here shown with a "." which
  ! all that is included in each cell with missing data in the
data file
  ANALYSIS:
  TYPE = MIXTURE;
  ! LPA is a version of mixture modeling, and this instructs
Mplus to analyze in this way
  ESTIMATOR = MLR;
  !FIML robust to non-normal data
  STARTS = 2000 500;
  STITERATIONS = 500;
  ! Default number of starts for each step of the ML
estimation. First STARTS value specifie
  !number of unique start values to start with, the 250
represents the 250 best unique start
  !carrying forward to completion. The STITERATIONS
specifies the number of ML iteration
  !steps for those 250 selected start values to go through to
be able to converge. This is a
  !maximum number of iteration; if a model converges in less
than 500 iterations it will sto
  !before reaching 500 iterations.
  !These values can be increased … see "Four-Profile Final
Model with Covariate Analysis
  !Syntax" for an example.
  LRTSTARTS = 4 2 100 20;
  LRTBOOTSTRAP = 500;
  !The above start values are for the defaults for the LRT
statistic being run to compare th
  !model fit with the model fit of a model with one less class
(k-1). The BOOTSTRAP statemen
  !specifies the number of bootstrap draws to inform Mplus'
bootstrapped LRT results.
  MODEL:
  !For a default Mplus model the LPA model does not need
to be specified. However, it can be
  !The model can also be modified from the Mplus default of
estimating the indicator means
  !(uniquely across profiles) and variances (constrained
across profiles), as well as the la
  !profile mean.
  %OVERALL%
  [slpnssNC worryNC solC slpDurC]; !estimates the
indicators means for each profile. Without
  !the means are freely estimated in each profile, not
constrained.
  slpnssNC worryNC solC slpDurC; !Label Var1-Var5
constrains the estimates of the variances
  !profiles to be equal.

  %c#1%

    [slpnssNC worryNC solC slpDurC];

    !covariances
  slpnssNC WITH worryNC solC slpDurC;
  worryNC WITH solC slpDurC;
  solC WITH slpDurC;

   %c#2%

    [slpnssNC worryNC solC slpDurC];

    !covariances
  slpnssNC WITH worryNC solC slpDurC;
  worryNC WITH solC slpDurC;
  solC WITH slpDurC;

  %c#3%

    [slpnssNC worryNC solC slpDurC];

    !covariances
  slpnssNC WITH worryNC solC slpDurC;
  worryNC WITH solC slpDurC;
  solC WITH slpDurC;

   %c#4%

    [slpnssNC worryNC solC slpDurC];

    !covariances
  slpnssNC WITH worryNC solC slpDurC;
  worryNC WITH solC slpDurC;
  solC WITH slpDurC;

   %c#5%

    [slpnssNC worryNC solC slpDurC];

    !covariances
  slpnssNC WITH worryNC solC slpDurC;
  worryNC WITH solC slpDurC;
```

```
  solC WITH slpDurC;

  OUTPUT:
  TECH11 TECH14;
  ! TECH1 provides parameter specifications and starting values for the analysis
  ! TECH8 provides optimization history for this analysis type
  !TECH11 provides LRT results
  !TECH14 provides bootstrapped LRT test
  PLOT: SERIES=slpnssNC worryNC solC slpDurC(*);
  TYPE=PLOT3;
  SAVEDATA:
  FILE IS Step2_LPA5M5.dat;
  ! Tells Mplus where to save the output files from the analysis
  SAVE = CPROBABILITIES;
  ! The above command lines are to save the most likely profile membership for each particip
  ! and the posterior probabilities for their membership in each latent profile.

*** WARNING in MODEL command
  All variables are uncorrelated with all other variables within class.
  Check that this is what is intended.

LPA 5 profile syntax: M5: Varying means, equal variances, and varying covariances

SUMMARY OF ANALYSIS

Number of groups                                           1
Number of observations                                 27802

Number of dependent variables                              4
Number of independent variables                            0
Number of continuous latent variables                      0
Number of categorical latent variables                     1

Observed dependent variables

  Continuous
   SLPNSSNC    WORRYNC     SOLC        SLPDURC

Categorical latent variables
   C

Variables with special functions

  ID variable           ID

Estimator                                                MLR
Information matrix                                  OBSERVED
Optimization Specifications for the Quasi-Newton Algorithm for
Continuous Outcomes
  Maximum number of iterations                           100
  Convergence criterion                            0.100D-05
Optimization Specifications for the EM Algorithm
  Maximum number of iterations                           500
  Convergence criteria
    Loglikelihood change                           0.100D-06
    Relative loglikelihood change                  0.100D-06
    Derivative                                     0.100D-05
Optimization Specifications for the M step of the EM Algorithm for
Categorical Latent variables
  Number of M step iterations                              1
  M step convergence criterion                     0.100D-05
  Basis for M step termination                     ITERATION
Optimization Specifications for the M step of the EM Algorithm for
Censored, Binary or Ordered Categorical (Ordinal), Unordered
Categorical (Nominal) and Count Outcomes
  Number of M step iterations                              1
  M step convergence criterion                     0.100D-05
  Basis for M step termination                     ITERATION
  Maximum value for logit thresholds                      15
  Minimum value for logit thresholds                     -15
  Minimum expected cell size for chi-square        0.100D-01
Maximum number of iterations for H1                     2000
Convergence criterion for H1                       0.100D-03
Optimization algorithm                                   EMA
Random Starts Specifications
  Number of initial stage random starts                 2000
  Number of final stage optimizations                    500
  Number of initial stage iterations                     500
  Initial stage convergence criterion              0.100D+01
  Random starts scale                              0.500D+01
  Random seed for generating random starts                 0

Input data file(s)
  mplus2.csv
Input data format  FREE

SUMMARY OF DATA

     Number of missing data patterns             4
     Number of y missing data patterns           4
     Number of u missing data patterns           0

COVARIANCE COVERAGE OF DATA

Minimum covariance coverage value   0.100

     PROPORTION OF DATA PRESENT FOR Y

           Covariance Coverage
              SLPNSSNC      WORRYNC       SOLC          SLPDURC
              ________      ________      ________      ________
 SLPNSSNC       1.000
 WORRYNC        1.000         1.000
 SOLC           0.976         0.976         0.976
 SLPDURC        0.963         0.963         0.940         0.963

UNIVARIATE SAMPLE STATISTICS

     UNIVARIATE HIGHER-ORDER MOMENT DESCRIPTIVE STATISTICS

         Variable/         Mean/     Skewness/   Minimum/ % with                Percentiles
        Sample Size      Variance    Kurtosis    Maximum   Min/Max      20%/60%    40%/80%    Median

     SLPNSSNC              0.933       0.086       0.000   27.10%       0.000      1.000      1.000
           27802.000       0.471      -0.878       2.000   20.40%       1.000      2.000
     WORRYNC               0.000      -1.036      -2.825    1.71%      -0.928      0.020      0.495
           27802.000       1.000       0.165       0.969   28.55%       0.495      0.969
     SOLC                  0.000       0.967      -1.054   31.63%      -1.054     -0.072     -0.072
           27123.000       1.000       0.467       2.875    2.77%      -0.072      0.910
```

```
    SLPDURC            0.000      0.568     -1.205   25.70%
-1.205     -0.292     -0.292
         26763.000      1.000     -0.470      2.445    3.44%
-0.292      0.620

RANDOM STARTS RESULTS RANKED FROM THE BEST
TO THE WORST LOGLIKELIHOOD VALUES

Unperturbed starting value run did not converge in the initial
stage
optimizations.

1760 perturbed starting value run(s) did not converge in the
initial stage
optimizations.

Final stage loglikelihood values at local maxima, seeds, and
initial stage start numbers:

    -124083.504  285763         1486
    -124083.504  530559         1845
    -124083.504  464810         1424
    -124083.504  453260         1106
    -124083.504  627151         1601
    -124083.504  317868         740
    -124083.504  628997         1188
    -124083.504  393453         1355
    -124083.504  153942         31
    -124083.504  884619         1441
    -124083.504  848331         137
    -124083.504  929823         1205
    -124083.504  922596         456
    -124083.504  716616         1159
    -124083.504  718876         1104
    -124083.504  539389         544
    -124083.504  268664         1784
    -124083.504  225932         1285
    -124083.504  712531         631
    -124083.504  626208         698
    -124083.504  210870         383
    -124083.504  185331         1162
    -124083.504  778331         1280
    -124083.504  881886         608
    -124083.504  930872         277
    -124083.504  685657         69
    -124083.504  526265         1962
    -124083.504  760599         832
    -124083.504  175820         1040
    -124083.504  696830         668
    -124083.504  879211         453
    -124083.504  676560         1250
    -124083.504  507218         613
    -124083.504  327927         908
    -124083.504  927045         1994
    -124083.504  273669         1118
    -124083.504  188498         258
    -124083.504  291112         645
    -124083.504  276243         1768
    -124083.504  782821         272
    -124083.504  266349         1989
    -124083.504  645052         910
    -124083.504  399071         1007
    -124083.504  547354         1357
    -124083.504  165822         1803
    -124083.504  61710          1327
    -124083.504  16096          1147
    -124083.504  967902         52
    -124083.504  384859         1530
    -124083.504  424280         1245
    -124083.504  680987         1926
    -124083.504  300844         1405
    -124083.504  301180         236
    -124083.504  583281         1907
    -124083.504  256197         1923
    -124083.504  908674         1748
    -124083.504  793487         595
    -124083.504  992389         77
    -124083.504  150531         154
    -124083.504  929361         1971
    -124083.504  964999         1522
    -124083.504  325724         1043
    -124083.504  876943         650
    -124083.504  568279         1580
    -124083.504  306768         978
    -124083.504  547702         767
    -124083.504  549244         756
    -124083.504  526742         1546
    -124083.504  922042         492
    -124083.504  848163         47
    -124083.504  105435         265
    -124083.504  393232         152
    -124083.504  501995         791
    -124083.504  222513         1088
    -124083.504  59963          670
    -124083.504  919217         1501
    -124083.504  238317         812
    -124083.504  354208         196
    -124083.504  290210         1620
    -124083.504  855963         1408
    -124083.504  990504         827
    -124083.504  81951          907
    -124083.504  251680         794
    -124083.504  51375          148
    -124083.504  856347         1927
    -124083.504  760850         739
    -124083.504  981480         1025
    -124096.895  702409         1227
    -124096.895  117891         1818
    -124096.895  669634         335
    -124096.895  131063         843
    -124096.895  126922         1124
    -124096.895  331689         1872
    -124096.895  303429         1016
    -124096.895  765589         1140
    -124096.895  165853         105
    -124096.895  570681         777
    -124096.895  84927          1511
    -124096.895  349263         263
    -124096.895  928085         1946
    -124096.895  956446         1081
    -124096.895  164643         1941
    -124096.895  358115         1724
    -124096.895  197904         1782
    -124096.895  769907         457
    -124096.895  341041         34
    -124096.895  765372         1123
    -124096.895  159216         1423
    -124096.895  441118         1091
    -124096.895  63231          935
    -124096.895  964389         1223
    -124096.895  813351         1507
    -124096.895  95624          1605
    -124096.895  674786         1496
    -124096.895  879338         309
    -124096.895  629320         222
    -124096.895  662718         460
    -124096.895  644297         340
    -124096.895  558208         1403
    -124096.895  53089          1660
    -124096.895  471398         74
```

-124096.895	933578	506
-124096.895	814975	129
-124096.895	518828	432
-124096.895	166220	1502
-124096.895	96617	955
-124096.895	496710	386
-124096.895	510491	1275
-124096.895	752847	1109
-124096.895	848356	602
-124096.895	654377	1684
-124096.895	271809	846
-124096.895	699748	1120
-124096.895	678813	1881
-124096.895	621542	375
-124096.895	358688	1573
-124096.895	937885	426
-124096.895	435506	988
-124096.895	521770	945
-124096.895	188490	1460
-124096.895	493775	1402
-124096.895	910119	1886
-124096.895	246261	38
-124096.895	792993	859
-124096.895	51011	1111
-124096.895	153053	378
-124096.895	970689	266
-124096.895	528938	1898
-124709.900	945711	1419
-124709.900	996231	310
-124709.900	611687	1933
-124709.900	569833	85
-124709.900	469158	1145
-124709.900	451258	848
-124709.900	137305	379
-124709.900	531546	702
-124709.900	207299	1039
-124709.900	938409	1144
-124709.900	135076	1692
-125293.398	842091	1373
-125293.398	677720	681
-125394.935	232559	136
-125394.935	881268	1299
-125994.415	489927	520
-125994.415	181178	1069
-126943.690	91231	727

334 perturbed starting value run(s) did not converge or were rejected in the third stage.

THE BEST LOGLIKELIHOOD VALUE HAS BEEN REPLICATED. RERUN WITH AT LEAST TWICE THE RANDOM STARTS TO CHECK THAT THE BEST LOGLIKELIHOOD IS STILL OBTAINED AND REPLICATED.

THE MODEL ESTIMATION TERMINATED NORMALLY

MODEL FIT INFORMATION

Number of Free Parameters 58

Loglikelihood

H0 Value -124083.504
H0 Scaling Correction Factor 1.1989
for MLR

Information Criteria

Akaike (AIC) 248283.007
Bayesian (BIC) 248760.513
Sample-Size Adjusted BIC 248576.190
(n* = (n + 2) / 24)

FINAL CLASS COUNTS AND PROPORTIONS FOR THE LATENT CLASSES
BASED ON THE ESTIMATED MODEL

Latent Classes		
1	1300.32515	0.04677
2	4426.85090	0.15923
3	1645.56536	0.05919
4	2323.29222	0.08357
5	18105.96637	0.65125

FINAL CLASS COUNTS AND PROPORTIONS FOR THE LATENT CLASSES
BASED ON ESTIMATED POSTERIOR PROBABILITIES

Latent Classes		
1	1300.32515	0.04677
2	4426.85090	0.15923
3	1645.56536	0.05919
4	2323.29222	0.08357
5	18105.96637	0.65125

FINAL CLASS COUNTS AND PROPORTIONS FOR THE LATENT CLASSES
BASED ON THEIR MOST LIKELY LATENT CLASS MEMBERSHIP

Class Counts and Proportions

Latent Classes		
1	1037	0.03730
2	4825	0.17355
3	1250	0.04496
4	2335	0.08399
5	18355	0.66020

CLASSIFICATION QUALITY

Entropy 0.860

Average Latent Class Probabilities for Most Likely Latent Class Membership (Row)
by Latent Class (Column)

	1	2	3	4	5
1	0.782	0.142	0.001	0.075	0.000
2	0.061	0.778	0.052	0.023	0.085
3	0.002	0.036	0.905	0.043	0.014
4	0.081	0.019	0.008	0.891	0.000
5	0.000	0.024	0.013	0.000	0.963

Classification Probabilities for the Most Likely Latent Class Membership (Column)
by Latent Class (Row)

	1	2	3	4	5
1	0.624	0.228	0.002	0.146	0.000
2	0.033	0.848	0.010	0.010	0.098

```
   3  0.000   0.152   0.687   0.012   0.148
   4  0.033   0.048   0.023   0.896   0.000
   5  0.000   0.023   0.001   0.000   0.976

Logits for the Classification Probabilities for the Most Likely
Latent Class Membership (Column)
by Latent Class (Row)

            1        2        3        4        5

   1     9.660    8.655    3.760    8.210    0.000
   2    -1.078    2.158   -2.261   -2.280    0.000
   3    -5.926    0.029    1.535   -2.541    0.000
   4     9.727   10.084    9.366   13.018    0.000
   5   -13.507   -3.761   -6.925  -12.691    0.000

MODEL RESULTS

                                         Two-Tailed
                   Estimate       S.E.  Est./S.E.    P-Value

Latent Class 1

 SLPNSSNC WITH
    WORRYNC            0.028      0.014      2.097      0.036
    SOLC               0.054      0.019      2.863      0.004
    SLPDURC           -0.004      0.021     -0.173      0.863

 WORRYNC  WITH
    SOLC               0.018      0.023      0.760      0.447
    SLPDURC            0.011      0.017      0.603      0.546

 SOLC     WITH
    SLPDURC            0.029      0.023      1.279      0.201

 Means
    SLPNSSNC           0.941      0.041     23.047      0.000
    WORRYNC           -1.812      0.077    -23.556      0.000
    SOLC               0.058      0.078      0.741      0.459
    SLPDURC            0.950      0.069     13.849      0.000

 Variances
    SLPNSSNC           0.460      0.003    150.607      0.000
    WORRYNC            0.183      0.003     66.958      0.000
    SOLC               0.414      0.004     99.215      0.000
    SLPDURC            0.752      0.009     82.198      0.000

Latent Class 2

 SLPNSSNC WITH
    WORRYNC            0.009      0.009      1.072      0.284
    SOLC               0.088      0.008     10.716      0.000
    SLPDURC            0.029      0.010      2.814      0.005

 WORRYNC  WITH
    SOLC              -0.074      0.009     -8.090      0.000
    SLPDURC           -0.007      0.014     -0.457      0.648

 SOLC     WITH
    SLPDURC            0.096      0.013      7.505      0.000

 Means
    SLPNSSNC           0.875      0.015     58.846      0.000
    WORRYNC           -0.749      0.035    -21.694      0.000
    SOLC               0.032      0.029      1.135      0.256
    SLPDURC            0.332      0.033      9.996      0.000

 Variances
    SLPNSSNC           0.460      0.003    150.607      0.000
    WORRYNC            0.183      0.003     66.958      0.000
    SOLC               0.414      0.004     99.215      0.000
    SLPDURC            0.752      0.009     82.198      0.000

Latent Class 3

 SLPNSSNC WITH
    WORRYNC           -0.002      0.007     -0.314      0.753
    SOLC              -0.015      0.019     -0.760      0.447
    SLPDURC           -0.030      0.016     -1.880      0.060

 WORRYNC  WITH
    SOLC              -0.004      0.008     -0.475      0.635
    SLPDURC           -0.076      0.010     -7.544      0.000

 SOLC     WITH
    SLPDURC            0.012      0.020      0.592      0.554

 Means
    SLPNSSNC           1.209      0.022     54.036      0.000
    WORRYNC           -0.224      0.037     -5.997      0.000
    SOLC               1.723      0.027     63.438      0.000
    SLPDURC            0.439      0.044      9.899      0.000

 Variances
    SLPNSSNC           0.460      0.003    150.607      0.000
    WORRYNC            0.183      0.003     66.958      0.000
    SOLC               0.414      0.004     99.215      0.000
    SLPDURC            0.752      0.009     82.198      0.000

Latent Class 4

 SLPNSSNC WITH
    WORRYNC            0.004      0.006      0.633      0.527
    SOLC              -0.007      0.014     -0.517      0.605
    SLPDURC            0.013      0.013      1.000      0.317

 WORRYNC  WITH
    SOLC              -0.050      0.007     -6.747      0.000
    SLPDURC           -0.072      0.008     -9.171      0.000

 SOLC     WITH
    SLPDURC            0.024      0.014      1.765      0.078

 Means
    SLPNSSNC           1.167      0.017     69.498      0.000
    WORRYNC           -2.030      0.021    -97.318      0.000
    SOLC               1.944      0.024     81.105      0.000
    SLPDURC            1.173      0.026     45.965      0.000

 Variances
    SLPNSSNC           0.460      0.003    150.607      0.000
    WORRYNC            0.183      0.003     66.958      0.000
    SOLC               0.414      0.004     99.215      0.000
    SLPDURC            0.752      0.009     82.198      0.000

Latent Class 5

 SLPNSSNC WITH
    WORRYNC           -0.016      0.003     -5.075      0.000
    SOLC               0.074      0.004     19.537      0.000
    SLPDURC            0.017      0.005      3.071      0.002

 WORRYNC  WITH
    SOLC              -0.136      0.003    -43.443      0.000
    SLPDURC           -0.150      0.005    -32.481      0.000

 SOLC     WITH
    SLPDURC            0.195      0.006     33.001      0.000

 Means
```

```
    SLPNSSNC           0.892      0.005    163.537      0.000
    WORRYNC            0.594      0.005    123.078      0.000
    SOLC              -0.411      0.006    -74.068      0.000
    SLPDURC           -0.334      0.007    -45.482      0.000

 Variances
    SLPNSSNC           0.460      0.003    150.607      0.000
    WORRYNC            0.183      0.003     66.958      0.000
    SOLC               0.414      0.004     99.215      0.000
    SLPDURC            0.752      0.009     82.198      0.000

Categorical Latent Variables

 Means
    C#1               -2.634      0.144    -18.330      0.000
    C#2               -1.409      0.032    -44.014      0.000
    C#3               -2.398      0.042    -56.710      0.000
    C#4               -2.053      0.032    -63.375      0.000


QUALITY OF NUMERICAL RESULTS

     Condition Number for the Information Matrix
0.996E-05
       (ratio of smallest to largest eigenvalue)

TECHNICAL 11 OUTPUT

     Random Starts Specifications for the k-1 Class Analysis
Model
        Number of initial stage random starts                 2000
        Number of final stage optimizations                    500

     VUONG-LO-MENDELL-RUBIN LIKELIHOOD RATIO
TEST FOR 4 (H0) VERSUS 5 CLASSES

          H0 Loglikelihood Value                      -124709.900
          2 Times the Loglikelihood Difference           1252.794
          Difference in the Number of Parameters               11
          Mean                                             26.542
          Standard Deviation                               38.528
          P-Value                                          0.0000

     LO-MENDELL-RUBIN ADJUSTED LRT TEST

          Value                                          1241.762
          P-Value                                          0.0000

TECHNICAL 14 OUTPUT

     Random Starts Specifications for the k-1 Class Analysis
Model
        Number of initial stage random starts                 2000
        Number of final stage optimizations                    500

     Random Starts Specification for the k-1 Class Model for
Generated Data
        Number of initial stage random starts                    4
        Number of final stage optimizations                      2
     Random Starts Specification for the k Class Model for
Generated Data
        Number of initial stage random starts                  100
        Number of final stage optimizations                     20
     Number of bootstrap draws requested                       500

     PARAMETRIC BOOTSTRAPPED LIKELIHOOD RATIO
TEST FOR 4 (H0) VERSUS 5 CLASSES

          H0 Loglikelihood Value                      -124709.900
          2 Times the Loglikelihood Difference           1252.794
          Difference in the Number of Parameters               11
          Approximate P-Value                              0.0000
          Successful Bootstrap Draws                          500

     WARNING:  OF THE 500 BOOTSTRAP DRAWS, 459
DRAWS HAD BOTH A SMALLER LRT VALUE THAN THE
     OBSERVED LRT VALUE AND NOT A REPLICATED
BEST LOGLIKELIHOOD VALUE FOR THE 5-CLASS
MODEL.
     THIS MEANS THAT THE P-VALUE MAY NOT BE
TRUSTWORTHY DUE TO LOCAL MAXIMA.
     INCREASE THE NUMBER OF RANDOM STARTS
USING THE LRTSTARTS OPTION.

PLOT INFORMATION

The following plots are available:

  Histograms (sample values)
  Scatterplots (sample values)
  Sample means
  Estimated means, medians, modes, and percentiles
  Sample and estimated means
  Observed individual values
  Estimated means and observed individual values
  Estimated overall and class-specific distributions

SAVEDATA INFORMATION

  Save file
    Step2_LPA5M5.dat

  Order and format of variables

    SLPNSSNC       F10.3
    WORRYNC        F10.3
    SOLC           F10.3
    SLPDURC        F10.3
    CPROB1         F10.3
    CPROB2         F10.3
    CPROB3         F10.3
    CPROB4         F10.3
    CPROB5         F10.3
    C              F10.3
    ID             I6

  Save file format
    10F10.3 I6

  Save file record length    10000

  Save missing symbol        *

DIAGRAM INFORMATION

  Mplus diagrams are currently not available for Mixture
analysis.
  No diagram output was produced.

     Beginning Time:  20:14:35
        Ending Time:  20:57:03
       Elapsed Time:  24:42:28

MUTHEN & MUTHEN
3463 Stoner Ave.
Los Angeles, CA  90066

Tel: (310) 391-9971
Fax: (310) 391-8971
Web: www.StatModel.com
```

Support: Support@StatModel.com Copyright (c) 1998-2021 Muthen & Muthen

Six-profile model

```
Mplus VERSION 8.7
MUTHEN & MUTHEN
07/15/2023   1:26 PM

INPUT INSTRUCTIONS

  TITLE: LPA 6 profile syntax: M5: Varying means, equal
variances, and varying covariances

  DATA:
  FILE IS mplus2.csv;
  ! Specifies file location for data file. Make sure data is in
format appropriate for Mplus
  ! per Mplus manual. This data file is in individual format
(one row of data per participan
  VARIABLE:
  NAMES ARE ID SCI_02 SCI_02B slpnssN worryN sol
slpDur gender genderN yrgrp yrgrpN SCI_02C
  slpnssNC worryNC solC slpDurC;

  ! All variables included in data file should be named here.
  USEVARIABLES ARE slpnssNC worryNC solC slpDurC;
  ! Only variables intended for use in the analysis should be
listed here
  IDVARIABLE IS ID;
  CLASSES = c (6);
  ! This is where you instruct Mplus on how many
classes/profiles are being estimated. Initi
  ! model contains only one class/profile, thus it would be
CLASSES = c (1). Above specifies
  !profiles, and for each further iterative models the number
in parentheses increases by on
  !three profiles/classes would be c (3), and so on.
  MISSING ARE ALL(9999);
  ! Used to communicate how missing data is coded in data
file. Here shown with a "." which
  ! all that is included in each cell with missing data in the
data file
  ANALYSIS:
  TYPE = MIXTURE;
  ! LPA is a version of mixture modeling, and this instructs
Mplus to analyze in this way
  ESTIMATOR = MLR;
  !FIML robust to non-normal data
  STARTS = 8000 2000;
  STITERATIONS = 500;
  ! Default number of starts for each step of the ML
estimation. First STARTS value specifie
  !number of unique start values to start with, the 250
represents the 250 best unique start
  !carrying forward to completion. The STITERATIONS
specifies the number of ML iteration
  !steps for those 250 selected start values to go through to
be able to converge. This is a
  !maximum number of iteration; if a model converges in less
than 500 iterations it will sto
  !before reaching 500 iterations.
  !These values can be increased … see "Four-Profile Final
Model with Covariate Analysis
  !Syntax" for an example.
  LRTSTARTS = 16 8 400 80;
  LRTBOOTSTRAP = 500;
  !The above start values are for the defaults for the LRT
statistic being run to compare th
  !model fit with the model fit of a model with one less class
(k-1). The BOOTSTRAP statemen
  !specifies the number of bootstrap draws to inform Mplus'
bootstrapped LRT results.
  MODEL:
  !For a default Mplus model the LPA model does not need
to be specified. However, it can be
  !The model can also be modified from the Mplus default of
estimating the indicator means
  !(uniquely across profiles) and variances (constrained
across profiles), as well as the la
  !profile mean.
  %OVERALL%
  [slpnssNC worryNC solC slpDurC]; !estimates the
indicators means for each profile. Without
  !the means are freely estimated in each profile, not
constrained.
  slpnssNC worryNC solC slpDurC; !Label Var1-Var5
constrains the estimates of the variances
  !profiles to be equal.

  %c#1%

    [slpnssNC worryNC solC slpDurC];

    !covariances
  slpnssNC WITH worryNC solC slpDurC;
  worryNC WITH solC slpDurC;
  solC WITH slpDurC;

   %c#2%

    [slpnssNC worryNC solC slpDurC];

    !covariances
  slpnssNC WITH worryNC solC slpDurC;
  worryNC WITH solC slpDurC;
  solC WITH slpDurC;

  %c#3%

    [slpnssNC worryNC solC slpDurC];

    !covariances
  slpnssNC WITH worryNC solC slpDurC;
  worryNC WITH solC slpDurC;
  solC WITH slpDurC;

   %c#4%

    [slpnssNC worryNC solC slpDurC];

    !covariances
  slpnssNC WITH worryNC solC slpDurC;
  worryNC WITH solC slpDurC;
  solC WITH slpDurC;

   %c#5%

    [slpnssNC worryNC solC slpDurC];

    !covariances
  slpnssNC WITH worryNC solC slpDurC;
  worryNC WITH solC slpDurC;
```

```
  solC WITH slpDurC;

   %c#6%

     [slpnssNC worryNC solC slpDurC];

     !covariances
  slpnssNC WITH worryNC solC slpDurC;
  worryNC WITH solC slpDurC;
  solC WITH slpDurC;

  OUTPUT:
  TECH11 TECH14;
  ! TECH1 provides parameter specifications and starting
values for the analysis
  ! TECH8 provides optimization history for this analysis type
  !TECH11 provides LRT results
  !TECH14 provides bootstrapped LRT test
  PLOT: SERIES=slpnssNC worryNC solC slpDurC(*);
  TYPE=PLOT3;
  SAVEDATA:
  FILE IS Step2_LPA6M5.dat;
  ! Tells Mplus where to save the output files from the
analysis
  SAVE = CPROBABILITIES;
  ! The above command lines are to save the most likely
profile membership for each particip
  ! and the posterior probabilities for their membership in
each latent profile.

*** WARNING in MODEL command
  All variables are uncorrelated with all other variables within
class.
  Check that this is what is intended.

LPA 6 profile syntax: M5: Varying means, equal variances,
and varying covariances

SUMMARY OF ANALYSIS

Number of groups                                  1
Number of observations                        27802

Number of dependent variables                     4
Number of independent variables                   0
Number of continuous latent variables             0
Number of categorical latent variables            1

Observed dependent variables

  Continuous
   SLPNSSNC    WORRYNC     SOLC       SLPDURC

Categorical latent variables
   C

Variables with special functions

  ID variable          ID

Estimator                                       MLR
Information matrix                         OBSERVED
Optimization Specifications for the Quasi-Newton Algorithm
for
Continuous Outcomes
  Maximum number of iterations                  100
  Convergence criterion                   0.100D-05
Optimization Specifications for the EM Algorithm
  Maximum number of iterations                  500
  Convergence criteria
    Loglikelihood change                  0.100D-06
    Relative loglikelihood change         0.100D-06
    Derivative                            0.100D-05
Optimization Specifications for the M step of the EM
Algorithm for
Categorical Latent variables
  Number of M step iterations                     1
  M step convergence criterion            0.100D-05
  Basis for M step termination            ITERATION
Optimization Specifications for the M step of the EM
Algorithm for
Censored, Binary or Ordered Categorical (Ordinal),
Unordered
Categorical (Nominal) and Count Outcomes
  Number of M step iterations                     1
  M step convergence criterion            0.100D-05
  Basis for M step termination            ITERATION
  Maximum value for logit thresholds             15
  Minimum value for logit thresholds            -15
  Minimum expected cell size for chi-square  0.100D-
01
Maximum number of iterations for H1            2000
Convergence criterion for H1              0.100D-03
Optimization algorithm                          EMA
Random Starts Specifications
  Number of initial stage random starts        8000
  Number of final stage optimizations          2000
  Number of initial stage iterations            500
  Initial stage convergence criterion     0.100D+01
  Random starts scale                     0.500D+01
  Random seed for generating random starts        0

Input data file(s)
  mplus2.csv
Input data format  FREE

SUMMARY OF DATA

     Number of missing data patterns           4
     Number of y missing data patterns         4
     Number of u missing data patterns         0

COVARIANCE COVERAGE OF DATA

Minimum covariance coverage value   0.100

     PROPORTION OF DATA PRESENT FOR Y


           Covariance Coverage
              SLPNSSNC      WORRYNC       SOLC
SLPDURC
              ________      ________      ________      ________
 SLPNSSNC       1.000
 WORRYNC        1.000         1.000
 SOLC           0.976         0.976         0.976
 SLPDURC        0.963         0.963         0.940         0.963

UNIVARIATE SAMPLE STATISTICS

     UNIVARIATE HIGHER-ORDER MOMENT
DESCRIPTIVE STATISTICS

         Variable/         Mean/     Skewness/   Minimum/ % with
Percentiles
        Sample Size      Variance    Kurtosis    Maximum
Min/Max      20%/60%    40%/80%    Median

     SLPNSSNC             0.933       0.086       0.000   27.10%
0.000      1.000      1.000
```

```
        27802.000      0.471      -0.878       2.000   20.40%
1.000      2.000
    WORRYNC             0.000      -1.036      -2.825    1.71%
-0.928      0.020      0.495
        27802.000      1.000       0.165       0.969   28.55%
0.495      0.969
    SOLC               0.000       0.967      -1.054   31.63%      -
1.054     -0.072     -0.072
        27123.000      1.000       0.467       2.875    2.77%
-0.072      0.910
    SLPDURC             0.000       0.568      -1.205   25.70%
-1.205     -0.292     -0.292
        26763.000      1.000      -0.470       2.445    3.44%
-0.292      0.620

RANDOM STARTS RESULTS RANKED FROM THE BEST
TO THE WORST LOGLIKELIHOOD VALUES

Unperturbed starting value run did not converge in the initial
stage
optimizations.

7502 perturbed starting value run(s) did not converge in the
initial stage
optimizations.

Final stage loglikelihood values at local maxima, seeds, and
initial stage start numbers:

        -105840.802  102504           7215
        -123380.204  347110           3761
        -123380.204  366872           1169
        -123380.204  630834           2964
        -123380.204  45556           3294
        -123380.204  530305           6477
        -123380.204  209545           7123
        -123380.204  689589           3947
        -123380.204  419979           5094
        -123380.204  632565           4724
        -123380.204  460630           3542
        -123380.204  470921           2993
        -123380.204  929499           3974
        -123380.204  166700           7162
        -123380.204  231366           1137
        -123380.204  640797           4654
        -123380.204  474013           7485
        -123380.204  977507           1716
        -123380.204  110260           2535
        -123380.204  231986           7210
        -123380.204  81598           7240
        -123380.204  855715           6992
        -123380.204  607148           6862
        -123380.204  29275           6028
        -123380.204  863480           5377
        -123380.204  25584           7402
        -123380.204  466035           6850
        -123380.204  933578           506
        -123380.204  690608           1800
        -123380.204  848969           173
        -123380.204  220633           5604
        -123380.204  567252           1843
        -123380.204  978671           5259
        -123380.204  789564           2275
        -123380.204  719104           6684
        -123380.204  12814           4104
        -123380.204  899062           3493
        -123380.204  165822           1803
        -123380.204  42278           1305
        -123380.204  88178           5619
        -123380.204  473869           3707
        -123380.204  279725           5288
        -123380.204  548301           5906
        -123380.204  270257           5176
        -123380.204  682047           2666
        -123380.204  9427           4643
        -123380.204  880807           6726
        -123380.204  550354           4790
        -123380.204  120368           6622
        -123380.204  797872           7655
        -123380.204  463894           1733
        -123380.204  876943           650
        -123380.204  400299           4782
        -123380.204  854941           6096
        -123380.204  217990           1077
        -123380.204  907407           2624
        -123380.204  54085           5617
        -123380.204  755611           3182
        -123380.204  551340           766
        -123380.204  542577           1004
        -123380.204  766178           1937
        -123380.204  512048           3172
        -123380.204  678595           5688
        -123380.204  153053           378
        -123380.204  444228           860
        -123380.204  129163           4601
        -123380.204  139941           3271
        -123380.204  563452           7834
        -123380.204  846789           7904
        -123380.204  715466           5857
        -123380.204  728282           5901
        -123380.204  430691           7901
        -123380.204  8370           4917
        -123380.204  776607           7866
        -123380.204  254297           4551
        -123380.204  420298           1222
        -123380.204  407520           2854
        -123380.204  294397           7351
        -123380.204  352277           42
        -123380.204  62571           3923
        -123380.204  937651           5704
        -123380.204  280104           1875
        -123380.204  848163           47
        -123380.204  953815           1347
        -123380.204  510814           3219
        -123380.204  988209           7713
        -123380.204  530559           1845
        -123380.204  354693           2038
        -123380.204  736635           7286
        -123380.204  666720           671
        -123380.204  970838           3615
        -123380.204  357565           7348
        -123380.204  697272           2010
        -123380.204  480926           1824
        -123380.204  748083           4737
        -123380.204  413363           2884
        -123380.204  751592           3458
        -123380.204  876447           6025
        -123380.204  37806           4535
        -123380.204  634782           979
        -123380.204  485948           3188
        -123380.204  908524           6156
        -123380.204  314551           3955
        -123380.204  619187           7233
        -123380.204  937476           7543
        -123380.204  760850           739
        -123380.204  717754           180
        -123380.204  390788           3191
        -123380.204  564582           7832
        -123380.204  938366           6817
        -123380.204  356418           7958
```

-123380.204	787782	3419
-123380.204	634094	7507
-123380.204	829063	2276
-123380.204	739458	1963
-123380.204	362529	2736
-123380.204	321363	1095
-123380.204	337438	4320
-123380.204	734978	5042
-123380.204	286505	2416
-123380.204	794411	5790
-123380.204	721907	4479
-123380.204	76422	5258
-123380.204	310591	4901
-123380.204	925741	1567
-123380.204	450584	3772
-123380.204	244349	736
-123380.204	513053	5961
-123380.204	900041	3785
-123380.204	821111	6824
-123380.204	402776	7710
-123380.204	687000	5863
-123380.204	891347	504
-123380.204	76204	2003
-123380.204	701586	5384
-123380.204	7510	2482
-123380.204	962359	4118
-123380.204	187001	4588
-123380.204	81648	5344
-123380.204	252552	2489
-123380.204	476538	5063
-123380.204	712066	2494
-123380.204	950604	172
-123380.204	475953	3687
-123380.204	861413	1154
-123380.204	18840	3536
-123380.204	24045	2066
-123380.204	374630	1869
-123380.204	145690	7951
-123380.204	61710	1327
-123380.204	25294	2192
-123380.204	803696	1340
-123380.204	43897	2230
-123380.204	589839	4722
-123380.204	763802	4958
-123380.204	369266	7511
-123380.204	344416	3290
-123380.204	603416	6452
-123380.204	955504	7773
-123380.204	573791	6121
-123380.204	164467	2047
-123380.204	501995	791
-123380.204	307045	7189
-123380.204	691544	2183
-123380.204	757590	2804
-123380.204	584751	4113
-123380.204	643858	5665
-123380.204	439675	7827
-123380.204	17896	592
-123380.204	166096	4361
-123380.204	389980	7164
-123380.204	438631	7526
-123380.204	486133	7364
-123380.204	602960	2226
-123380.204	785023	3454
-123380.204	262076	2015
-123380.204	438144	271
-123380.204	801057	6339
-123380.204	488565	5839
-123380.204	760380	3885
-123380.204	887787	5625
-123380.204	692399	3763
-123380.204	463203	5945
-123380.204	120100	5289
-123380.204	832850	2779
-123380.204	173191	422
-123380.204	885436	4377
-123380.204	626490	4476
-123380.204	749808	3344
-123380.204	140694	5735
-123380.204	323788	3307
-123380.204	573976	1217
-123380.204	42579	4794
-123380.204	882244	6464
-123380.204	703618	7115
-123380.204	566784	6618
-123380.204	469648	6565
-123380.204	670998	1876
-123380.204	655088	7777
-123380.204	412313	6615
-123380.204	477242	4717
-123380.204	627224	4579
-123380.204	231581	1048
-123380.204	824956	607
-123380.204	995881	4723
-123380.204	177175	851
-123380.204	529627	1542
-123380.204	900432	2808
-123380.204	470570	3598
-123380.204	880440	5602
-123380.204	963975	6544
-123380.204	976112	4060
-123380.204	417391	7371
-123380.204	603765	1006
-123380.204	863378	2556
-123380.204	738142	1970
-123380.204	633988	6030
-123380.204	828340	1559
-123380.204	804616	868
-123380.204	399071	1007
-123380.204	683728	5363
-123380.204	681079	3005
-123380.204	838356	5513
-123380.204	660633	5171
-123380.204	659469	5608
-123380.204	287220	4395
-123380.204	606153	5028
-123380.204	335396	5902
-123380.204	973369	202
-123380.204	526836	6690
-123380.204	576306	3678
-123380.204	695314	6891
-123380.204	697866	1018
-123380.204	927045	1994
-123380.204	669634	335
-123380.204	159216	1423
-123380.204	802250	7675
-123380.204	200114	6733
-123380.204	860732	2774
-123380.204	587497	5903
-123380.204	978781	497
-123380.204	427273	2524
-123380.204	869821	6237
-123380.204	955439	7882
-123380.204	498982	4136
-123380.204	10606	4208
-123380.204	581912	3149
-123380.204	769417	4111
-123380.204	561782	6822
-123380.204	234123	7681
-123380.204	914462	3094

-123380.204	950970	2963
-123380.204	234995	4728
-123380.204	779878	6421
-123380.204	925265	5017
-123380.204	365050	1835
-123380.204	3307	735
-123380.204	652179	5703
-123380.204	208803	6223
-123380.204	862495	3657
-123380.204	127619	5679
-123380.204	596257	405
-123380.204	778331	1280
-123380.204	171251	5015
-123380.204	726787	1550
-123380.204	145560	5410
-123380.204	616776	3283
-123380.204	385116	7397
-123380.204	88407	6844
-123380.204	136094	7099
-123380.204	321574	1185
-123380.204	791087	4308
-123380.204	844630	7711
-123380.204	236935	3873
-123380.204	271769	7649
-123380.204	286163	6626
-123380.204	555074	6182
-123380.204	526265	1962
-123380.204	922042	492
-123380.204	51375	148
-123380.204	277405	5638
-123380.204	957757	6403
-123380.204	208737	7212
-123380.204	981709	6322
-123380.204	370434	1832
-123380.204	761633	50
-123380.204	992389	77
-123380.204	609004	2777
-123380.204	164582	7991
-123380.204	949412	4952
-123380.204	435399	2896
-123380.204	296924	1702
-123380.204	646573	741
-123380.204	46908	3949
-123380.204	789171	2365
-123380.204	697914	6806
-123380.204	448012	7413
-123380.204	303628	7673
-123380.204	626264	3014
-123380.204	25530	4179
-123380.204	71845	7650
-123380.204	527412	7073
-123380.204	850953	4066
-123380.204	477475	6934
-123380.204	524904	4913
-123380.204	832635	3239
-123380.204	602588	5436
-123380.204	417342	6942
-123380.204	422776	5742
-123380.204	115831	3223
-123380.204	612202	4269
-123380.204	322964	3451
-123380.204	392717	4834
-123380.204	988709	2430
-123380.204	612837	3056
-123380.204	548625	7854
-123380.204	911887	6629
-123380.204	170485	6091
-123380.204	317070	7733
-123380.204	245802	5336
-123380.204	662807	4554

-123380.204	437885	7147
-123380.204	953205	2247
-123380.204	383658	6186
-123380.204	179332	4881
-123380.204	518828	432
-123380.204	579995	183
-123380.204	423665	3152
-123380.204	360476	3647
-123380.204	166220	1502
-123380.204	204860	5437
-123380.204	690707	5370
-123380.204	879211	453
-123380.204	626891	32
-123380.204	395914	5801
-123380.204	907342	1133
-123380.204	718876	1104
-123380.204	863691	481
-123380.204	367683	4650
-123380.204	105392	7874
-123380.204	393318	4188
-123380.204	117891	1818
-123380.204	746304	3335
-123380.204	137790	5503
-123380.204	785068	3000
-123380.204	63470	4478
-123380.204	807339	4334
-123380.204	227563	63
-123380.204	754616	4405
-123380.204	383371	3818
-123380.204	197794	4439
-123380.204	798091	5260
-123380.204	139080	7105
-123380.204	735663	4273
-123380.204	99433	4520
-123380.204	309767	3240
-123380.204	715588	6020
-123380.204	232226	235
-123380.204	534583	6098
-123380.204	52311	7800
-123380.204	293349	2520
-123380.204	971770	2708
-123380.204	735406	4759
-123380.204	957792	2069
-123380.204	478421	311
-123380.204	396943	5911
-123380.204	997222	229
-123380.204	949086	1426
-123380.204	480046	2484
-123380.204	994880	5709
-123380.204	830618	3377
-123380.204	745674	3511
-123646.100	694918	1206
-123646.100	718336	2280
-123646.100	643311	888
-123646.100	123098	2191
-123646.100	789916	4259
-123646.100	570518	6831
-123646.100	10968	1492
-123646.100	447198	7824
-123656.753	471811	5132
-123793.364	571443	4528
-123793.364	311384	5074
-123793.364	600244	3830
-123793.364	393453	1355
-123793.364	943783	5797
-123817.802	188498	258
-123817.802	156983	5262
-123817.802	202790	198
-123817.802	80	2188
-123817.802	84256	2662

```
-123817.802  866824        7117
-123817.802  802771        4878
-123817.802  896300        5588
-123817.802  100054        1225
-123817.802  704526        6256
-123817.802  247709        6305
-123817.802  810668        4172
-123817.802  789701        6909
-123817.802  654884        1352
-123817.802  818908        5898
-123817.802  368364        4774
-123817.802  421731        886
-123817.802  662359        1954
-123817.802  218683        5325
-123817.802  462159        6130
-123817.802  362536        7653
-123817.802  402049        4468
-123817.802  763134        4903
-123817.802  157736        1524
-123817.802  115267        2689
-123817.802  667120        2859
-123817.802  515742        7693
-123817.802  940517        2728
-123817.802  167220        2608
-124083.504  937225        394
-124083.504  450164        5105
-124083.504  975027        1552
-124083.504  920593        611
-124083.504  429802        3983
-124083.504  924648        6655
-124083.504  945925        2583
-124083.504  269171        2307
-124096.895  662718        460
-124096.895  789081        6962
-124096.895  135288        5248
-124096.895  819788        4192
-124096.895  301105        4652
-124096.895  91231        727
-124096.895  886520        3171
-124096.895  429510        4040
-124096.895  299736        7688
-124096.895  785215        3364
-124096.895  883355        2140
-124117.383  311796        4910
-124117.383  109599        3448
-124117.383  676560        1250
-124117.383  272240        6376
-124241.254  737045        1638
-124241.254  808639        5924
-124241.254  887941        2425
-124241.254  504509        5610
-124241.254  730263        2641
-124241.254  187594        3708
-124367.958  50708        6449
-124608.382  406734        2605
-124709.900  954581        3583
-124709.900  692357        2081
-124709.900  903246        3176
-124709.900  970728        1371
-124709.900  517917        5157
-124709.900  933596        2349
-124709.900  232509        2745
-124709.900  472141        5833
-124709.900  723642        3817
-124709.900  181178        1069
-124709.900  767778        7158
-124709.900  124303        7695
-124709.900  21345        199

1541 perturbed starting value run(s) did not converge or
were rejected in the third stage.

     WARNING:  THE BEST LOGLIKELIHOOD VALUE WAS
NOT REPLICATED.  THE
     SOLUTION MAY NOT BE TRUSTWORTHY DUE TO
LOCAL MAXIMA.  INCREASE THE
     NUMBER OF RANDOM STARTS.

     THE STANDARD ERRORS OF THE MODEL
PARAMETER ESTIMATES MAY NOT BE
     TRUSTWORTHY FOR SOME PARAMETERS DUE TO
A NON-POSITIVE DEFINITE
     FIRST-ORDER DERIVATIVE PRODUCT MATRIX.  THIS
MAY BE DUE TO THE STARTING
     VALUES BUT MAY ALSO BE AN INDICATION OF
MODEL NONIDENTIFICATION.  THE
     CONDITION NUMBER IS       0.573D-10.  PROBLEM
INVOLVING THE FOLLOWING PARAMETER:
     Parameter 62, %C#6%: SLPDURC WITH SLPNSSNC


THE MODEL ESTIMATION TERMINATED NORMALLY

MODEL FIT INFORMATION

Number of Free Parameters                       69

Loglikelihood

          H0 Value                      -105840.802
          H0 Scaling Correction Factor      1.1409
            for MLR

Information Criteria

          Akaike (AIC)                   211819.605
          Bayesian (BIC)                 212387.673
          Sample-Size Adjusted BIC       212168.392
            (n* = (n + 2) / 24)

FINAL CLASS COUNTS AND PROPORTIONS FOR THE
LATENT CLASSES
BASED ON THE ESTIMATED MODEL

    Latent
   Classes

       1        6256.52283          0.22504
       2        2512.95967          0.09039
       3        3788.57316          0.13627
       4        7106.88021          0.25562
       5        3150.58120          0.11332
       6        4986.48292          0.17936

FINAL CLASS COUNTS AND PROPORTIONS FOR THE
LATENT CLASSES
BASED ON ESTIMATED POSTERIOR PROBABILITIES

    Latent
   Classes

       1        6256.52283          0.22504
       2        2512.95967          0.09039
       3        3788.57316          0.13627
       4        7106.88021          0.25562
       5        3150.58120          0.11332
       6        4986.48292          0.17936
```

```
FINAL CLASS COUNTS AND PROPORTIONS FOR THE
LATENT CLASSES
BASED ON THEIR MOST LIKELY LATENT CLASS
MEMBERSHIP

Class Counts and Proportions

  Latent
 Classes

    1        6431        0.23131
    2        2290        0.08237
    3        3705        0.13326
    4        7760        0.27912
    5        3166        0.11388
    6        4450        0.16006

CLASSIFICATION QUALITY

   Entropy                     0.878

Average Latent Class Probabilities for Most Likely Latent
Class Membership (Row)
by Latent Class (Column)

          1       2       3       4       5       6

   1   0.928   0.063   0.001   0.007   0.000   0.001
   2   0.093   0.900   0.001   0.001   0.001   0.004
   3   0.000   0.000   0.996   0.001   0.001   0.001
   4   0.008   0.002   0.004   0.852   0.002   0.132
   5   0.001   0.001   0.007   0.002   0.981   0.007
   6   0.002   0.005   0.008   0.099   0.006   0.880

Classification Probabilities for the Most Likely Latent Class
Membership (Column)
by Latent Class (Row)

          1       2       3       4       5       6

   1   0.954   0.034   0.000   0.009   0.001   0.001
   2   0.161   0.820   0.001   0.007   0.001   0.010
   3   0.001   0.001   0.974   0.009   0.006   0.009
   4   0.006   0.000   0.000   0.930   0.001   0.062
   5   0.000   0.001   0.001   0.004   0.986   0.008
   6   0.002   0.002   0.001   0.206   0.005   0.785

Logits for the Classification Probabilities for the Most Likely
Latent Class Membership (Column)
by Latent Class (Row)

            1       2       3       4       5       6

   1     6.500   3.171  -1.890   1.869  -0.946   0.000
   2     2.821   4.447  -2.894  -0.260  -2.318   0.000
   3    -2.122  -2.648   4.647  -0.068  -0.441   0.000
   4    -2.335  -5.695  -4.844   2.710  -4.048   0.000
   5    -3.300  -2.693  -1.970  -0.648   4.827   0.000
   6    -6.242  -6.076  -6.711  -1.340  -5.134   0.000

MODEL RESULTS

                                          Two-Tailed
                 Estimate       S.E.  Est./S.E.    P-Value

Latent Class 1

 SLPNSSNC WITH
    WORRYNC          -0.223      0.017    -12.898      0.000
    SOLC             -0.033      0.003    -12.984      0.000
    SLPDURC           0.199      0.017     11.802      0.000

 WORRYNC  WITH
    SOLC              0.138      0.002     56.172      0.000
    SLPDURC          -0.911      0.017    -52.271      0.000

 SOLC     WITH
    SLPDURC          -0.131      0.003    -48.309      0.000

 Means
    SLPNSSNC          0.787      0.009     86.467      0.000
    WORRYNC           0.855      0.004    196.544      0.000
    SOLC             -1.055      0.000  *********      0.000
    SLPDURC          -0.752      0.014    -55.549      0.000

 Variances
    SLPNSSNC          0.473      0.005     88.730      0.000
    WORRYNC           0.988      0.020     49.163      0.000
    SOLC              0.020      0.001     36.573      0.000
    SLPDURC           1.115      0.022     49.574      0.000

Latent Class 2

 SLPNSSNC WITH
    WORRYNC           0.107      0.027      3.924      0.000
    SOLC             -0.016      0.007     -2.437      0.015
    SLPDURC           0.066      0.025      2.581      0.010

 WORRYNC  WITH
    SOLC             -0.076      0.004    -17.336      0.000
    SLPDURC           0.208      0.040      5.254      0.000

 SOLC     WITH
    SLPDURC          -0.058      0.006     -9.098      0.000

 Means
    SLPNSSNC          0.679      0.017     39.866      0.000
    WORRYNC          -0.080      0.027     -2.949      0.003
    SOLC             -1.054      0.000  -6158.720      0.000
    SLPDURC           0.157      0.022      6.983      0.000

 Variances
    SLPNSSNC          0.473      0.005     88.730      0.000
    WORRYNC           0.988      0.020     49.163      0.000
    SOLC              0.020      0.001     36.573      0.000
    SLPDURC           1.115      0.022     49.574      0.000

Latent Class 3

 SLPNSSNC WITH
    WORRYNC           0.097      0.015      6.565      0.000
    SOLC              0.029      0.003      8.616      0.000
    SLPDURC          -0.125      0.016     -7.852      0.000

 WORRYNC  WITH
    SOLC              0.049      0.005      9.155      0.000
    SLPDURC          -0.497      0.022    -22.271      0.000

 SOLC     WITH
    SLPDURC          -0.067      0.004    -15.090      0.000

 Means
    SLPNSSNC          1.101      0.011    101.666      0.000
    WORRYNC          -0.580      0.015    -37.798      0.000
    SOLC              0.910      0.000   9470.622      0.000
    SLPDURC           0.494      0.016     31.282      0.000

 Variances
    SLPNSSNC          0.473      0.005     88.730      0.000
    WORRYNC           0.988      0.020     49.163      0.000
```

```
    SOLC               0.020      0.001     36.573      0.000
    SLPDURC            1.115      0.022     49.574      0.000

Latent Class 4

 SLPNSSNC WITH
    WORRYNC           -0.145      0.023     -6.286      0.000
    SOLC               0.023      0.003      6.466      0.000
    SLPDURC            0.132      0.021      6.144      0.000

 WORRYNC  WITH
    SOLC              -0.133      0.002    -54.161      0.000
    SLPDURC           -0.828      0.018    -46.563      0.000

 SOLC     WITH
    SLPDURC            0.120      0.003     43.259      0.000

 Means
    SLPNSSNC           0.972      0.010     94.865      0.000
    WORRYNC            0.535      0.007     80.781      0.000
    SOLC              -0.071      0.000   -836.073      0.000
    SLPDURC           -0.373      0.013    -27.768      0.000

 Variances
    SLPNSSNC           0.473      0.005     88.730      0.000
    WORRYNC            0.988      0.020     49.163      0.000
    SOLC               0.020      0.001     36.573      0.000
    SLPDURC            1.115      0.022     49.574      0.000

Latent Class 5

 SLPNSSNC WITH
    WORRYNC            0.014      0.013      1.147      0.251
    SOLC              -0.001      0.001     -2.245      0.025
    SLPDURC           -0.004      0.014     -0.268      0.789

 WORRYNC  WITH
    SOLC              -0.011      0.001    -11.157      0.000
    SLPDURC           -0.407      0.020    -20.611      0.000

 SOLC     WITH
    SLPDURC            0.004      0.001      4.415      0.000

 Means
    SLPNSSNC           1.184      0.012     97.778      0.000
    WORRYNC           -1.338      0.019    -71.482      0.000
    SOLC               2.133      0.008    279.403      0.000
    SLPDURC            0.900      0.020     46.072      0.000

 Variances
    SLPNSSNC           0.473      0.005     88.730      0.000
    WORRYNC            0.988      0.020     49.163      0.000
    SOLC               0.020      0.001     36.573      0.000
    SLPDURC            1.115      0.022     49.574      0.000

Latent Class 6

 SLPNSSNC WITH
    WORRYNC            0.024      0.020      1.194      0.233
    SOLC               0.004      0.004      1.007      0.314
    SLPDURC            0.002      0.017      0.121      0.904

 WORRYNC  WITH
    SOLC               0.082      0.003     25.708      0.000
    SLPDURC           -0.318      0.024    -13.211      0.000

 SOLC     WITH
    SLPDURC           -0.063      0.005    -13.511      0.000

 Means
    SLPNSSNC           0.901      0.013     68.010      0.000
    WORRYNC           -0.509      0.017    -30.758      0.000
    SOLC              -0.072      0.000   -768.888      0.000
    SLPDURC            0.471      0.017     27.896      0.000

 Variances
    SLPNSSNC           0.473      0.005     88.730      0.000
    WORRYNC            0.988      0.020     49.163      0.000
    SOLC               0.020      0.001     36.573      0.000
    SLPDURC            1.115      0.022     49.574      0.000

Categorical Latent Variables

 Means
    C#1                0.227      0.028      8.058      0.000
    C#2               -0.685      0.041    -16.645      0.000
    C#3               -0.275      0.028     -9.843      0.000
    C#4                0.354      0.035     10.135      0.000
    C#5               -0.459      0.029    -15.917      0.000

QUALITY OF NUMERICAL RESULTS

     Condition Number for the Information Matrix
0.573E-10
       (ratio of smallest to largest eigenvalue)

TECHNICAL 11 OUTPUT

     Random Starts Specifications for the k-1 Class Analysis
Model
        Number of initial stage random starts                8000
        Number of final stage optimizations                  2000

     VUONG-LO-MENDELL-RUBIN LIKELIHOOD RATIO
TEST FOR 5 (H0) VERSUS 6 CLASSES

          H0 Loglikelihood Value                    -107985.460
          2 Times the Loglikelihood Difference          4289.315
          Difference in the Number of Parameters              11
          Mean                                  -106396860.944
          Standard Deviation                      150385030.149
          P-Value                                         0.2396

     LO-MENDELL-RUBIN ADJUSTED LRT TEST

          Value                                        4251.544
          P-Value                                         0.2396

TECHNICAL 14 OUTPUT

     Random Starts Specifications for the k-1 Class Analysis
Model
        Number of initial stage random starts                8000
        Number of final stage optimizations                  2000

     Random Starts Specification for the k-1 Class Model for
Generated Data
        Number of initial stage random starts                  16
        Number of final stage optimizations                     8
     Random Starts Specification for the k Class Model for
Generated Data
        Number of initial stage random starts                 400
        Number of final stage optimizations                    80
     Number of bootstrap draws requested                      500

     PARAMETRIC BOOTSTRAPPED LIKELIHOOD RATIO
TEST FOR 5 (H0) VERSUS 6 CLASSES

          H0 Loglikelihood Value                    -107985.460
          2 Times the Loglikelihood Difference          4289.315
```

```
          Difference in the Number of Parameters              11
          Approximate P-Value                         0.0000
          Successful Bootstrap Draws                     493

     WARNING:  OF THE 493 BOOTSTRAP DRAWS, 452
DRAWS HAD BOTH A SMALLER LRT VALUE THAN THE
     OBSERVED LRT VALUE AND NOT A REPLICATED
BEST LOGLIKELIHOOD VALUE FOR THE 6-CLASS
MODEL.
     THIS MEANS THAT THE P-VALUE MAY NOT BE
TRUSTWORTHY DUE TO LOCAL MAXIMA.
     INCREASE THE NUMBER OF RANDOM STARTS
USING THE LRTSTARTS OPTION.

     WARNING:  7 OUT OF 500 BOOTSTRAP DRAWS DID
NOT CONVERGE.
     INCREASE THE NUMBER OF RANDOM STARTS
USING THE LRTSTARTS OPTION.

PLOT INFORMATION

The following plots are available:

  Histograms (sample values)
  Scatterplots (sample values)
  Sample means
  Estimated means, medians, modes, and percentiles
  Sample and estimated means
  Observed individual values
  Estimated means and observed individual values
  Estimated overall and class-specific distributions

SAVEDATA INFORMATION

  Save file
    Step2_LPA6M5.dat

  Order and format of variables

    SLPNSSNC       F10.3
    WORRYNC        F10.3
    SOLC           F10.3
    SLPDURC        F10.3
    CPROB1         F10.3
    CPROB2         F10.3
    CPROB3         F10.3
    CPROB4         F10.3
    CPROB5         F10.3
    CPROB6         F10.3
    C              F10.3
    ID             I6

  Save file format
    11F10.3 I6

  Save file record length    10000

  Save missing symbol        *

DIAGRAM INFORMATION

  Mplus diagrams are currently not available for Mixture
analysis.
  No diagram output was produced.

     Beginning Time:   13:26:45
        Ending Time:   08:20:25
       Elapsed Time:  114:53:40

MUTHEN & MUTHEN
3463 Stoner Ave.
Los Angeles, CA  90066

Tel: (310) 391-9971
Fax: (310) 391-8971
Web: www.StatModel.com
Support: Support@StatModel.com

Copyright (c) 1998-2021 Muthen & Muthen
```

Secondary Analysis in R

Set graphic theme

```
month_theme <- theme(axis.text.x = element_text(angle = 45, hjust=1, vjust=1, size=7,
    family="Serif"),
                axis.title.x = element_blank(),
                strip.text.x = element_text(size = 10,
    family="Serif"),
                axis.text.y = element_text(size=7,
    family="Serif"),
                axis.title.y = element_text(size=7,
    family="Serif"),
                legend.title = element_blank(),
                legend.text = element_text(size=7,
    family="Serif"),
                legend.position="right",
    panel.grid.major.y = element_blank(),
   panel.grid.minor.y = element_blank())

set_theme(
  geom.outline.color = "white",
  geom.outline.size = 0,
  geom.label.size = 2,
  geom.label.color = "#040540",
  axis.textcolor = "#040540",
  axis.title.color = "#040540",
  legend.color = "#040540",
  legend.title.color = "#040540",
  base = theme_bw()
)
```

Apply classes

```
# Read Mplus data from txt file
classData<-read.table("Step2_LPA5M5.txt", header=FALSE,na.strings=c("", "*"))

## Names of columns - taken from MPlus output
names(classData) <- c("SLPNSSNC", "WORRYNC", "SOLC", "SLPDURC", "CPROB1", "CPROB2", "CPROB3", "CPROB4", "CPR
OB5", "C", "ID")

data$ID <- data$X

## Merge the two
merged <- merge(classData, data, by="ID")

## Probability
probability <- merged[,c("CPROB1", "CPROB2", "CPROB3", "CPROB4", "CPROB5")] # select the probability columns
probability$max<-apply(probability, 1, max) # obtain the max value across the columns

# Add the 'max' column into the data as "weight"
merged$weight <- probability$max

## Create class column to text:
merged$Class <- ifelse(merged$C==1,"Class1",
            ifelse(merged$C==2, "Class2",
            ifelse(merged$C==3, "Class3",
            ifelse(merged$C==4,"Class4", "Class5"))))

merged$Class <- as.factor(merged$Class)

table(is.na(merged$Class))

##
## FALSE
## 27802
```

```
merged[is.na(merged["Class"]),"ID"]
```

Double check missingness & describe classes

```
predictors <- c('Gender_dummy', 'YEARGROUP_cat_dummy', 'SCI_02_binary')

for(x in predictors){
  merged[,x] <- as.character(merged[,x])
}


# get descriptives
table1::table1(~ Gender_dummy + Gender + YEARGROUP_cat_dummy +
                YEARGROUP_cat + SCI_02_binary +
                SCI_02 +
                sleepiness_num +
                worry_stops_sleep_num +
                sol +
                sch_sleep_duration|Class,
              data = merged,
              topclass="Rtable1-zebra")

## Get nicer `table1` .docx output by simply installing the `flextable` package

##                              Class1          Class2          Class3
## 1                           (N=1037)        (N=4825)        (N=1250)
## 2          Gender_dummy
## 3                   0      681 (65.7%)     2982 (61.8%)     772 (61.8%)
## 4                   1      356 (34.3%)     1843 (38.2%)     478 (38.2%)
## 5                Gender
## 6                Female       681 (65.7%)     2982 (61.8%)     772 (61.8%)
## 7                  Male      356 (34.3%)     1843 (38.2%)     478 (38.2%)
## 8    YEARGROUP_cat_dummy
## 9                   0      367 (35.4%)     1426 (29.6%)     181 (14.5%)
## 10                  1      448 (43.2%)     2438 (50.5%)     628 (50.2%)
## 11                  2      222 (21.4%)      961 (19.9%)     441 (35.3%)
## 12        YEARGROUP_cat
## 13          Year 10-13      222 (21.4%)      961 (19.9%)     441 (35.3%)
## 14            Year 5-6      367 (35.4%)     1426 (29.6%)     181 (14.5%)
## 15            Year 7-9      448 (43.2%)     2438 (50.5%)     628 (50.2%)
## 16        SCI_02_binary
## 17           good sleep      316 (30.5%)     4823 (100.0%)      1250 (100%)
## 18    probable insomnia      721 (69.5%)          2 (0.0%)           0 (0%)
## 19               SCI_02
## 20            Mean (SD)      1.97 (0.895)      4.27 (0.762)      5.39 (1.24)
## 21    Median [Min, Max]    2.00 [0, 3.00] 4.00 [2.00, 6.00] 5.00 [4.00, 8.00]
## 22       sleepiness_num
## 23            Mean (SD)      1.96 (0.593)      2.21 (0.655)      4.15 (0.378)
## 24    Median [Min, Max] 2.00 [1.00, 3.00] 2.00 [1.00, 3.00] 4.00 [3.00, 5.00]
## 25              Missing        23 (2.2%)       187 (3.9%)           0 (0%)
## 26 worry_stops_sleep_num
## 27            Mean (SD)       3.38 (1.14)       2.73 (1.03)       2.84 (1.14)
## 28    Median [Min, Max] 4.00 [1.00, 5.00] 3.00 [1.00, 5.00] 3.00 [1.00, 5.00]
## 29              Missing        41 (4.0%)       239 (5.0%)        61 (4.9%)
## 30                  sol
## 31            Mean (SD)      1.35 (0.565)      1.01 (0.559)     0.848 (0.589)
## 32    Median [Min, Max]    1.49 [0, 2.00]   0.964 [0, 2.00]   0.688 [0, 2.00]
## 33              Missing        40 (3.9%)       198 (4.1%)        57 (4.6%)
## 34   sch_sleep_duration
## 35            Mean (SD)       7.14 (1.69)       7.58 (1.54)       7.27 (1.56)
## 36    Median [Min, Max] 7.19 [3.00, 11.8] 7.63 [3.04, 12.0] 7.27 [3.00, 12.0]
## 37              Missing      144 (13.9%)      649 (13.5%)      161 (12.9%)
##              Class4          Class5          Overall
## 1          (N=2335)        (N=18355)        (N=27802)
## 2
## 3      1701 (72.8%)     9072 (49.4%)     15208 (54.7%)
## 4       634 (27.2%)     9283 (50.6%)     12594 (45.3%)
## 5
## 6      1701 (72.8%)     9072 (49.4%)     15208 (54.7%)
```

```
## 7       634 (27.2%)      9283 (50.6%)     12594 (45.3%)
## 8
## 9       319 (13.7%)      5242 (28.6%)      7535 (27.1%)
## 10      1244 (53.3%)      9837 (53.6%)     14595 (52.5%)
## 11       772 (33.1%)      3276 (17.8%)      5672 (20.4%)
## 12
## 13       772 (33.1%)      3276 (17.8%)      5672 (20.4%)
## 14       319 (13.7%)      5242 (28.6%)      7535 (27.1%)
## 15      1244 (53.3%)      9837 (53.6%)     14595 (52.5%)
## 16
## 17       473 (20.3%)      18355 (100%)     25217 (90.7%)
## 18      1862 (79.7%)           0 (0%)       2585 (9.3%)
## 19
## 20      1.57 (0.994)      7.22 (0.763)       5.96 (2.11)
## 21    2.00 [0, 3.00] 7.00 [6.00, 8.00]    7.00 [0, 8.00]
## 22
## 23      4.06 (0.656)      1.65 (0.628)       2.07 (1.02)
## 24 4.00 [3.00, 5.00] 2.00 [1.00, 3.00] 2.00 [1.00, 5.00]
## 25         95 (4.1%)        374 (2.0%)        679 (2.4%)
## 26
## 27       3.65 (1.03)      1.95 (0.873)       2.32 (1.10)
## 28 4.00 [1.00, 5.00] 2.00 [1.00, 5.00] 2.00 [1.00, 5.00]
## 29         89 (3.8%)        609 (3.3%)       1039 (3.7%)
## 30
## 31      1.38 (0.590)     0.632 (0.478)     0.795 (0.572)
## 32    1.51 [0, 2.00]   0.492 [0, 2.00]   0.560 [0, 2.00]
## 33         92 (3.9%)        629 (3.4%)       1016 (3.7%)
## 34
## 35       6.61 (1.62)       8.24 (1.40)       7.90 (1.56)
## 36 6.64 [3.00, 11.9] 8.28 [3.00, 12.0] 7.99 [3.00, 12.0]
## 37       320 (13.7%)      2428 (13.2%)      3702 (13.3%)

# get descriptives
table1::table1(~ Class,
               data = merged,
               topclass="Rtable1-zebra")

## Get nicer `table1` .docx output by simply installing the `flextable` package

##                  Overall
## 1              (N=27802)
## 2    Class
## 3   Class1   1037 (3.7%)
## 4   Class2  4825 (17.4%)
## 5   Class3   1250 (4.5%)
## 6   Class4   2335 (8.4%)
## 7   Class5 18355 (66.0%)
```

Graph classes

```
# create a percentage variable
merged$Class2 <- factor(merged$Class, levels = c("Class1", "Class2", "Class3", "Class4", "Class5"),
                              labels = c("Poor sleepers (3.7%)",
                                 "Moderate sleepers (17.4%)",
                                 "Moderate sleepy sleepers (4.5%)",
                                 "Poor sleepy sleepers (8.4%)",
                                 "Good sleepers (66.0%)"))

merged$SCI_02_S <- scale(merged$SCI_02, center = TRUE, scale = TRUE)
merged$sleepiness_S <- scale(merged$sleepiness_num, center = TRUE, scale = TRUE)
merged$worry_S <- scale(merged$worry_stops_sleep_num, center = TRUE, scale = TRUE)
merged$sol_S <- scale(merged$sol, center = TRUE, scale = TRUE)
merged$dur_S <- scale(merged$sch_sleep_duration, center = TRUE, scale = TRUE)

# separate graphs for class
# Select only columns that are needed:
data2 <- merged[,c("Class2", "SCI_02_S", "sleepiness_S", "worry_S", "sol_S", "dur_S", "ID")]
```

```
# Summary
summary(data2)

##                          Class2          SCI_02_S.V1       sleepiness_S.V1
##  Poor sleepers (3.7%)           : 1037   Min.   :-2.8248292   Min.   :-1.0538
##  Moderate sleepers (17.4%)      : 4825   1st Qu.:-0.4538482   1st Qu.:-1.0538
##  Moderate sleepy sleepers (4.5%): 1250   Median : 0.4945442   Median :-0.0717
##  Poor sleepy sleepers (8.4%)    : 2335   Mean   : 0.0000000   Mean   : 0.0000
##  Good sleepers (66.0%)          :18355   3rd Qu.: 0.9687404   3rd Qu.:-0.0717
##                                          Max.   : 0.9687404   Max.   : 2.8745
##                                                               NA's   :679
##    worry_S.V1          sol_S.V1          dur_S.V1           ID
##  Min.   :-1.2049   Min.   :-1.3912   Min.   :-3.152   Min.   :    1
##  1st Qu.:-1.2049   1st Qu.:-0.7475   1st Qu.:-0.612   1st Qu.: 6951
##  Median :-0.2923   Median :-0.4116   Median : 0.056   Median :13902
##  Mean   : 0.0000   Mean   : 0.0000   Mean   : 0.000   Mean   :13902
##  3rd Qu.: 0.6202   3rd Qu.: 0.6799   3rd Qu.: 0.683   3rd Qu.:20852
##  Max.   : 2.4452   Max.   : 2.1073   Max.   : 2.619   Max.   :27802
##  NA's   :1039      NA's   :1016      NA's   :3702

class(data2)

## [1] "data.frame"

## Long data: just indicators
long_data <- pivot_longer(data = data2,
  cols = sleepiness_S:dur_S,
  names_to ="scale",
  values_to = "measurement")

long_data$scale <- factor(long_data$scale, levels = c("sleepiness_S", "worry_S", "sol_S", "dur_S"),
                          labels = c("Sleepiness", "Worry disrupts sleep", "SOL", "Sleep duration"))

summary(long_data)

##                          Class2          SCI_02_S.V1            ID
##  Poor sleepers (3.7%)           : 4148   Min.   :-2.8248292   Min.   :    1
##  Moderate sleepers (17.4%)      :19300   1st Qu.:-0.4538482   1st Qu.: 6951
##  Moderate sleepy sleepers (4.5%): 5000   Median : 0.4945442   Median :13902
##  Poor sleepy sleepers (8.4%)    : 9340   Mean   : 0.0000000   Mean   :13902
##  Good sleepers (66.0%)          :73420   3rd Qu.: 0.9687404   3rd Qu.:20852
##                                          Max.   : 0.9687404   Max.   :27802
##
##                   scale        measurement.V1
##  Sleepiness          :27802   Min.   :-3.152
##  Worry disrupts sleep:27802   1st Qu.:-0.810
##  SOL                 :27802   Median :-0.072
##  Sleep duration      :27802   Mean   : 0.000
##                               3rd Qu.: 0.620
##                               Max.   : 2.875
##                               NA's   :6436

lineFig <- ggplot(long_data, aes(x=scale, y=measurement, group=Class2, colour=Class2, fill=Class2)) +
  stat_summary(fun=mean, geom="point") + stat_summary(fun=mean, geom="line") + stat_summary(fun.data = mean_cl_normal
, geom="ribbon", alpha = 0.3, colour=NA) + ylab(label= 'Standardized scores') + scale_colour_viridis_d() + scale_fill_viridis_d()
+ month_theme + coord_cartesian(ylim = c(-2,2))

lineFig
```

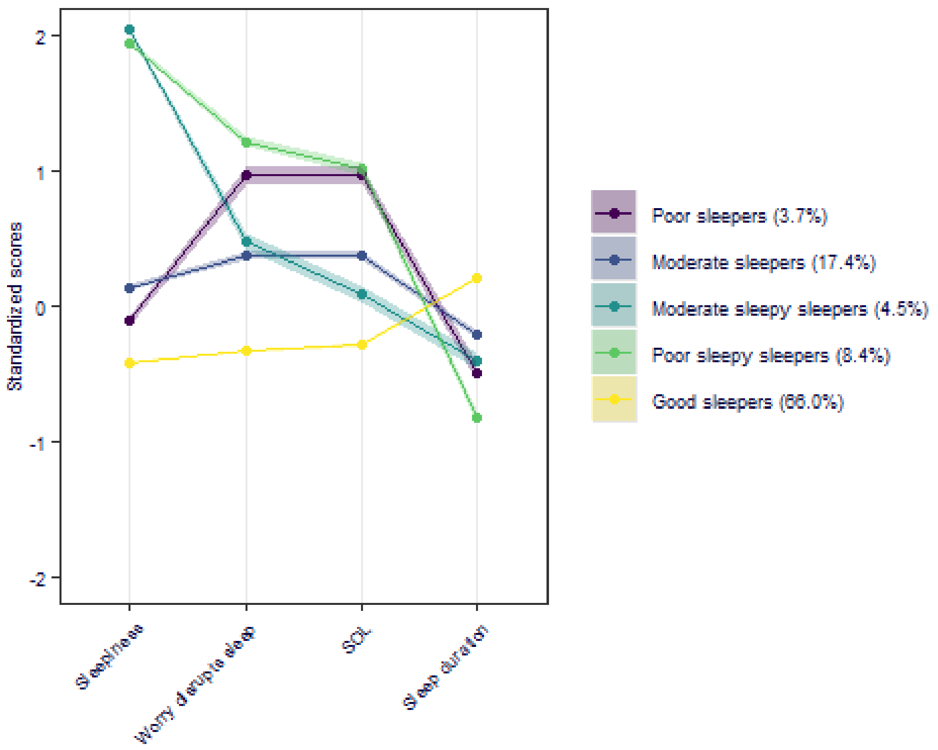

Investigate relationship with SCI-02

```
# graph
## Long data: with SCI-02
long_data <- pivot_longer(data = data2,
  cols = SCI_02_S:dur_S,
  names_to ="scale",
  values_to = "measurement")

long_data$scale <- factor(long_data$scale, levels = c("sleepiness_S", "worry_S", "sol_S", "dur_S", "SCI_02_S"),
                          labels = c("Sleepiness", "Worry before sleep", "SOL", "Sleep duration", "SCI_02"))

summary(long_data)

##                              Class2          ID
##  Poor sleepers (3.7%)           : 5185   Min.   :    1
##  Moderate sleepers (17.4%)      :24125   1st Qu.: 6951
##  Moderate sleepy sleepers (4.5%): 6250   Median :13902
##  Poor sleepy sleepers (8.4%)    :11675   Mean   :13902
##  Good sleepers (66.0%)          :91775   3rd Qu.:20852
##                                          Max.   :27802
##
##                 scale         measurement.V1
##  Sleepiness        :27802   Min.   :-3.152
##  Worry before sleep:27802   1st Qu.:-0.780
##  SOL               :27802   Median :-0.072
##  Sleep duration    :27802   Mean   : 0.000
##  SCI_02            :27802   3rd Qu.: 0.620
##                             Max.   : 2.875
##                             NA's   :6436

ggplot(long_data, aes(x=scale, y=measurement, group=Class2, colour=Class2, fill=Class2)) +
  stat_summary(fun=mean, geom="point")+stat_summary(fun=mean, geom="line")+ stat_summary(fun.data = mean_cl_normal,
geom="ribbon", alpha = 0.3, colour=NA) + ylab(label= 'Standardized scores') + scale_colour_viridis_d() + scale_fill_viridis_d() +
month_theme
```

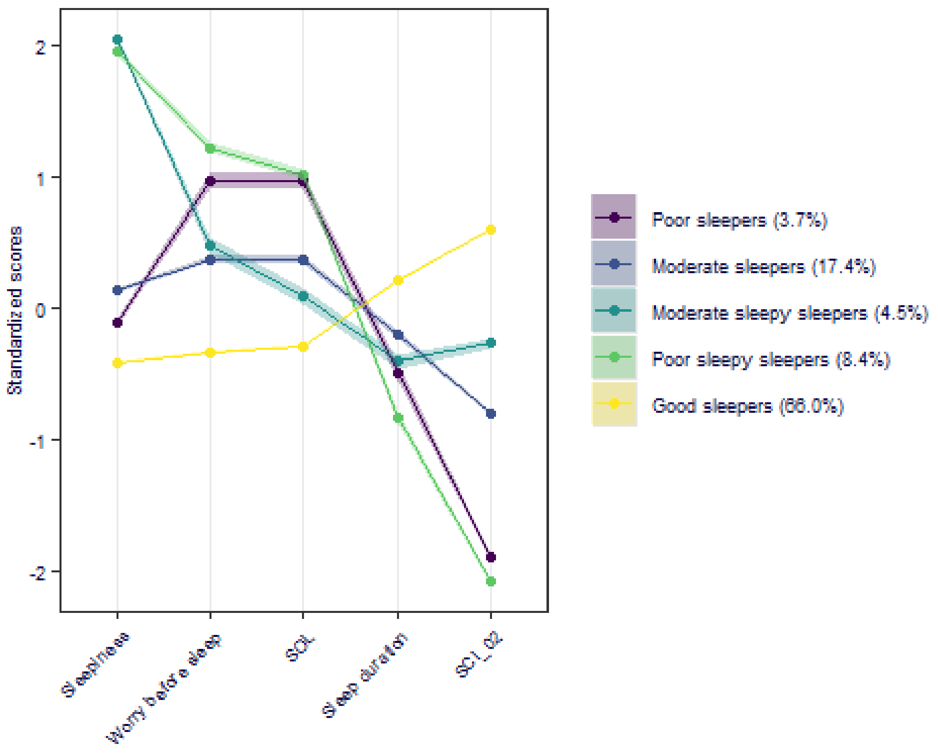


```
# anova
SCI_02_comp <- aov(SCI_02 ~ Class2, data = merged)

summary(SCI_02_comp)

##              Df Sum Sq Mean Sq F value Pr(>F)
## Class2        4 105084   26271   39364 <2e-16 ***
## Residuals   27797  18551       1
## ---
## Signif. codes:  0 '***' 0.001 '**' 0.01 '*' 0.05 '.' 0.1 ' ' 1

pairwise.t.test(merged$SCI_02, merged$Class2,
                p.adjust.method = "bonferroni")

##
##  Pairwise comparisons using t tests with pooled SD
##
## data:  merged$SCI_02 and merged$Class2
##
##                               Poor sleepers (3.7%) Moderate sleepers (17.4%)
## Moderate sleepers (17.4%)      <2e-16               -
## Moderate sleepy sleepers (4.5%) <2e-16              <2e-16
## Poor sleepy sleepers (8.4%)    <2e-16               <2e-16
## Good sleepers (66.0%)          <2e-16               <2e-16
##                               Moderate sleepy sleepers (4.5%)
## Moderate sleepers (17.4%)      -
## Moderate sleepy sleepers (4.5%) -
## Poor sleepy sleepers (8.4%)    <2e-16
## Good sleepers (66.0%)          <2e-16
##                               Poor sleepy sleepers (8.4%)
## Moderate sleepers (17.4%)      -
## Moderate sleepy sleepers (4.5%) -
## Poor sleepy sleepers (8.4%)    -
## Good sleepers (66.0%)          <2e-16
##
## P value adjustment method: bonferroni
```

Logistic regression

```
# Select only columns that are needed:
data3 <- merged[,c("Class", "Gender_dummy", "YEARGROUP_cat_dummy", "weight", "ID")]

predictors <- c('Gender_dummy', 'YEARGROUP_cat_dummy')

for(x in predictors){
  data3[,x] <- as.character(data3[,x])
}

# Summary
summary(data3)

##     Class       Gender_dummy       YEARGROUP_cat_dummy     weight
##  Class1: 1037   Length:27802       Length:27802        Min.   :0.3510
##  Class2: 4825   Class :character   Class :character    1st Qu.:0.8850
##  Class3: 1250   Mode  :character   Mode  :character    Median :0.9950
##  Class4: 2335                                          Mean   :0.9157
##  Class5:18355                                          3rd Qu.:1.0000
##                                                        Max.   :1.0000
##        ID
##  Min.   :    1
##  1st Qu.: 6951
##  Median :13902
##  Mean   :13902
##  3rd Qu.:20852
##  Max.   :27802

## Long data
data4 = mlogit.data(data3, choice = "Class", shape="wide")

# Model:
model_LR <- mlogit(Class ~ 1 | Gender_dummy + YEARGROUP_cat_dummy, data=data4, reflevel =  "Class5")

#summary
summary(model_LR)

##
## Call:
## mlogit(formula = Class ~ 1 | Gender_dummy + YEARGROUP_cat_dummy,
##     data = data4, reflevel = "Class5", method = "nr")
##
## Frequencies of alternatives:choice
##   Class5   Class1   Class2   Class3   Class4
## 0.660204 0.037299 0.173549 0.044961 0.083987
##
## nr method
## 7 iterations, 0h:0m:7s
## g'(-H)^-1g = 1.04E-05
## successive function values within tolerance limits
##
## Coefficients :
##                             Estimate Std. Error  z-value  Pr(>|z|)
## (Intercept):Class1          -2.349727   0.059826 -39.2761 < 2.2e-16 ***
## (Intercept):Class2          -1.061812   0.033331 -31.8564 < 2.2e-16 ***
## (Intercept):Class3          -3.137894   0.079848 -39.2985 < 2.2e-16 ***
## (Intercept):Class4          -2.389418   0.059998 -39.8249 < 2.2e-16 ***
## Gender_dummy1:Class1        -0.684501   0.067160 -10.1921 < 2.2e-16 ***
## Gender_dummy1:Class2        -0.506638   0.033142 -15.2869 < 2.2e-16 ***
## Gender_dummy1:Class3        -0.477822   0.060346  -7.9180 2.442e-15 ***
## Gender_dummy1:Class4        -0.984424   0.049116 -20.0429 < 2.2e-16 ***
## YEARGROUP_cat_dummy1:Class1 -0.458847   0.072667  -6.3144 2.713e-10 ***
## YEARGROUP_cat_dummy1:Class2 -0.114792   0.037669  -3.0474  0.002308 **
## YEARGROUP_cat_dummy1:Class3  0.594139   0.086180   6.8942 5.417e-12 ***
## YEARGROUP_cat_dummy1:Class4  0.691392   0.065404  10.5711 < 2.2e-16 ***
## YEARGROUP_cat_dummy2:Class1 -0.062002   0.088141  -0.7034  0.481785
## YEARGROUP_cat_dummy2:Class2  0.053350   0.047540   1.1222  0.261776
```

```
## YEARGROUP_cat_dummy2:Class3  1.339768   0.091165  14.6961 < 2.2e-16 ***
## YEARGROUP_cat_dummy2:Class4  1.313035   0.070679  18.5775 < 2.2e-16 ***
## ---
## Signif. codes:  0 '***' 0.001 '**' 0.01 '*' 0.05 '.' 0.1 ' ' 1
##
## Log-Likelihood: -28462
## McFadden R^2:  0.023353
## Likelihood ratio test : chisq = 1361.2 (p.value = < 2.22e-16)

exp(coefficients(model_LR)) ## risk ratio

##         (Intercept):Class1         (Intercept):Class2
##                 0.09539524                 0.34582845
##         (Intercept):Class3         (Intercept):Class4
##                 0.04337404                 0.09168299
##       Gender_dummy1:Class1       Gender_dummy1:Class2
##                 0.50434183                 0.60251763
##       Gender_dummy1:Class3       Gender_dummy1:Class4
##                 0.62013258                 0.37365447
## YEARGROUP_cat_dummy1:Class1 YEARGROUP_cat_dummy1:Class2
##                 0.63201174                 0.89155141
## YEARGROUP_cat_dummy1:Class3 YEARGROUP_cat_dummy1:Class4
##                 1.81147094                 1.99649191
## YEARGROUP_cat_dummy2:Class1 YEARGROUP_cat_dummy2:Class2
##                 0.93988144                 1.05479873
## YEARGROUP_cat_dummy2:Class3 YEARGROUP_cat_dummy2:Class4
##                 3.81815842                 3.71743742

exp(confint.default(model_LR))

##                                  2.5 %    97.5 %
## (Intercept):Class1          0.08484043 0.1072632
## (Intercept):Class2          0.32395830 0.3691750
## (Intercept):Class3          0.03709057 0.0507220
## (Intercept):Class4          0.08151139 0.1031239
## Gender_dummy1:Class1        0.44213823 0.5752967
## Gender_dummy1:Class2        0.56462379 0.6429547
## Gender_dummy1:Class3        0.55095677 0.6979938
## Gender_dummy1:Class4        0.33936156 0.4114127
## YEARGROUP_cat_dummy1:Class1 0.54811380 0.7287517
## YEARGROUP_cat_dummy1:Class2 0.82809917 0.9598656
## YEARGROUP_cat_dummy1:Class3 1.52994298 2.1448034
## YEARGROUP_cat_dummy1:Class4 1.75628654 2.2695499
## YEARGROUP_cat_dummy2:Class1 0.79076449 1.1171179
## YEARGROUP_cat_dummy2:Class2 0.96095520 1.1578067
## YEARGROUP_cat_dummy2:Class3 3.19340534 4.5651373
## YEARGROUP_cat_dummy2:Class4 3.23654718 4.2697789
```

Produce a forest graph

```
oddsSleep <- broom::tidy(model_LR) # extract model estimates into a separate DF
oddsSleep$OR <- exp(coefficients(model_LR)) # extract and save ORs
oddsSleep$CI <- exp(confint.default(model_LR)) # extract and save CIs
oddsSleep$Class <- rep(c("C1",
                   "C2",
                   "C3",
                   "C4"), 4) # create a new variable for class

# might want to adjust these but a place holder for now
oddsSleep$Class <- factor(oddsSleep$Class,
                    levels = c("C1",
                   "C2",
                   "C3",
                   "C4",
                   "C5"),
                   labels = c("Poor sleepers",
                          "Moderate sleepers",
                          "Moderate sleepy sleepers",
                          "Poor sleepy sleepers",
```

```
                              "Good sleepers"))

oddsSleep$term <- gsub("\\:.*","", oddsSleep$term) # remove class name from the effects

oddsSleep <- dplyr::filter(oddsSleep, term!="(Intercept)") # remove intercept values

#write.table(oddsSleep, file="oddsNon-gamers.csv", row.names=F, sep=",")


oddsSleep$effect <- rep(c("Gender: Boy (vs. Girl)",
                          "Year: 7-9 (vs. 5-6)",
                          "Year: 10-13 (vs. 5-6)"),
                        each=4) # create a new variable for class

# Relevel effect factor
oddsSleep$effect <- factor(oddsSleep$effect,
                          levels = c("Gender: Boy (vs. Girl)",
                          "Year: 7-9 (vs. 5-6)",
                          "Year: 10-13 (vs. 5-6)"))

forestStr <- ggplot(oddsSleep, aes(y=effect,x=OR,colour=Class))+
  geom_pointrangeh(aes(xmin=CI[,1],
                       xmax=CI[,2]),
    position=position_dodgev(height=0.75)) + scale_y_discrete(limits=rev) + geom_vline(xintercept=1, linetype=2, color = "grey")
+ labs(x = "Odds Ratio", y=element_blank()) + theme_few() +  theme(axis.text.x = element_text(hjust=0.5, vjust=1, size=7, family
="Serif"), axis.title.x = element_text(size=7, family="Serif"), axis.text.y = element_text(size=7, family="Serif"), axis.title.y = element
_text(size=7, family="Serif"), legend.title = element_blank(), legend.text = element_text(size=7, family="Serif"), legend.position="ri
ght") + scale_x_log10() + scale_colour_viridis_d()

print(forestStr)
```

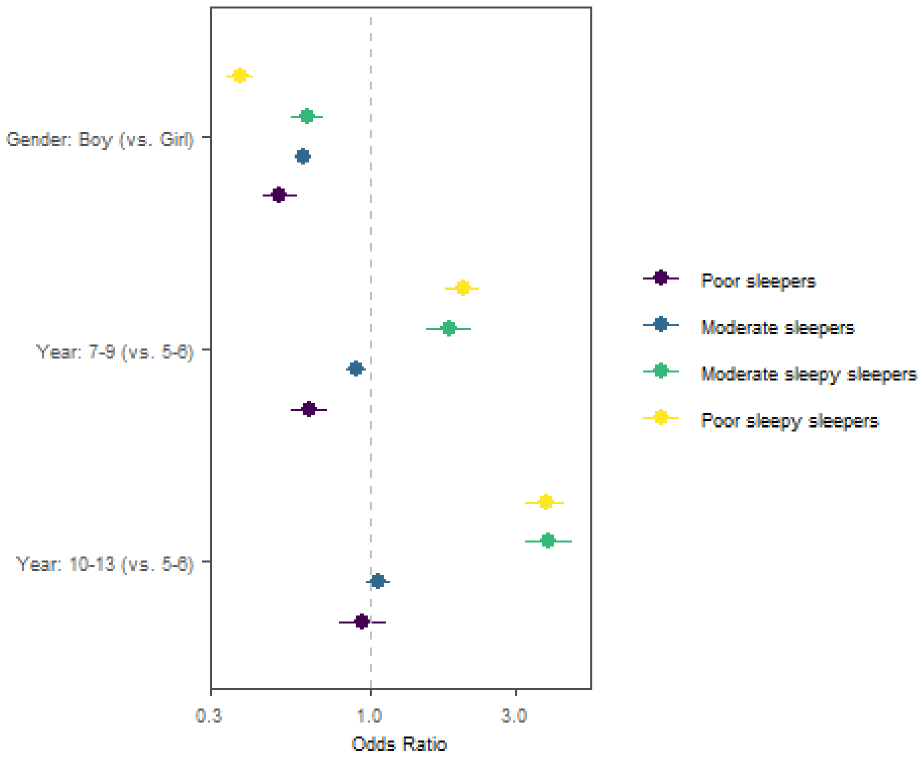

Supplement: Supplementary file 2 — Supplementary Material 2 [file 12889_2024_20310_MOESM2_ESM.pdf]
